# Global Crotonylome Profiling Identifies TaPRXIIB Crotonylation as a Modulator H_2_O_2_ Homeostasis in Wheat Resistance to *Puccinia triticina*

**DOI:** 10.1111/mpp.70288

**Published:** 2026-07-11

**Authors:** Qipeng Wang, Rongna Wang, Tianjie Sun, Qian Wang, Jia Gu, Zelin Niu, Na Liu, Shengfang Han, Dongmei Wang

**Affiliations:** ^1^ State Key Laboratory of North China Crop Improvement and Regulation Baoding China; ^2^ Key Laboratory of Hebei Province for Plant Physiology and Molecular Pathology Baoding China; ^3^ College of Life Sciences Hebei Agricultural University Baoding China

**Keywords:** H_2_O_2_, lysine crotonylation, peroxidases, *Puccinia triticina*, wheat

## Abstract

Wheat food security is severely threatened by *Puccinia triticina* infection. Although lysine crotonylation (Kcr) plays pivotal roles in plant stress responses, its function and regulation in biotic stress remain largely unknown. In this study, we investigated Kcr levels in wheat upon *P. triticina* infection and found that elevated protein crotonylation enhances resistance to *P. triticina*. We first performed a global crotonylome profiling of wheat in response to *P. triticina* infection and identified 3333 quantifiable Kcr sites across 952 proteins. Kcr sites were preferentially flanked by aliphatic amino acids, suggesting a sequence preference for this modification. Functional enrichment and protein–protein interaction (PPI) analyses based on crotonylated differentially expressed proteins (DEPs) further highlighted a strong association between Kcr‐modified antioxidant enzymes and stress response processes. Furthermore, we investigated the modification pattens of two antioxidant enzymes and confirmed that TaPRXIIB negatively regulates wheat resistance to *P. triticina* by modulating H_2_O_2_ homeostasis. We found that crotonylation at K172 of TaPRXIIB disrupts the hydrogen bond between H187 and its prosthetic group, thereby reducing its enzymatic activity. This study presented a global crotonylome profile of wheat in response to *P. triticina* infection and revealed that the Kcr of TaPRXIIB at K172 negatively regulates its enzymatic activity.

## Graphical abstract

Global crotonylome profiling identifies TaPRXIIB^K172^ crotonylation as a key modification that modulates H_2_O_2_ homeostasis, reduces enzyme activity and enhances wheat resistance to leaf rust.

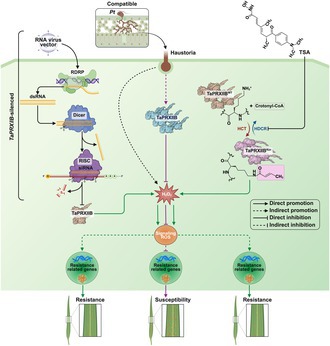

## 1 Introduction

Protein post‐translational modifications (PTMs) regulate protein function by altering their charge, conformation and molecular weight through the addition of chemical groups to specific amino acid residues, thereby expanding their functional diversity and enabling rapid responses to environmental stimuli (Li et al. 2022; Zhang et al. 2023). Lysine post‐translational modifications (K‐PTMs) are covalent chemical modifications that occur on the ε‐amino group (‐NH_3_^+^) of lysine residues, either enzymatically or non‐enzymatically, after protein synthesis (Chen et al. 2007; Xie et al. 2012). As dynamic and reversible regulatory mechanisms, K‐PTMs greatly diversify protein functions (Sabari et al. 2017). With continuous advancements in specific modification antibodies, mass spectrometry‐based platforms and bioinformatics databases, several newly identified types of K‐PTMs, including propionylation (Chen et al. 2007), butyrylation (Chen et al. 2007), succinylation (Zhang et al. 2011), crotonylation (Tan et al. 2011), 2‐hydroxyisobutyrylation (Dai et al. 2014) and lactylation (Zhang et al. 2019), have been reported, and their diversity continues to expand (Zhu et al. 2021). Among these, crotonylation (Kcr), a novel lysine acylation, has emerged as an important regulator in plants, participating in energy metabolism, carbon fixation, amino acid biosynthesis and signal transduction (Subba and Prasad 2021). Recent studies have demonstrated that Kcr modulates plant responses to environmental stresses. Zhang et al. (2023) found that crotonylation of phosphoglycerate kinase (PGK), a key enzyme involved in glycolytic process, stabilises the TaPGK protein structural and enhances cold tolerance in wheat by elevating pyruvate levels (Zhang et al. 2023). A study on cold tolerance in chrysanthemum revealed that Kcr enhances peroxidase (POD) expression and activity under cold stress by inhibiting DgTIL1‐mediated degradation of DgnsLTP, thereby improving the plant's adaptation to low temperatures (Huang et al. 2021). Kcr is not only extensively involved in regulating plant responses to abiotic stress (Wu et al. 2022; Dong and Chen 2022) but also plays a pivotal role in modulating plant–pathogen interactions. In a study on the pathogenicity of *Botrytis cinerea*, 26 Kcr sites were identified that potentially contribute to its infection process (Zhang, Yang, et al. 2020). In jujube under phytoplasma stress, crotonylation levels of the antioxidant enzymes ZjPOD51 and ZjPHGPX2 are significantly elevated, and Kcr modification at lysine 130 and/or 135 was found to enhance ZjPHGPX2 activity, contributing to reactive oxygen species (ROS) detoxification and improved disease resistance (Zhang et al. 2024). These findings indicate that Kcr is a key regulatory mechanism in plant stress responses and immunity.

Wheat leaf rust, caused by *Puccinia triticina*, leads to significant yield losses or even complete crop failure during epidemic years (Bolton et al. 2008; Huerta‐Espino et al. 2011; McCallum et al. 2024). Elucidating the molecular mechanisms and uncovering the immune processes involved in wheat resistance to *P. triticina* infection are crucial for ensuring global food security and sustainable development. Previous studies have revealed that the accumulation of ROS plays a crucial role in wheat resistance to *P. triticina* infection, with ROS levels varying significantly between compatible and incompatible interactions (Wang et al. 2020; Qiao et al. 2015). ROS are one of the earliest molecules produced during the plant immune response to pathogen invasion (Torres et al. 2006; Vellosillo et al. 2010). The rapid production and transient accumulation of ROS, defined by oxidative bursts of hydrogen peroxide (H_2_O_2_), superoxide anions (O_2_^−^), hydroxyl radicals (•OH), and singlet oxygen (^1^O_2_), plays a central role in plant defence signalling (Mehdy 1994; Rui et al. 2024). Among these, H_2_O_2_, as the most stable ROS, has attracted much attention in ROS‐related studies and serves as a key indicator of plant immune system activation (Kuźniak and Urbanek 2000). Initially, the excessive ROS levels induced by pathogen invasion were thought to cause oxidative damage to plant proteins, nucleic acids and lipids, functioning as toxic by‐products of aerobic metabolism. However, ROS are now recognised as essential signalling molecules that activate proteins and metabolic pathways involved in pathogen response, restrict further pathogen spread and ultimately confer systemic resistance in plants (Kuzniak and Kopczewski 2020; Wang et al. 2024; Myers et al. 2024). Our previous research also demonstrated that, in the incompatible combination of wheat and *P. triticina*, early accumulation of ROS acts as a defence signal, while the increase of ROS levels at later stages is extremely important for the cell death and the killing of *P. triticina* through the hypersensitive response (HR) (Sun et al. 2024; Qiao et al. 2015).

Plants regulate ROS homeostasis through diverse enzymatic and non‐enzymatic mechanisms (He et al. 2017). The enzymatic mechanisms primarily involve peroxidases (PRXs), catalases (CATs), superoxide dismutases (SODs) and ascorbate peroxidases (APXs) (Wani et al. 2021). Among these, PRXs constitute a large gene family that regulates the metabolism of ROS and reactive nitrogen species (RNS), directly or indirectly affecting plant defence responses (Terron‐Camero et al. 2022; Kapoor et al. 2019). In plants, the activities of antioxidant enzymes can be regulated by PTMs, thereby influencing the plant's response to stress (Sharova and Medvedev 2024; Marti‐Guillen et al. 2022). In *Arabidopsis thaliana* , the Cys32 residue of APX undergoes S‐nitrosylation and S‐sulfhydration, both of which enhance its H_2_O_2_ scavenging activity and improve plant tolerance to oxidative stress (Aroca et al. 2015; Yang et al. 2015). Mn‐SOD (MSD2) undergoes nitration at the Tyr68 residue, which is located near the enzyme's active site and may hinder substrate accessibility, thereby reducing its activity (Chen et al. 2022). Similarly, S‐nitrosylation of the cysteine residue in AtCAT inactivates its enzymatic activity (Corpas et al. 2019). The Kcr modification of ZjPHGPX2 positively regulates its enzymatic activity, thereby enhancing the antioxidant capacity of jujube trees and increasing their resistance to phytoplasma stress (Zhang et al. 2024). Therefore, the catalytic activity of antioxidant enzymes modulated by PTMs is considered particularly important for plant tolerance to environmental stresses.

In recent years, although some progress has been made in understanding the role of Kcr in plant responses to abiotic stress (Zhu et al. 2023; Zhang et al. 2023; Yang et al. 2022), its roles in biotic stress remain poorly understood. In this study, trichostatin A (TSA) treatment was employed to manipulate Kcr levels in wheat, and the susceptibility of plants with altered Kcr abundance to *P. triticina* infection was subsequently assessed. Subsequent global crotonylome analysis of wheat resistance to *P. triticina* revealed that Kcr may affect wheat susceptibility to *P. triticina* by regulating antioxidant enzyme activity. A key peroxidase, TaPRXIIB, whose Kcr level was significantly increased during *P. triticina* infection, was found to negatively regulate wheat resistance. We further demonstrated that the crotonylation at K172 of TaPRXIIB positively regulates wheat resistance to *P. triticina* by suppressing its activity, thereby promoting ROS accumulation in wheat during the early stage of *P. triticina* infection. Finally, we proposed a TaPRXIIB‐Kcr‐ROS model, which may regulate wheat susceptibility to *P. triticina* infection by promoting ROS accumulation.

## 2 Results

### 2.1 Lysine Crotonylation in Wheat During *P. triticina* Infection

To investigate lysine crotonylation (Kcr) in wheat during *P. triticina* infection, we conducted western blotting with a pan anti‐crotonyllysine antibody. In the incompatible combination, Kcr was found to be widely distributed at multiple time points following *P. triticina* infection (Figure S1a,b). As a histone deacetylase inhibitor, trichostatin A (TSA) has been extensively used to induce epigenetic modifications in human cell lines and model organisms (Azechi et al. 2013). Wheat and *P. triticina* were treated with 15 or 30 μM TSA. The results showed no visible phenotypic changes in wheat leaves at either TSA concentration compared with the control (MOCK) (Figure S1c). To evaluate whether TSA has an impact on the germination rate of *P. triticina*, the fresh urediniospores were treated with different concentrations of TSA. The results showed that 15 μM TSA had no significant effect on tube length, spore germination rate, or tube bifurcation rate. However, under the 30 μM TSA treatment, the bifurcation rate of tubes was significantly higher than that of the control, indicating that the 30 μM TSA treatment affected the growth and development of *P. triticina* (Figure S1d–g). Therefore, 15 μM TSA was selected for subsequent experiments.

**FIGURE 1:**
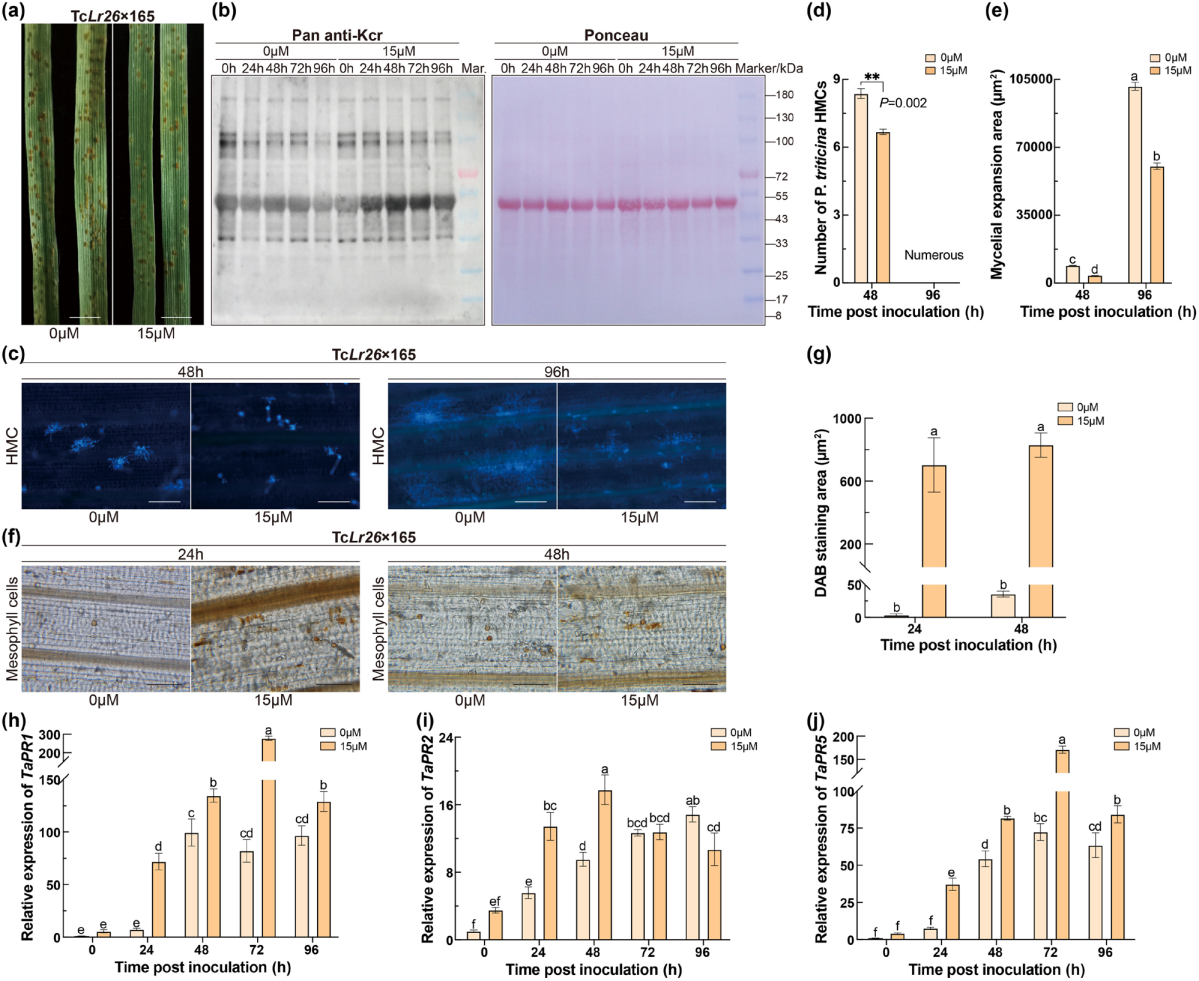
Trichostatin A (TSA) treatment enhances wheat resistance to *Puccinia triticina* infection. (a) Phenotype of the Tc*Lr26* wheat leaves at 7 days post‐inoculation (dpi) with *P. triticina* race 165 under TSA treatment. Scale bar = 0.5 cm. (b) Immunoblot analysis of the total lysine crotonylation (Kcr) proteins at various time points after *P. triticina* infection under TSA treatment. (c) Effects of TSA treatment on wheat resistance to *P. triticina* infection. Scale bar = 250 μm. (d, e) Quantification of haustorial mother cells (HMCs) and *P. triticina* mycelial area. Statistical significance is indicated by asterisks: **p < 0.01. (f) 3,3′‐diaminobenzidine (DAB) staining of H_2_O_2_ during *P. triticina* infection under TSA treatment. Scale bar = 150 μm. (g) Quantification of DAB‐stained regions. (h–j) Relative expression of *TaPR1*, *TaPR2* and *TaPR5* in Tc*Lr26* leaves infected with *P. triticina* race 165 under TSA treatment. All values were from 50 single infection sites, each from three independent biological repeats. Different lowercase letters indicate significant differences (*p* < 0.05), as determined by one‐way ANOVA followed by Duncan's multiple range test.

In the compatible combination consisting of *P. triticina* race 165 and Tc*Lr26*, the number of *P. triticina* uredosori on the leaves under TSA treatment was fewer compared to the control (Figure 1a). Using a pan anti‐crotonyllysine antibody, we performed western blotting and found that the abundance of Kcr was consistently higher than in the control during *P. triticina* infection (Figure 1b). Compared to the control, TSA‐treated Tc*Lr26* plants exhibited fewer *P. triticina* haustorial mother cells (HMCs) and a smaller mycelial expansion area at 48 and 96 h post‐inoculation (hpi) (Figure 1c–e). H_2_O_2_ accumulation was visualised by 3,3′‐diaminobenzidine (DAB) staining at 24 and 48 hpi (Figure 1f). The results demonstrated that Tc*Lr26* plants exhibited a statistically significant increase in the area of DAB staining under TSA treatment compared to the control (Figure 1g). In addition, the expression levels of wheat pathogenesis‐related genes (*TaPR1*, *TaPR2* and *TaPR5*) were significantly higher under TSA treatment than in the control at different time points after *P. triticina* infection (Figure 1h–j), although the expression of *TaPR2* declined at 96 hpi. These results are consistent with the hypothesis that elevated protein crotonylation levels enhance wheat resistance to *P. triticina*.

### 2.2 Proteome‐Wide Identification and Characteristics of Kcr Sites and Proteins Involved in Wheat Resistance to *P. triticina*

**FIGURE 2:**
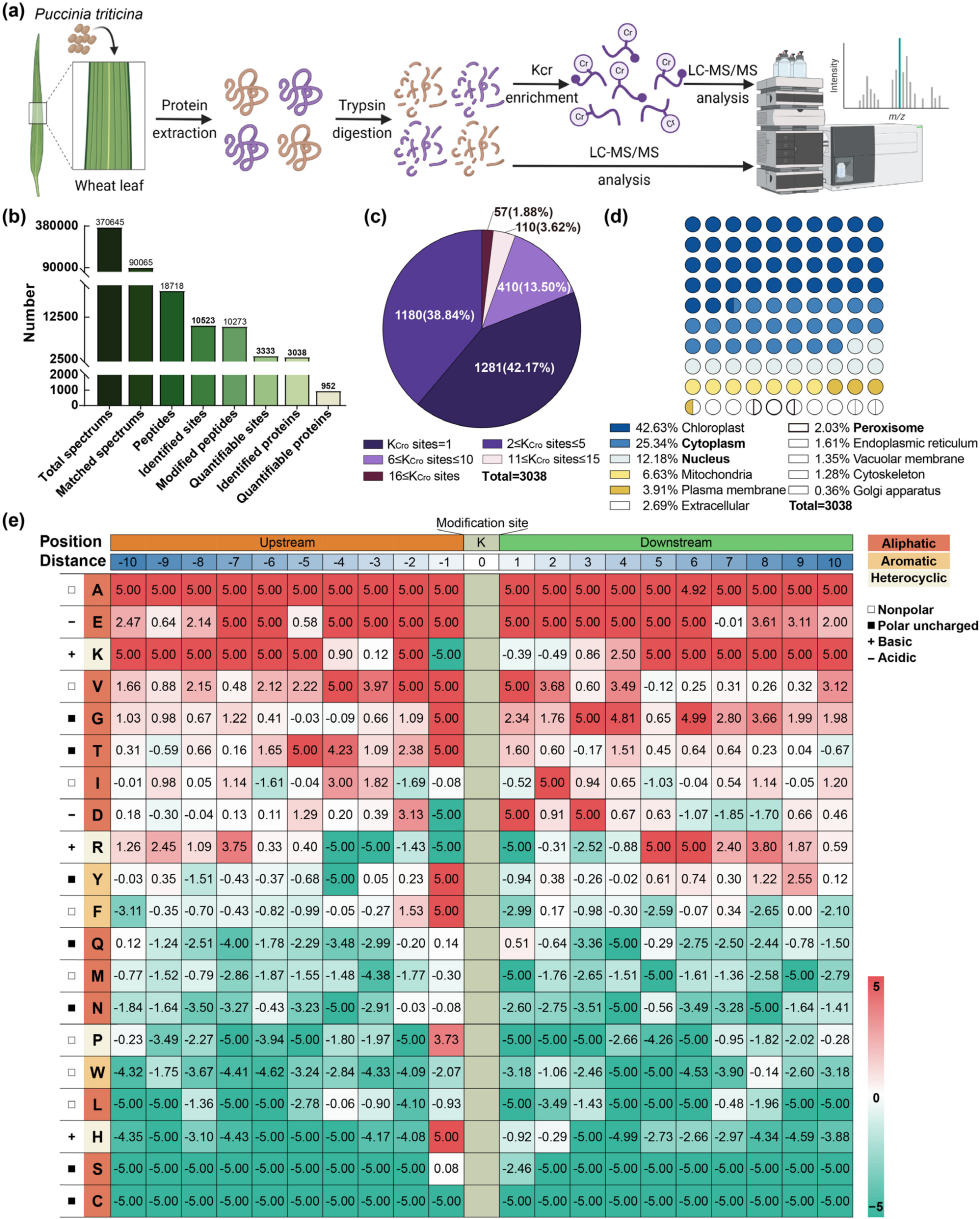
Proteome‐wide identification and characterisation of lysine crotonylation (Kcr) sites and proteins in Tc*Lr26* wheat leaves infected by *Puccinia triticina* race 260. (a) Experimental flowchart for identifying proteome‐wide Kcr sites and proteins by LC–MS/MS. (b) Overview of Kcr proteomics identified through MS/MS spectral analysis. (c) The quantity and proportion of proteins with different Kcr modification sites. (d) Subcellular localisation of Kcr proteins. (e) Frequency distribution of different amino acid residues around Kcr sites. Red indicates enrichment and green indicates depletion.

To comprehensively identify and characterise Kcr sites and proteins involved in wheat resistance to *P. triticina*, we conducted a proteome‐wide analysis of lysine crotonylation in Tc*Lr26* wheat leaves infected with *P. triticina* race 260 at 0 and 24 hpi using 4D label‐free quantitative (4D‐LFQ) LC–MS/MS (Figure 2a). In total, we identified 10,523 distinct Kcr sites in 3038 proteins, with 3333 quantifiable sites on 952 quantifiable proteins (Figure 2b). Among the identified proteins, 10,367 distinct Kcr sites were detected on 2976 non‐histone proteins, with 3326 quantifiable sites on 948 quantifiable proteins (Table S1). The Kcr peptide length ranged from 7 to 37 amino acids and 93.6% of them showed 7–20 amino acids (Figure S2a). All the mass errors were less than 10 ppm, confirming the high accuracy of the MS data (Figure S2b). Principal component analysis (PCA) showed distinct group separation, supporting the reliability of the proteomics data and the richness of the dataset (Figure S2c). Of the 3038 Kcr proteins, more than 1757 (57.8%) wheat proteins were found to have multiple Kcr sites (Figure 2c). The average Kcr level reached 3.46, surpassing previously reported levels (Table S2). In the subcellular localisation analysis, the crotonylated proteins were mainly distributed in the chloroplasts (42.6%), cytoplasm (25.3%) and nucleus (12.2%) (Figure 2d). Notably, 2.0% of the modified proteins were located in the peroxisome (Figure 2d). These results reveal a comprehensive overview of the crotonylation events in wheat during the early stages of *P. triticina* infection, suggesting that Kcr could play essential roles in regulating modified proteins involved in wheat–*P. triticina* interactions.

To analyse sequence patterns near Kcr sites, the amino acid distribution from −10 to +10 positions around the identified Kcr sites were analysed using an iceLogo‐based algorithm (Figure 2e). Sixteen highly conserved motifs with motif scores greater than 20 were identified (Figure S2d). As shown in Figure 2e, aliphatic A residues were frequently observed from positions −10 to +10; enrichment of aliphatic E residues was detected at positions −7 to −6 and −4 to +6, while positively charged K residues were markedly enriched at −10 to −5, −2 and +5 to +10. In wheat, aliphatic residues V, G and T, aromatic residues Y and F, and the heterocyclic residue H were enriched at the −1 position, whereas aliphatic residues V and D were enriched at the +1 (Figure 2e). These results suggest a preference for aliphatic amino acids surrounding Kcr sites. Protein secondary structures of all the identified proteins indicated that 27.1%, 6.4% and 66.5% of the Kcr sites were located in α‐helices, β‐strands and coils, respectively (Figure S2e). Compared with unmodified lysine residues, Kcr sites showed a significant preference for β‐strand (*p* = 6.8e−6) and coil (*p* = 1.7e−5) regions (Figure S2e). Therefore, it is demonstrated that Kcr has a preference for the secondary structure of proteins. The surface accessibility of modified lysine residues was significantly lower than that of unmodified (Figure S2f), suggesting that Kcr sites are preferentially located within protein structures.

### 2.3 Functional Enrichment of Crotonylated Differentially Expressed Proteins in Wheat Resistance to *P. triticina*

**FIGURE 3:**
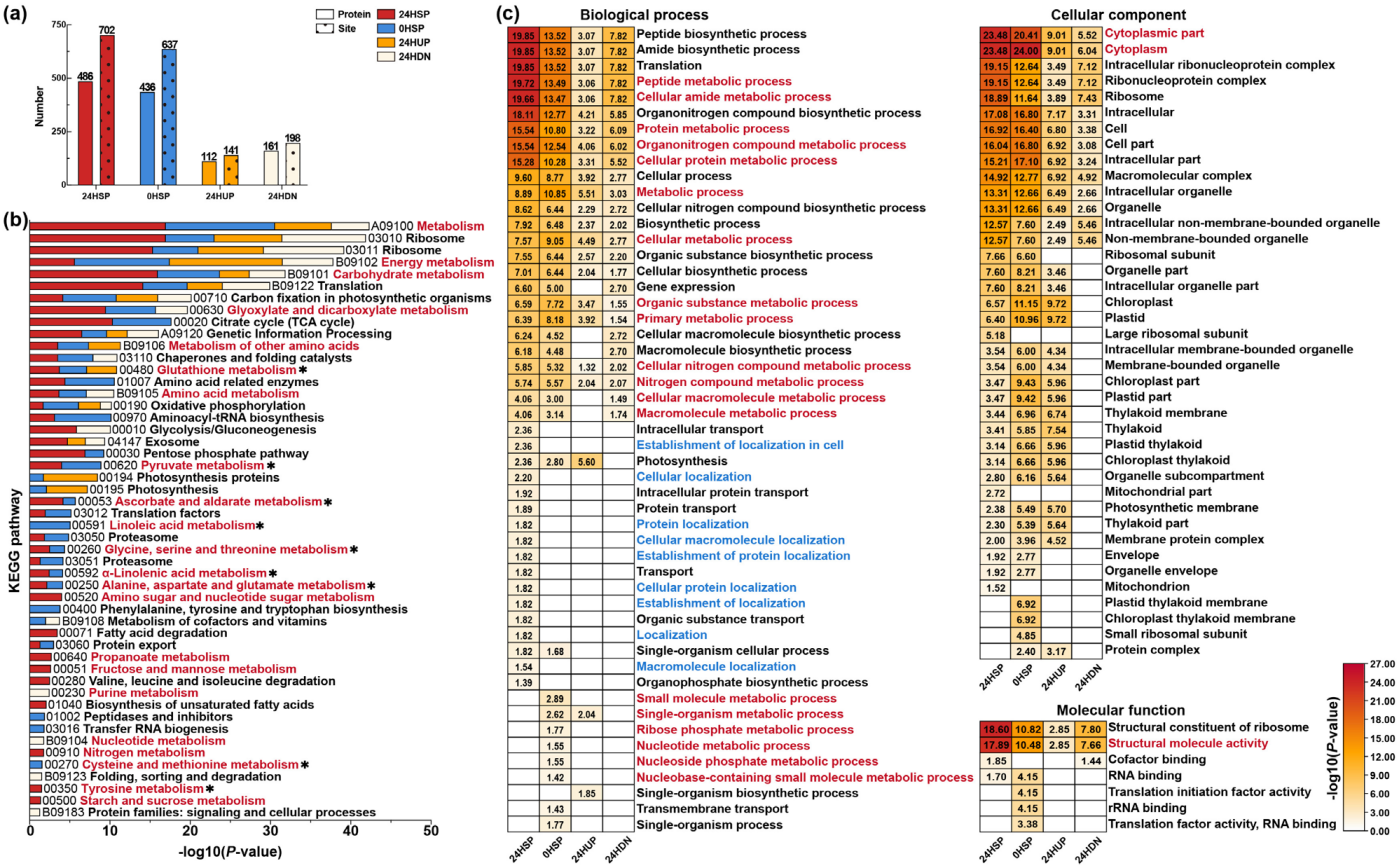
Functional enrichment analysis of significantly differentially modified proteins. (a) Categorisation of differential modification and proteins. 24HSP: Values were available only at 24 h post‐inoculation (hpi) among the time points of 0 and 24 hpi; 0HSP: Values were available only at 0 hpi among the time points of 0 and 24 hpi; 24HUP: 24 hpi modification is upregulated, with fold‐change (FC) > 1.5 compared to 0 hpi; 24HDN: 24 hpi modification is downregulated, with FC > 1.5 compared to 0 hpi. (*t*‐test: *p* < 0.05). (b) KEGG pathway‐based enrichment analysis of crotonylated proteins. Metabolism‐related items are shown in red font and reactive oxygen species (ROS) metabolism‐related items are labelled with an asterisk. (c) GO‐based enrichment analysis of crotonylated proteins in biological process, cellular component and molecular function. Items related to metabolic processes, cytoplasmic or structural activities are shown in red font, and those related to localisation are shown in blue font.

To explore the roles of Kcr in wheat resistance to *P. triticina*, crotonylated differentially expressed proteins (DEPs) were identified and categorised into four groups: 24HSP, 0HSP, 24HUP and 24HDN (Figure 3a and Table S3). A total of 702 sites in 486 crotonylated proteins were identified in 24HSP, 637 sites in 436 proteins in 0HSP, 112 sites in 141 proteins in 24HUP and 198 sites in 161 proteins in 24HDN (Figure 3a and Table S3). Statistical analysis showed that at 24 hpi, proteins with upregulated modifications in 24HSP and 24HUP accounted for 50.0% of total proteins, while upregulated modification sites comprised 50.2% of total sites.

To clarify the functions of these crotonylated DEPs in wheat, KEGG pathway enrichment analysis and GO (Gene Ontology) enrichment analysis were performed (Figure 3b,c). The KEGG pathway enrichment analysis showed that the upregulated modification proteins (24HSP and 24HUP) at 24 hpi were predominantly enriched in pathways related to metabolism, ribosome, energy metabolism, carbohydrate metabolism, translation, amino acid metabolism, glutathione metabolism and oxidative phosphorylation; the downregulated modification proteins (0HSP and 24HDN) exhibited no specific pathways enrichment (Figure 3b). The GO enrichment analysis included biological processes, cellular components and molecular functions (Figure 3c). In terms of biological processes, both upregulated and downregulated Kcr proteins were enriched in pathways similar to those identified in KEGG analysis. Notably, all four groups exhibited a high degree of enrichment in metabolism‐related processes. In the cellular component category, the upregulated and downregulated modification proteins were enriched mainly in the cytoplasm and ribosome. In the molecular function category, Kcr‐modified proteins associated with structural components (ribosomes) or activity were enriched. These results suggest that Kcr proteins are involved in several critical processes, particularly cytoplasmic substance metabolism and molecular activity. Notably, the upregulated modification proteins (24HSP and 24HUP) contributed to some ROS scavenging‐related pathways, such as glutathione metabolism and ascorbate and aldarate metabolism.

### 2.4 Alterations in the Crotonylation of Antioxidant Enzymes Are Involved in Wheat Adaptation to Stress During *P. triticina* Infection

**FIGURE 4:**
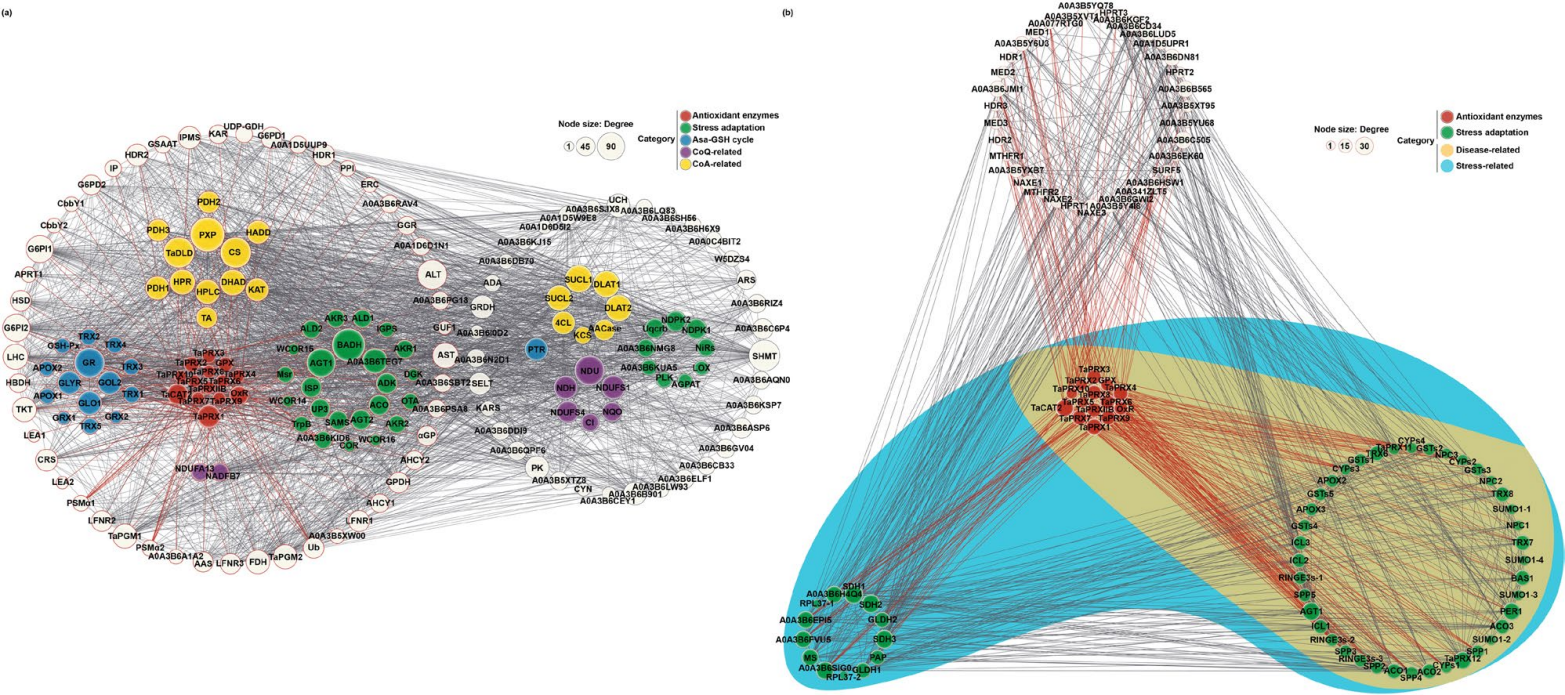
Interactions between differentially crotonylated antioxidant enzymes and associated proteins in wheat during *Puccinia triticina* infection. (a) Crotonylated proteins interaction network in the incompatible combination of Tc*Lr26* and *P. triticina* race 260. (b) Interaction network of differentially modified antioxidant enzymes and associated proteins. Proteins sharing the same functional category are shown in circles of the same colour. Circle size reflects interaction degree. Red and black lines represent first‐ and second‐degree neighbours, respectively.

Growing evidence indicates that PTMs play a critical role in cellular signalling by mediating protein interactions and coordinating regulatory processes, thereby forming complex networks that govern cellular functions (Sharma et al. 2023; Lee et al. 2023; Agbemafle et al. 2023; Pandey and Gayen 2024). To gain deeper insights into the role of ROS‐scavenging‐related proteins in wheat resistance to *P. triticina*, we constructed a protein–protein interaction (PPI) network using 1195 differentially modified proteins from 24HSP, 0HSP, 24HUP and 24HDN groups, each with a confidence score above 0.7. The analysis revealed that the network includes 173 crotonylated DEPs as nodes, connected by 5006 physical interactions (Figure 4a and Table S4). Node proteins can be classified into five functional groups: (1) 14 antioxidant enzymes (red dots in Figure 4a) involved in maintaining intracellular ROS balance and playing key roles in plant disease resistance; (2) 33 stress adaptation proteins (green dots) participating in plant defence responses and cellular adaptation to environmental stress; (3) 15 AsA‐GSH cycle proteins (blue dots) associated with cellular redox homeostasis and antioxidant metabolic pathways; (4) 8 CoQ‐related proteins (purple dots) functioning as non‐enzymatic antioxidants, primarily involved in intracellular energy metabolism and antioxidant defence; and (5) 19 CoA‐related proteins (yellow dots), essential for fatty acid and pyruvate metabolism and serving as donor molecules in protein crotonylation. Among the observed interactions in the PPI network, antioxidant enzymes were physically connected to 52 stress‐related proteins through 84 interactions, which accounted for 51.5% of the total 101 direct interaction partners, highlighting a strong association between Kcr‐modified antioxidant enzymes and stress response processes. Furthermore, using the STRING database, we constructed a PPI network between antioxidant enzymes and the wheat proteome (Figure 4b and Table S5), including 102 protein nodes with 1610 direct physical interactions. Among these proteins, 53 are stress‐related, of which 40 are involved in disease‐related processes, accounting for 75.5% (Figure 4b and Table S5). These findings suggest that crotonylated DEPs, especially antioxidant enzymes, play key roles in wheat defence against environmental stresses and pathogenic challenges.

Among the crotonylated DEPs, 12 were selected for validation of crotonylation sites using parallel reaction monitoring (PRM) analysis (Figure S3a). PRM analysis at 24 hpi revealed significantly increased Kcr levels in eight proteins: ADP‐ribosylation factor (TaARF^K36^, Q76ME3^K36^), phospholipase D (TaPLD^K262^, W5D4Q6^K262^), histone H2A (TaH2A^K128^, A0A1D5YKH2^K128^), large ribosomal subunit protein (TaRSL2D^K198^, W5ECL2^K198^), large ribosomal subunit protein (TaRPL22^K101^, Q95H48^K101^), 40S ribosomal protein S7 (TaRPS7^K91^, Q5I7K2^K91^), peroxiredoxin (TaPRXIIB^K172^, C6ETA5^K172^) and catalase (TaCAT2^K426^, F1DKC1^K426^). Conversely, five proteins exhibited decreased Kcr levels: phosphoglycerate mutase (2,3‐diphosphoglycerate‐independent) (TaPGM^K243^, W5D322^K243^), flavone O‐methyltransferase (TaOMT^K186^, Q84N28^K186^), prefoldin subunit β (TaPFDNB^K84^, W5BRT4^K84^), dihydrolipoyl dehydrogenase (TaDLD^K404^, W5A874^K404^) and catalase (TaCAT2^K125^, F1DKC1^K125^). Among them, only four proteins showed significant changes in abundance between 0 and 24 hpi, all of which were upregulated at 24 hpi, including TaPRXIIB, TaCAT2, TaPLD and TaOMT (Figure S3b). Reverse transcription‐quantitative PCR (RT‐qPCR) analysis was conducted to examine the expression of the 12 corresponding genes at different time points following *P. triticina* infection (Figure S3c). Remarkably, *TaPRXIIB* and *TaCAT2*, the two antioxidant enzyme genes, were both induced during *P. triticina* infection, and exhibited higher expression levels in the compatible combination (*P. triticina* race 165 and Tc*Lr26*) than in the incompatible combination (*P. triticina* race 260 and Tc*Lr26*) (Figure S3c). Based on the PPI network, PRM validation and gene expression patterns, subsequent analyses focused on TaPRXIIB and TaCAT2.

### 2.5 Sequence Features and Kcr Site Analysis of TaPRXIIB and TaCAT2

**FIGURE 5:**
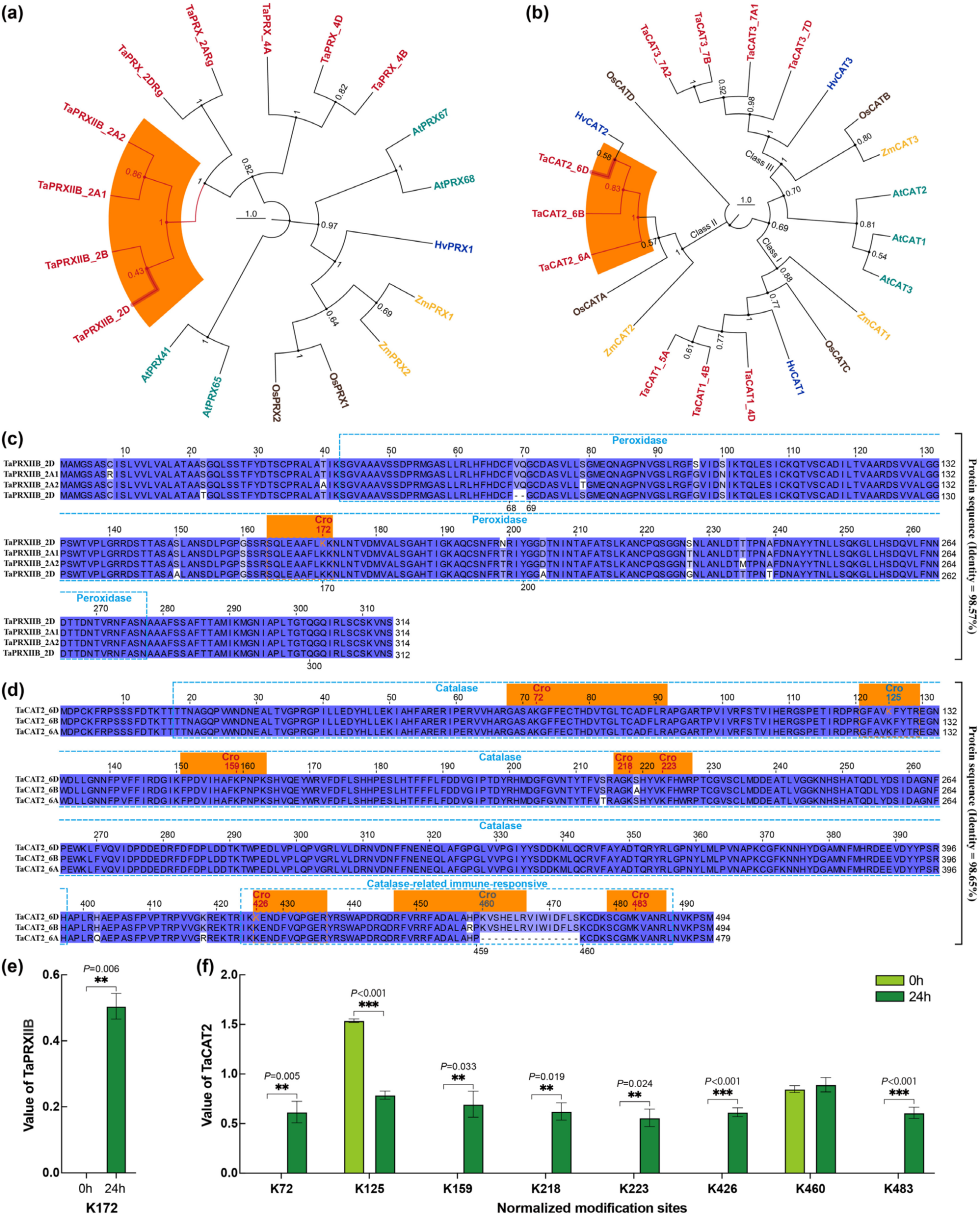
Crotonylation sites analysis of TaPRXIIB and TaCAT2. (a, b) Phylogenetic trees of TaPRXIIB and TaCAT2, including three related species with *Arabidopsis* as an outgroup. Copies of TaPRXIIB and TaCAT2 are highlighted with an orange background. Numbers represent bootstrap values. Ta: wheat; Hv: barley; Os: rice; Zm: maize; At: *Arabidopsis*. (c) and (d) Comparison of different copies of TaPRXIIB and TaCAT2. Identified modified peptides are highlighted with an orange background. Red denotes upregulation of modifications at 24 h, blue shows downregulation and grey indicates no change. Blue dashed lines outline conserved functional regions in functional proteins. (e, f) Normalised statistics of modification sites for TaPRXIIB and TaCAT2. Highly significant differences and extremely significant differences are shown as **p < 0.01 and ***p < 0.001, respectively.

To investigate the functions of crotonylated TaPRXIIB and TaCAT2 in wheat resistance to *P. triticina*, we firstly constructed phylogenetic trees of TaPRXs and TaCATs across wheat, barley, rice, maize and *Arabidopsis* (Figures 5a,b and S4a,b). Phylogenetic analysis revealed that TaPRXIIB and TaCAT2 possess four and three homologous copies, respectively, distributed across the A, B and D subgenomes of wheat, with each sharing more than 98% sequence identity. In addition, TaPRXIIB was homologous to AtPRX41/65/67/68, OsPRX1/2, ZmPRX1/2 and HvPRX1, while TaCAT2 was most closely related to HvCAT2 (Figure 5a,b). Furthermore, at 24 hpi, only one Kcr site (K172) in TaPRXIIB exhibited a higher crotonylated level compared to 0 hpi. This site is located within the peroxidase domain and is conserved across the four copies and major homologous genes in wheat (Figures 5c and S4a). However, TaCAT2 showed significant changes in Kcr levels at seven sites at 24 hpi, including a significant decrease at K125 and significant increases at K72, K159, K218, K223, K426 and 483. These sites are located within catalase‐related domains and are highly conserved across different species (Figure 5d and S4b). In addition, the Kcr level of the K426 site in TaCAT2 did not change between 0 and 24 hpi and this site was not conserved across different species (Figures 5d and S4b). Given the complex modification patterns observed at eight Kcr sites of TaCAT2, we selected TaPRXIIB for further study to better elucidate the potential role of Kcr modification in wheat disease resistance.

### 2.6 Downregulation of *TaPRXIIB* Enhances Wheat Resistance by Promoting H_2_O_2_ Accumulation

**FIGURE 6:**
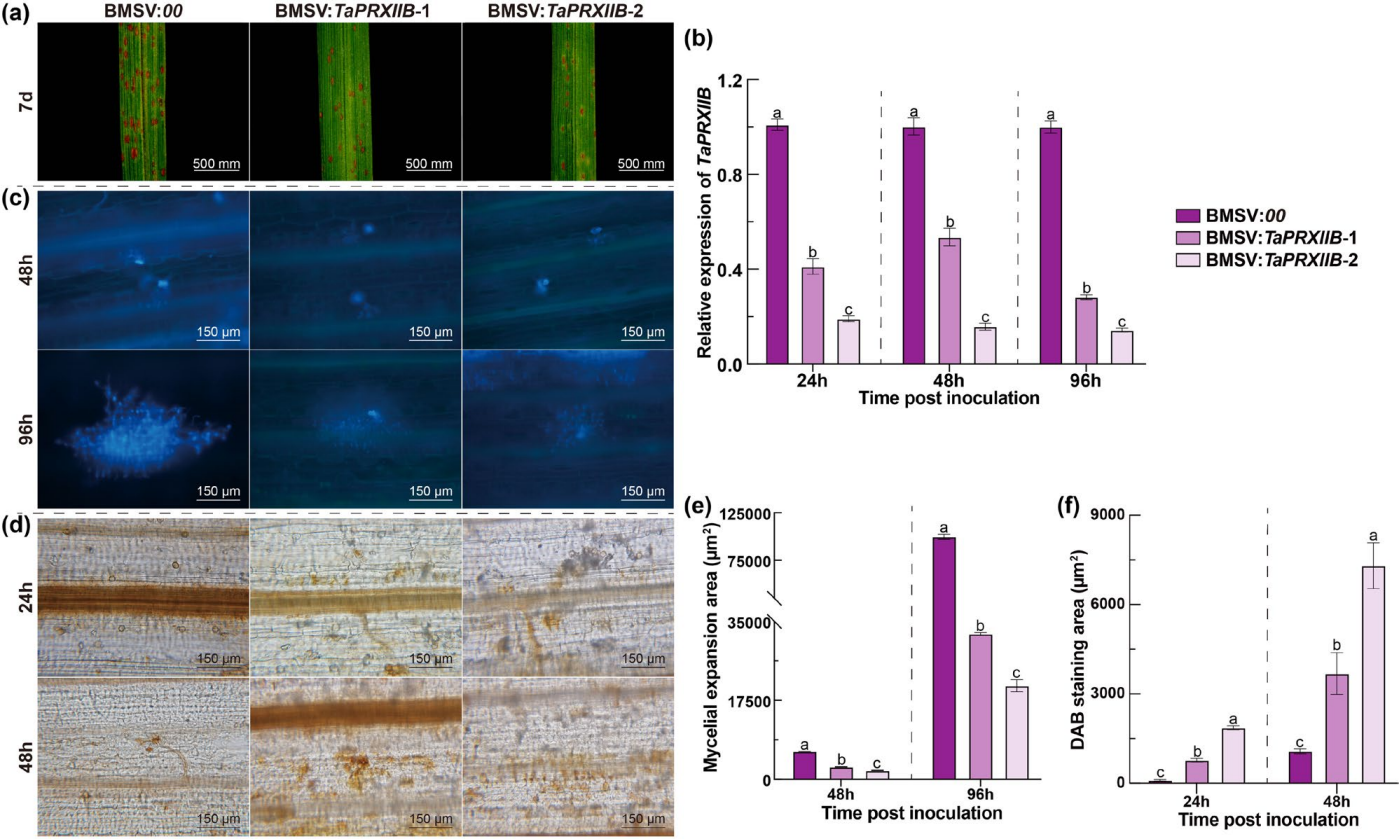
Virus‐induced gene silencing (VIGS) of *TaPRXIIB* reduces wheat susceptibility to *Puccinia triticina* infection. (a) Phenotypic analysis of *TaPRXIIB*‐silenced plants at 7 days post‐infection with *P. triticina*. (b) Silencing efficiency of *TaPRXIIB* in VIGS experiments. Values are from three independent replicates. (c, e) Mycelial expansion and quantification of mycelial expansion area in *TaPRXIIB*‐silenced leaves at 48 and 96 h post‐inoculation (hpi) with *P. triticina*. (d, f) 3,3′‐diaminobenzidine (DAB) staining visualising H_2_O_2_ accumulation and its quantified area in *TaPRXIIB*‐silenced leaves at 24 and 48 hpi with *P. triticina*. All values are from 50 single infection sites, each from three independent biological repeats. Lowercase letters indicate significant differences (*p* < 0.05) determined by one‐way ANOVA and Duncan's multiple range test.

To elucidate the role of TaPRXIIB in wheat resistance to *P. triticina* infection, we used barley stripe mosaic virus (BMSV)‐induced gene silencing (VIGS) to suppress its expression in the compatible combination (Figure 6 and Table S8). Wheat plants inoculated with BSMV:*PDS* showed typical photobleaching symptoms, while those inoculated with BSMV:*TaPRXIIB* exhibited typical symptoms of BSMV infection (Figure S5a). To assess the silencing efficiency of *TaPRXIIB*, wheat plants inoculated with BSMV:*TaPRXIIB* were challenged with *P. triticina* race 165, and *TaPRXIIB* expression levels were measured at 24, 48 and 72 hpi (Figure 6b and Table S8). Compared to the control plants (BSMV:00), *TaPRXIIB* expression was significantly reduced in BSMV:*TaPRXIIB* plants (Figure 6b), whereas the expression of *TaPRXIIB_Rg*, the closest homologue, remained unchanged, indicating that the silencing was both efficient and specific (Figure S5b,c). At 7 days post‐inoculation (dpi), the number of urediniospores on leaves of *TaPRXIIB*‐silenced wheat plants (BSMV:*TaPRXIIB*) was significantly reduced compared to the control group (BSMV:00) (Figure 6a). Rohringer staining revealed a noticeable increase in the mycelial expansion area, which negatively correlated with the silencing efficiency of *TaPRXIIB* (Figure 6c,e), indicating that reduced *TaPRXIIB* expression enhances wheat resistance to *P. triticina*. DAB staining results showed that the *TaPRXIIB*‐silenced plants exhibited significantly higher H_2_O_2_ levels at 24 and 48 hpi compared to the control plants (Figure 6d,f). These findings indicate that the reduced expression of *TaPRXIIB* enhances wheat resistance to *P. triticina* infection through modulating ROS homeostasis.

### 2.7 Crotonylation at Site K172 Negatively Regulates TaPRXIIB Activity

**FIGURE 7:**
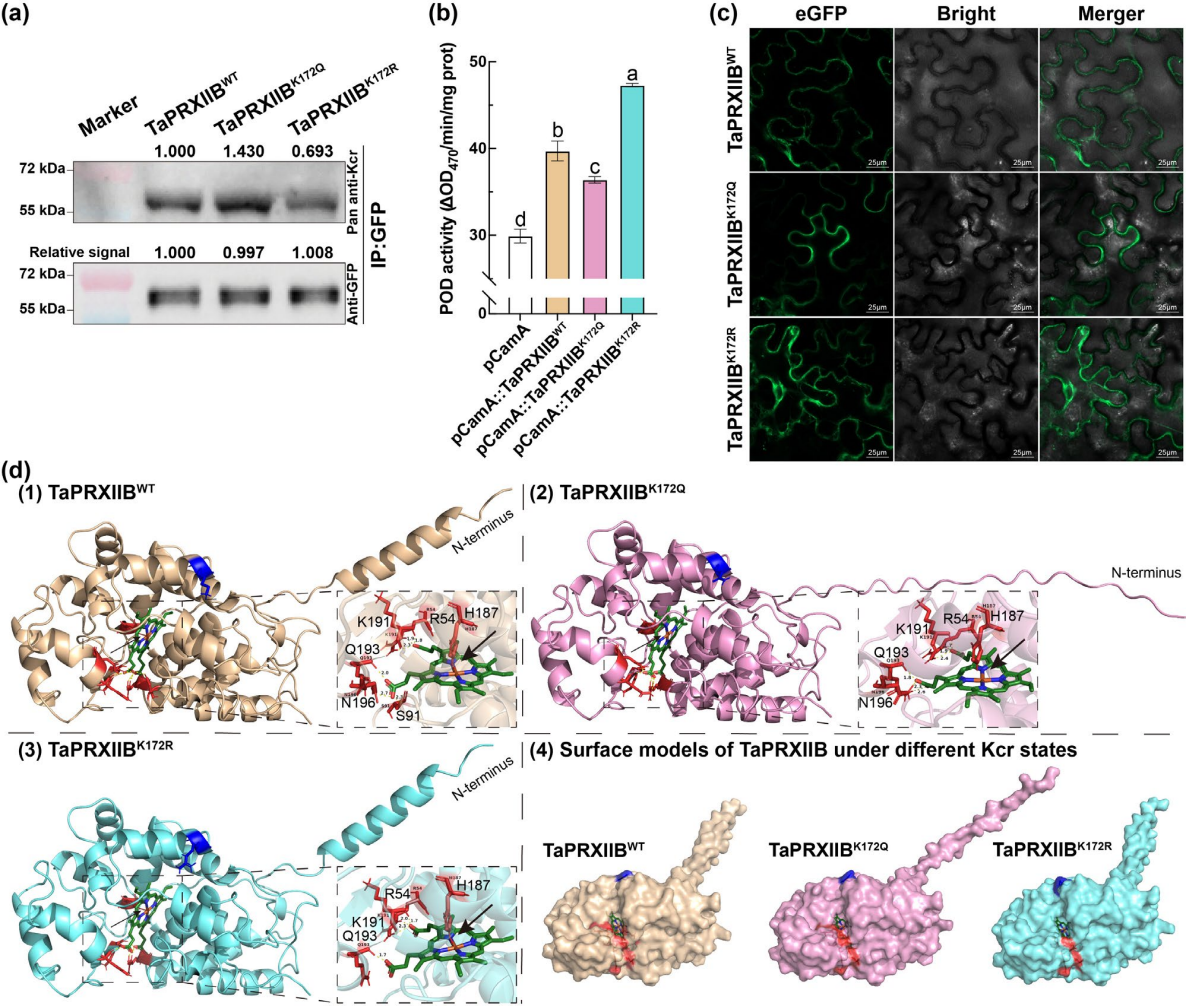
Functional analysis of TaPRXIIB under different lysine crotonylation (Kcr) states. (a) Validation of the effectiveness of the crotonylation site at lysine 172 in TaPRXIIB. WT, wild type (K172). (b) TaPRXIIB activity in eukaryotic system. Values are from three independent biological replicates. Lowercase letters indicate significant differences (*p* < 0.05) determined by one‐way ANOVA and Duncan's multiple range test. (c) Subcellular localisation of TaPRXIIB under different modification states. (d) 3D structure of TaPRXIIB in complex with its prosthetic group. In the protein structure, red highlights represent amino acid residues forming direct hydrogen bonds with the prosthetic group, while blue highlights denote different modification states of lysine at position 172. Hydrogen bonds are indicated by yellow dashed lines, with the numbers representing the distances (Å). The dashed box highlights the detailed interactions between the apoprotein and its prosthetic group. K172Q represents a crotonyl‐mimetic mutant, while K172R represents a crotonyl‐dead mutant.

To investigate the role of TaPRXIIB^K172^ crotonylation in wheat resistance to *P. triticina*, lysine (K) 172 was mutated to glutamine (Q) and arginine (R) to generate crotonyl‐mimetic and crotonyl‐dead mutants, respectively. Subsequently, we transiently overexpressed TaPRXIIB and its two mutants (TaPRXIIB^K172Q^ and TaPRXIIB^K172R^) in *Nicotiana benthamiana*. Western blotting analysis revealed that Kcr levels were markedly elevated in TaPRXIIB^K172Q^ and reduced in TaPRXIIB^K172R^ relative to the wild‐type protein (TaPRXIIB^WT^) (Figure 7a and S6). The enzyme activity assay showed that the POD activity of TaPRXIIB^K172Q^ was significantly decreased, whereas that of TaPRXIIB^K172R^ was significantly increased compared to TaPRXIIB^WT^ (Figure 7b). These results indicate that crotonylation at K172 suppresses the enzymatic activity of TaPRXIIB. In addition, subcellular localisation results showed that the TaPRXIIB^WT^, TaPRXIIB^K172Q^ and TaPRXIIB^K172R^ were all localised in the cytoplasm (Figure 7c), indicating that the K172 mutants and the Kcr levels do not affect its subcellular localisation. We performed computational modelling of the three‐dimensional structures of TaPRXIIB^WT^, TaPRXIIB^K172Q^ and TaPRXIIB^K172R^. Cartoon representations showed that both TaPRXIIB^WT^ and TaPRXIIB^K172R^ retain a continuous N‐terminal α‐helix, whereas it is specifically lost in the crotonyl‐mimetic TaPRXIIB^K172Q^ mutant (Figure 7d(1–3)). Surface structure analysis further revealed that apart from the N‐terminal region, no obvious differences were observed in the rigid core structure of the protein across different Kcr states (Figure 7d(4)).

The heme (ferroprotoporphyrin IX), the prosthetic group of peroxidases, is crucial for their catalytic activity (Yu et al. 2020; Stenbaek and Jensen 2010; Marañón and Van Huystee 1994). We performed the molecular docking between TaPRXIIB and heme at different modification states to elucidate how Kcr at 172 influences its enzymatic activity (Figure 7d). Site analysis revealed that H187 forms a hydrogen bond with the heme iron in TaPRXIIB^WT^ and TaPRXIIB^K172R^, whereas this interaction is disrupted in the crotonyl‐mimetic TaPRXIIB^K172Q^. When the absolute value of the binding energy is high and the inhibition constant is low, the binding affinity between the enzyme and its ligand is stronger, which is generally associated with higher enzymatic activity (Du et al. 2016). Therefore, we quantified the binding energy and inhibition constant between TaPRXIIB and heme under different modification states (Table 1). The results showed that the binding energies of TaPRXIIB^K172Q^ and TaPRXIIB^K172R^ were similar to that of TaPRXIIB^WT^ (absolute differences < 1), while the inhibition constants of TaPRXIIB^WT^ and TaPRXIIB^K172R^ were about 190‐fold lower than that of TaPRXIIB^K172Q^ (Table 1). This suggests that the enzymatic activity of TaPRXIIB^WT^ or TaPRXIIB^K172R^ is higher than that of TaPRXIIB^K172Q^, consistent with the enzyme activity assay (Figure 7b). These results suggest that H187 is a key residue for heme binding in TaPRXIIB, and that crotonylation at K172 disrupts the H187–heme hydrogen bond, thereby reducing enzymatic activity and impairing ROS scavenging.

**TABLE 1:** Molecular docking of TaPRXIIB under different modification states with heme.

Protein	Ligand	Binding energy	Inhibitory constant/pM	Number of hydrogen bonds	Amino acid residues forming hydrogen bonds
TaPRXIIB^WT^	Heme	−15.93	2.11	7	R54, K191, Q193, **H187:Fe**, N196, S91
TaPRXIIB^K172Q^	Heme	−16.91	401.61	4	R54, K191, Q193, N196
TaPRXIIB^K172R^	Heme	−15.92	2.14	4	R54, K191, Q193, **H187:Fe**

Abbreviations: Fe, iron ion; H, histidine; K, lysine; N, asparagine; Q, glutamine; R, arginine; S, serine.

## 3 Discussion

Kcr is a novel type PTM and plays crucial roles in regulating a variety of cellular processes in various organisms. Studies, such as those by Zhang et al. (2024), Zhang et al. (2023), Zhu et al. (2023) and Huang et al. (2021) have investigated the relationship between Kcr and plant responses to environmental stress. However, the molecular mechanisms by which Kcr contributes to host resistance against pathogen infection remain poorly understood. In this study, we found that increased Kcr levels are positively correlated with wheat resistance, and for the first time, we report a global crotonylome profiling of wheat following *P. triticina* infection. Further proteomic analyses revealed that crotonylated DEPs were predominantly enriched in metabolic processes and mainly localised in the cytoplasm. Integration with PPI analysis revealed that multiple antioxidant enzymes were affected by Kcr. The modification patterns of two antioxidant enzymes were investigated in detail. We confirmed K172 of TaPRXIIB is the key Kcr site and revealed the negative role of TaPRXIIB in wheat resistance to *P. triticina* through modulating H_2_O_2_ homeostasis. Furthermore, the crotonylation at K172 of TaPRXIIB decreased its enzyme activity through disrupting the hydrogen bond formation between its H187 and the prosthetic group. It should be noted that the K to Q mutation used in this study serves as a biochemical mimic of crotonylation; however, it may not fully recapitulate the structural and functional properties of the native modification. This study provided the global crotonylome profiling in wheat resistance to *P. triticina* and revealed the Kcr modification of TaPRXIIB^K172^ negatively regulates its enzyme activity. Therefore, the results provide valuable resources, a theoretical basis, and new insights for improving disease resistance in wheat breeding.

PTMs serve as crucial regulatory mechanisms that modulate protein functions in eukaryotes (Mann and Jensen 2003). Treatment with TSA has been reported to elevate global lysine acylation levels in wheat proteins, including crotonylation (Bie et al. 2020; Vorontsova et al. 2004). Kcr was first reported by Tan et al. (2011) and was initially identified as predominantly enriched on histones, where it acts as a marker of active gene transcription. Subsequently, Kcr was also found on non‐histone proteins (Sun et al. 2017, 2019). Previous studies on plant stress tolerance have primarily linked Kcr to abiotic stress resistance. For instance, research on cold tolerance in chrysanthemum revealed that decrotonylation of DgGPX1 at K220 enhanced GPX activity, reduced ROS accumulation under low‐temperature stress, and improved cold tolerance (Yang et al. 2022). In wheat, the lysine deacylase TaSRT1 suppresses TaPGK accumulation through decrotonylation, thereby negatively regulating cold tolerance (Zhang et al. 2023). Additionally, Kcr at K367 of TaFBA6 enhances its structural stability in an alkaline environment by reducing the positive charge distribution on the protein surface, thereby promoting its enzymatic activity and improving wheat's salt tolerance (Zhu et al. 2023). However, reports on Kcr in plant responses to biotic stresses are relatively scarce. A recent study by Zhang et al. (2024) demonstrated that elevated Kcr levels at K130/K135 within the unique domain of ZjPHGPX2 enhanced its enzymatic activity, thereby promoting the scavenging of phytoplasma‐induced toxic ROS and strengthening jujube resistance to phytoplasma stress (Zhang et al. 2024). In the present study, we revealed that the increased Kcr level at K172 of TaPRXIIB reduced its enzymatic activity through disrupting the hydrogen bond formation between its H187 and the prosthetic group. This phenomenon, where changes in modification abundance at specific amino acid residues modulate enzymatic activity, has also been frequently reported in studies on other types of PTMs (Xiong and Guan 2012; Narita et al. 2018; Chen et al. 2022; Aroca et al. 2015; Yang et al. 2015; Corpas et al. 2019). Thus, the modification patterns of amino acid residues in proteins facilitate specific intermolecular interactions and, to some extent, modulate the physicochemical properties and functions of proteins. Identifying the crotonylases or decrotonylases responsible for TaPRXIIB^K172^ modification is essential for elucidating the dynamic regulation of Kcr in wheat immunity. Future efforts to systematically identify these enzymes and define their substrate specificity toward TaPRXIIB will provide deeper insight into the regulatory network governing Kcr‐mediated control of ROS homeostasis and disease resistance.

Leaf rust is a critical and ancient wheat disease that affects all wheat growth stages across more than 60 countries worldwide (Hovmoller et al. 2023). Following *P. triticina* infection, some of the earliest inevitable products in wheat are ROS (Wang et al. 2020). In plants, ROS play a dual role in disease resistance: on one hand, they function as signalling molecules involved in pathogen detection and the activation of defence responses; on the other hand, excessive accumulation of ROS can lead to oxidative damage. Antioxidant enzymes are distinguished by their high reaction efficiency, prosthetic group specificity and spatial specificity (Parsonage et al. 2008; Ferrer‐Sueta et al. 2011). Nitric oxide‐mediated post‐translational modifications (NO‐PTMs) regulate the activities of key antioxidant enzymes, especially those involved in the ascorbate‐glutathione cycle (Begara‐Morales et al. 2016). For example, resveratrol enhances glutathione peroxidase (GPX) activity by increasing its phosphorylation, thereby strengthening cellular defence against oxidative stress (Sadi et al. 2014). These findings suggest that PTMs finely regulate the activity of antioxidant enzymes to maintain a dynamic balance between ROS accumulation and scavenging. In this study, we found that there is a strong association between Kcr‐modified antioxidant enzymes and wheat resistance to *P. triticina*. Using VIGS, we found that TaPRXIIB plays a negative role in wheat resistance to *P. triticina*. Furthermore, we revealed that crotonylation at K172 of TaPRXIIB decreases its enzymatic activity (Figure 7b). Further structural analyses based on AlphaFold predictions and molecular docking suggested that this reduction results from altered protein conformation and weakened interaction with the prosthetic group. These indicate that Kcr modification can provide rapid and precise regulation for proteins through influencing conformational space and functions, thereby controlling the infected local regions (Ma et al. 2024). It is worth noting that this study found that the crotonylation at K172 of TaPRXIIB disrupts its hydrogen bond formation between H187 and the prosthetic group and adjusted its enzyme's catalytic pocket, thereby decreasing enzymatic activity without excessively compromising the structural rigidity (Figure 7d). These findings provide valuable insights into balancing the activity and stability trade‐offs in enzyme engineering and broaden the potential applications of PTMs.

In this study, we found that the Kcr site is evolutionarily conserved in wheat and its close relatives (Figure S4). Various conserved motifs preferentially associated with Kcr have been reported in several plants. However, analysis of reported Kcr‐preferred motifs did not identify any universally conserved pattern across species. Even within wheat, distinct Kcr‐associated motifs have been observed under different stimuli that alter Kcr levels (Zhu et al. 2023; Zhang et al. 2023), likely reflecting the recruitment of different functional proteins under specific biological contexts. Moreover, even within the same class of proteins, there are species‐specific differences. These sequence variations are likely a major cause of the differences in Kcr‐preferred motifs. By analysing the amino acid composition surrounding Kcr sites across species and stress conditions, we observed that glutamate (E) and aspartate (D) are consistently enriched in wheat (Zhu et al. 2023; Zhang et al. 2023), rice (Liu et al. 2018), peanut (Xu et al. 2021), tobacco (Sun et al. 2017), chrysanthemum (Lin et al. 2021) and jujube (Zhang et al. 2024). These acidic amino acids may contribute to local structural environments that influence Kcr occurrence and function. Their frequent occurrence suggests that Kcr may influence protein activity, such as enzymatic function. In addition, aromatic residues, including tyrosine (Y) and phenylalanine (F), are also enriched near Kcr sites and may participate in protein–molecule interactions, which could be modulated by nearby crotonylation. Lysine residues have also been frequently found near Kcr sites, which may increase the likelihood of modification in these regions and enhance the stability of Kcr‐mediated protein function regulation. These observations suggest that conserved sequence features surrounding Kcr sites may play important roles in regulating protein structure and function. A systematic understanding of these sequence characteristics could facilitate the identification of broader PTM patterns and support the prediction of novel modification sites. Furthermore, residues that are spatially proximal in the three‐dimensional structure, rather than only those adjacent in the primary sequence, should also be considered when defining PTM‐associated regions.

In conclusion, this study generates a comprehensive dataset of Kcr during *P. triticina* infection in wheat and elucidates the functional role of the Kcr‐modified protein TaPRXIIB in wheat resistance. These findings provide new insights into the regulatory function of Kcr in plant immunity and may guide future strategies for breeding disease‐resistant wheat.

## 4 Experimental Procedures

### 4.1 Materials and Growth Conditions

The Thatcher near‐isogenic wheat line Tc*Lr26* (carrying the *Lr26* gene) was used as the host. *P. triticina* races 260 and 165 establish incompatible and compatible interactions, respectively, on this line. Seedlings were inoculated at the one‐leaf, one‐heart stage with freshly propagated urediniospores. Infected plants were incubated at > 95% humidity for 16 h and then maintained in a growth chamber at 22°C–24°C under a 16 h light/8 h dark cycle.

### 4.2 Pharmacological Study

TSA stock solution (1 mM in dimethyl sulphoxide) was diluted to 0, 15 and 30 μM and injected into wheat leaves, followed by *P. triticina* infection 12 h later. Treatments were applied to nine seedlings with three biological replicates. For spore germination assays, urediniospores were treated with TSA and incubated on agar plates for 24 h under optimal conditions.

### 4.3 Staining and Microscopy

Fluorescence Brightener 28 staining was used to assess mycelial expansion, HMCs and HR areas (Rohringer et al. 1977). H_2_O_2_ accumulation was detected by DAB staining (Daudi et al. 2012). Images were analysed using Fiji. Subcellular localisation was observed by confocal microscopy following transient expression of *eGFP*‐tagged constructs (Wu et al. 2014).

### 4.4 RNA and Protein Analysis

Total RNA was extracted and reverse transcribed. Gene expression was quantified by RT‐qPCR (Table S6) using *TaEF‐1α* as an internal control (Hui et al. 2015) and the 2^−ΔΔ*C*t^ method (Livak and Schmittgen 2001). Protein extraction was performed from tissues ground in lysis buffer, and total protein concentration was measured by BCA assay. Western blotting was conducted using anti‐crotonyllysine and anti‐GFP antibodies. All assays included three biological replicates with triplicate measurements.

### 4.5 Trypsin Digestion, Enrichment and LC–MS/MS

Proteins were lysed, precipitated, resolubilised and digested with trypsin. Peptides were reduced, alkylated and enriched using anti‐crotonyllysine‐conjugated beads. Enriched peptides were desalted and analysed by LC–MS/MS in PASEF mode.

The resulting MS/MS data were processed using the MaxQuant search engine (v. 1.6.15.0). Tandem mass spectra were searched against the *Blast_Triticum_aestivum_4565_PR_20210107.fasta* database (130,673 entries), concatenated with a reverse decoy database and a common contaminants database to estimate the false discovery rate (FDR) and eliminate potential contaminant identifications. Trypsin/P was specified as the cleavage enzyme, allowing up to 4 missed cleavages. The minimum peptide length was set to 7 amino acids, and the maximum number of modifications per peptide was set to 5. The mass tolerance for precursor ions was set to 20 ppm for both the first search and the main search, and the mass tolerance for fragment ions was set to 20 ppm. Carbamidomethylation of cysteine (C) was specified as a fixed modification, while oxidation of methionine (M), acetylation at the protein N‐terminus, and crotonylation of lysine (K) were specified as variable modifications. The FDR was controlled at < 1% at the protein, peptide and peptide‐spectrum match (PSM) levels.

Thirteen target peptides were quantified by PRM using Skyline (v. 21.1). The enzyme specificity was set to trypsin [KR/P], allowing up to 2 missed cleavages. Peptide length ranged from 7 to 25 amino acids, with a maximum of 3 variable modifications. Precursor ion charges of 2 and 3 were selected, and fragment ions included *b*, *y* and *p* ions with charges of 1 and 2. Product ions from ion 3 to the last ion were considered, with an ion match tolerance of 0.02 Da.

### 4.6 Bioinformatics Analysis

The identified proteins were annotated for subcellular localisation using the WoLF PSORT software, followed by classification and statistical analysis. Gene function annotation was performed using eggNOG‐mapper (http://eggnog‐mapper.embl.de), and the KEGG and GO enrichment results were visualised with TBtools‐II (Chen et al. 2023). Protein–protein interactions for differentially expressed modified proteins were identified using STRING v. 12.0 (https://string‐db.org), with the minimum required interaction score set to high confidence (0.7) and other parameters left at their default values. The results were visualised using Cytoscape v. 3.10.2, and network analysis was performed using the built‐in algorithms of the software.

We performed motif analysis on all 10,523 protein modification sites as a single category, using lysine as the core amino acid. The analysis considered the 10 amino acids both upstream and downstream of the core lysine. The modified lysine residues were treated as the positive dataset, while all lysine‐containing peptides were used as the reference dataset. Differences between the datasets were calculated based on the iceLogo (Colaert et al. 2009) method, which measures the proportional difference of each amino acid at the same position between the positive and reference datasets. The *p*‐values were then calculated using a binomial test. We used NetSurfP (Hoie et al. 2022) to analyse the secondary structures of modified amino acids in proteins. For both modified and unmodified amino acids, we calculated the average probabilities and surface accessibility across the three types of secondary structures. The Wilcoxon rank‐sum test was applied to evaluate significant differences, with *p*‐values determining the statistical significance. At the same time, we used the MoMo tool based on the motif‐x algorithm to analyse the motif characteristics of the modification sites (https://meme‐suite.org/meme/tools/momo). The analysis focused on peptide sequences comprising 10 amino acids upstream and downstream of each identified modification site. We compared these sequences with all potential modification sites in wheat, using default parameters for the analysis.

The full genome data for *Triticum aestivum* ‘Chinese Spring’ was based on the research by Csiszar et al. (2012) and Zhang et al. (2022). PRX and CAT family genes were searched for across the genomes of wheat (https://wheat‐urgi.versailles.inra.fr/Seq‐Repository/Assemblies), barley ( *Hordeum vulgare* , https://www.ncbi.nlm.nih.gov/datasets/genome/GCF_904849725.1/), rice ( *Oryza sativa* , http://rice.uga.edu/downloads_gad.shtml), maize ( *Zea mays* , https://download.maizegdb.org/Zm‐B73‐REFERENCE‐NAM‐5.0/) and *A. thaliana* (https://www.arabidopsis.org/download/list?dir=Sequences), retaining only the sister clades closely related to TaPRXIIB or TaCAT2. The sequences were aligned using the MUSCLE algorithm and a phylogenetic tree was constructed using the neighbour‐joining method, with the bootstrap value set to 1000 (Kumar et al. 2018).

### 4.7 Enzyme Activity Assay

Target proteins (Table S7) were transiently expressed in *N*. *benthamiana*, extracted and purified (Zhang, Chen, et al. 2020). POD activity was measured by monitoring absorbance at 470 nm, and activity was calculated relative to protein concentration. Three biological replicates were performed.

### 4.8 Molecular Docking Simulation Analysis

All protein structures were predicted using AlphaFold (Abramson et al. 2024), with ligand files sourced from PubChem (https://pubchem.ncbi.nlm.nih.gov). Protein structures were prepared by removing water molecules and metal ions, followed by the addition of polar hydrogens using AutoDock Tools (v. 1.5.6). The processed proteins were used as receptor models. The ligand was prepared by adding hydrogens, assigning Gasteiger charges, and defining rotatable bonds. Molecular docking of the ligand and receptors was performed using AutoDock4 (https://autodock.scripps.edu/download‐autodock4/). A grid box was defined to cover the entire protein structure, with the grid spacing set to 0.375 Å. Grid maps were calculated using AutoGrid4. Docking simulations were performed using the Lamarckian genetic algorithm (Lamarckian GA 4.2), with default parameters, including an initial population size of 150, a maximum number of 2.5 × 10^6^ energy evaluations, and 10 independent docking runs. Semiflexible docking was applied. AutoDock4.exe was used to read the parameters and perform the docking calculations. The results were comprehensively analysed using the Analysis tool, with binding energy and inhibition constant as the evaluation criteria (a larger absolute value of binding energy combined with a smaller inhibition constant indicates a stronger binding between the receptor and ligand) (Hulme and Trevethick 2010). Intermolecular interactions were analysed, and the conformations of ligand‐receptor complexes were visualised using PyMOL software.

## Author Contributions

**Tianjie Sun:** writing – review and editing, supervision, project administration. **Na Liu:** resources, project administration. **Rongna Wang:** conceptualisation, funding acquisition, writing – review and editing, supervision. **Dongmei Wang:** conceptualisation, funding acquisition, writing – review and editing, supervision, resources, project administration. **Zelin Niu:** validation. **Jia Gu:** investigation, resources. **Shengfang Han:** funding acquisition, resources. **Qipeng Wang:** conceptualisation, writing – original draft, methodology, visualization, formal analysis. **Qian Wang:** validation, formal analysis.

## Funding

This work was supported by the National Natural Science Foundation of China (31871548, 32372047 and 32201706), the Natural Science Foundation of Hebei Province (C2023204054), the S&T Program of Hebei (23567601H), the Basic Research Funds for Provincial Universities in Hebei Province (KY2022036), the Science and Technology Research Projects of Hebei Universities (QN2024024) and the Central Guiding Local Science and Technology Development Fund Project (236Z6502G).

## Conflicts of Interest

The authors declare no conflicts of interest.

## Supporting information


**Figure S1:** Identification of Kcr in incompatible wheat–*Puccinia triticina* combinations and the effects of different concentrations of deacylase inhibitor trichostatin A (TSA) on wheat and *P. triticina*.
(a) Western blotting with pan anti‐crotonyllysine antibody. (b) Coomassie brilliant blue staining. (c) Effects of different TSA concentrations on wheat leaves. (d) Microscopic observation of *P. triticina* morphology after TSA treatment. Results observed at higher magnification are shown in the inset. (e–g) Quantitative results of the effects of different TSA concentrations on the physiological state of *P. triticina*. Different lowercase letters indicate significant differences (*p* < 0.05), as determined by one‐way ANOVA followed by Duncan's multiple range test.

**Figure S2:** Quality control analysis of crotonylation proteomics data.
(a) Distribution of peptide lengths identified by mass spectrometry. (b) Mass error analysis of peptide identification in mass spectrometry. The vertical axis shows peptide scores, and the horizontal axis displays the deviation between observed and theoretical mass‐to‐charge ratios. (c) Principal component analysis results of the six samples. The clustering of samples reflects the extent of differences between them. Different colours represent the major categories of sample classification. (d) Significantly (motif score > 15) enriched motif sequences around lysine crotonylation sites. (e) and (f) Distribution of all lysines and crotonylated lysines in structured protein regions.

**Figure S3:** Quantitative analysis and expression patterns of key differentially modified proteins in wheat during *Puccinia triticina* infection.
(a) Targeted quantitative analysis of key differentially modified proteins using PRM mass spectrometry. Different colours of the rectangles indicate the peak areas of fragment ions for the selected peptides. The dotp values represent the similarity between the measured fragment spectrum and the library spectrum. (b) Analysis results of key differentially modified proteins in the modification proteomics. Significant differences are indicated by *, ** and ***, corresponding to *p* < 0.05, *p* < 0.01 and *p* < 0.001, respectively. (c) Relative expression pattern of key differentially modified proteins in incompatible and compatible combinations of Tc*Lr26*. Values are from three independent replicates. Lowercase letters (purple for Tc*Lr26*‐165; green for Tc*Lr26*‐260) indicate significant differences (*p* < 0.05), as determined by one‐way ANOVA followed by Duncan's multiple range test.

**Figure S4:** Comparative analysis of Kcr sites in TaPRXIIB and TaCAT2 and their most closely related homologues in other species.
(a) and (b) Conservation analysis of modification sites in TaPRXIIB and TaCAT2 and their homologues in closely related species. Conserved modification sites are highlighted with an orange background. Red denotes upregulation of modifications at 24 h, blue shows downregulation and grey indicates no change.

**Figure S5:** Observation and sequence analysis of *TaPRXIIB*‐silenced plants.
(a) Virus symptoms (chlorosis or mosaic) were observed on wheat leaves 12 days after inoculation with BMSV:00, BMSV:*TaPDS*, or BMSV:*TaPRXIIB*. MOCK: wheat leaves treated with FES buffer. The BSMV RNA fragments consist of α, β and γ0. (b) Silencing efficiency of *TaPRXIIB_Rg* in virus‐induced gene silencing experiments. Values are from three independent replicates. (c) Sequence alignment among the silenced genes. The primer regions used to assess silencing efficiency are highlighted in blue. The regions containing the silenced fragments are highlighted in grey. Red dashed lines mark the corresponding primer regions.

**Figure S6:** Validation of crotonylation at lysine 172 in TaPRXIIB.
(a) Detection results of pan‐crotonylation antibody. (b) Detection results of GFP antibody.

**Table S1:** Statistics of histones and non‐histones.
24HSP: Values were available only at 24 h among the time points of 0 and 24 h; 0HSP: Values were available only at 0 h among the time points of 0 and 24 h; 24HUP: 24 h modification is upregulated, with FC > 1.5 compared to 0 h; 24HDN: 24 h modification is downregulated, with FC > 1.5 compared to 0 h.

**Table S2:** Comparison of this study with other studies on crotonylation modification.

**Table S3:** Information on differentially modified proteins.

**Table S4:** Information on the interaction network of crotonylated proteins.

**Table S5:** Information on the interaction network of differentially modified antioxidant enzymes and their interactors.

**Table S6:** Real‐time quantitative PCR primers and related information used for gene detection.

**Table S7:** Information on primers for full‐length gene cloning, site‐directed mutagenesis and overexpression.

**Table S8:** Information on primers for BMSV vector construction and gene silencing efficiency assessment.

## Data Availability

The data supporting the findings of this study are available in the Supporting Information of this article.
