# Supplementary material for: Global Crotonylome Profiling Identifies TaPRXIIB Crotonylation as a Modulator H2O2 Homeostasis in Wheat Resistance to Puccinia triticina
Source: Mol Plant Pathol. 2026 Jul 11;27(7):e70288. doi: 10.1111/mpp.70288 (PMC13354946; doi:10.1111/mpp.70288)
Supplement: Supplementary file 1 — Figure S1: Identification of Kcr in incompatible wheat–Puccinia triticina combinations and the effects of different concentrations of deacylase inhibitor trichostatin A (TSA) on wheat and P. triticina. (a) Western blotting with pan anti‐crotonyllysine antibody. (b) Coomassie brilliant blue staining. (c) Effects of different TSA concentrations on wheat leaves. (d) Microscopic observation of P. triticina morphology after TSA treatment. Results observed at higher magnification are shown in the inset. (e–g) Quantitative results of the effects of different TSA concentrations on the physiological state of P. triticina. Different lowercase letters indicate significant differences (p < 0.05), as determined by one‐way ANOVA followed by Duncan's multiple range test. [file MPP-27-e70288-s009.docx]

**
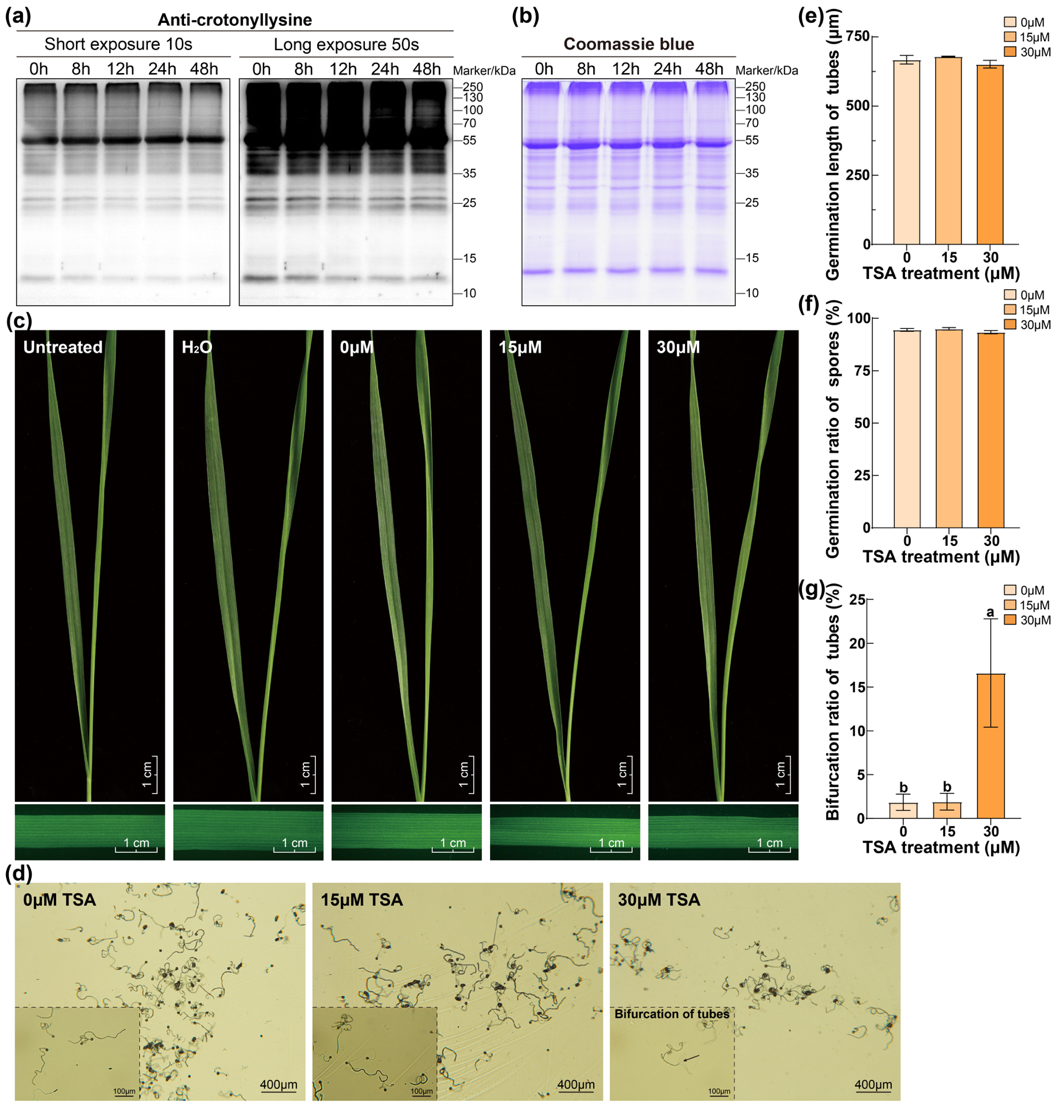
**

**Figure S1 Identification of Kcr in incompatible wheat-*Pt* combinations and the effects of different concentrations of TSA on wheat and *Pt*.**

(a) Western blotting with pan anti-crotonyllysine antibody. (b) Coomassie brilliant blue staining. (c) Effects of different TSA concentrations on wheat leaves. (d) Microscopic observation of *Pt* morphology after TSA treatment. Results observed at higher magnification are shown in the inset. (e-g) Quantitative results of the effects of different TSA concentrations on the physiological state of *Pt*. Different lowercase letters indicate significant differences (*P*<0.05), as determined by one-way ANOVA followed by Duncan’s multiple range test.
