# Supplementary material for: Global Crotonylome Profiling Identifies TaPRXIIB Crotonylation as a Modulator H2O2 Homeostasis in Wheat Resistance to Puccinia triticina
Source: Mol Plant Pathol. 2026 Jul 11;27(7):e70288. doi: 10.1111/mpp.70288 (PMC13354946; doi:10.1111/mpp.70288)
Supplement: Supplementary file 2 — Figure S2: Quality control analysis of crotonylation proteomics data. (a) Distribution of peptide lengths identified by mass spectrometry. (b) Mass error analysis of peptide identification in mass spectrometry. The vertical axis shows peptide scores, and the horizontal axis displays the deviation between observed and theoretical mass‐to‐charge ratios. (c) Principal component analysis results of the six samples. The clustering of samples reflects the extent of differences between them. Different colours represent the major categories of sample classification. (d) Significantly (motif score > 15) enriched motif sequences around lysine crotonylation sites. (e) and (f) Distribution of all lysines and crotonylated lysines in structured protein regions. [file MPP-27-e70288-s002.docx]

**
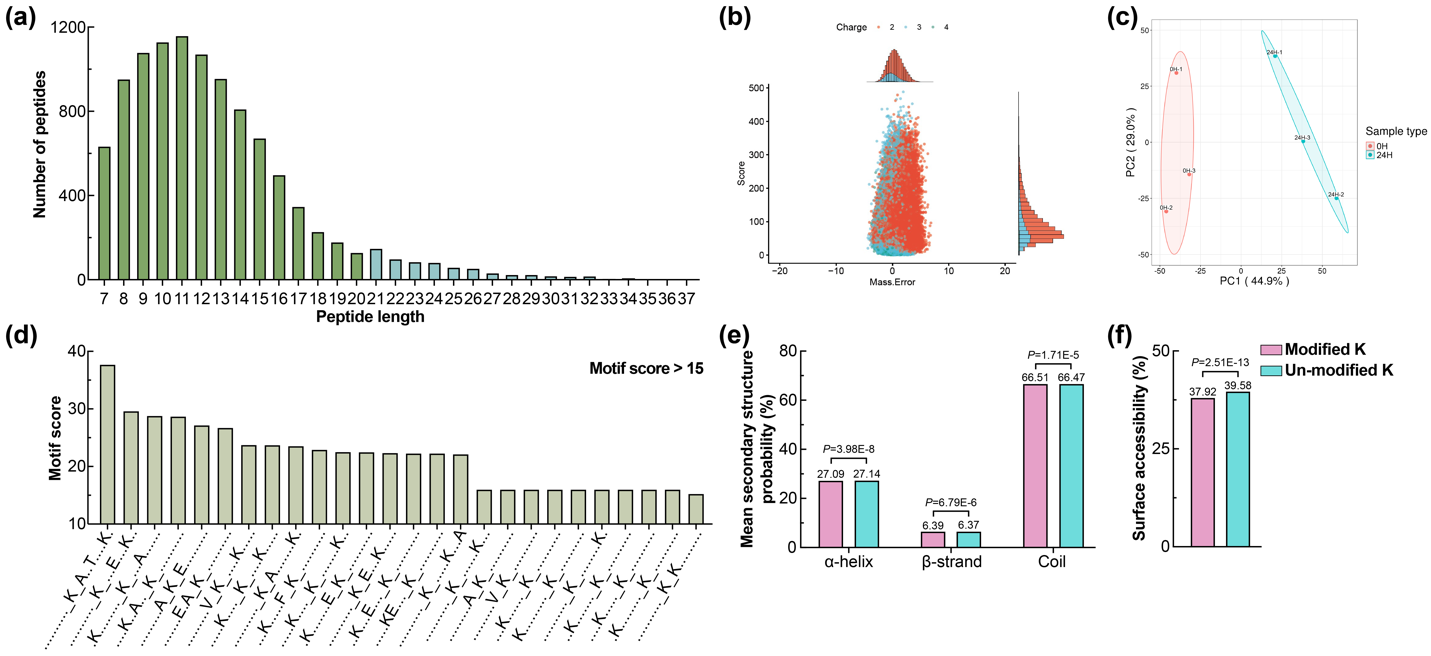
**

**Figure S2 Quality control analysis of crotonylation proteomics data.**

(a) Distribution of peptide lengths identified by mass spectrometry. (b) Mass error analysis of peptide identification in mass spectrometry. The vertical axis shows peptide scores, and the horizontal axis displays the deviation between observed and theoretical mass-to-charge ratios. (c) PCA analysis results of the 6 samples. The clustering of samples reflects the extent of differences between them. Different colors represent the major categories of sample classification. (d) Significantly (motif score>15) enriched motif sequences around lysine crotonylation sites. (e) and (f) Distribution of all lysines and crotonylated lysines in structured protein regions.
