# Supplementary material for: Global Crotonylome Profiling Identifies TaPRXIIB Crotonylation as a Modulator H2O2 Homeostasis in Wheat Resistance to Puccinia triticina
Source: Mol Plant Pathol. 2026 Jul 11;27(7):e70288. doi: 10.1111/mpp.70288 (PMC13354946; doi:10.1111/mpp.70288)
Supplement: Supplementary file 3 — Figure S3: Quantitative analysis and expression patterns of key differentially modified proteins in wheat during Puccinia triticina infection. (a) Targeted quantitative analysis of key differentially modified proteins using PRM mass spectrometry. Different colours of the rectangles indicate the peak areas of fragment ions for the selected peptides. The dotp values represent the similarity between the measured fragment spectrum and the library spectrum. (b) Analysis results of key differentially modified proteins in the modification proteomics. Significant differences are indicated by *, ** and ***, corresponding to p < 0.05, p < 0.01 and p < 0.001, respectively. (c) Relative expression pattern of key differentially modified proteins in incompatible and compatible combinations of TcLr26. Values are from three independent replicates. Lowercase letters (purple for TcLr26‐165; green for TcLr26‐260) indicate significant differences (p < 0.05), as determined by one‐way ANOVA followed by Duncan's multiple range test. [file MPP-27-e70288-s014.docx]

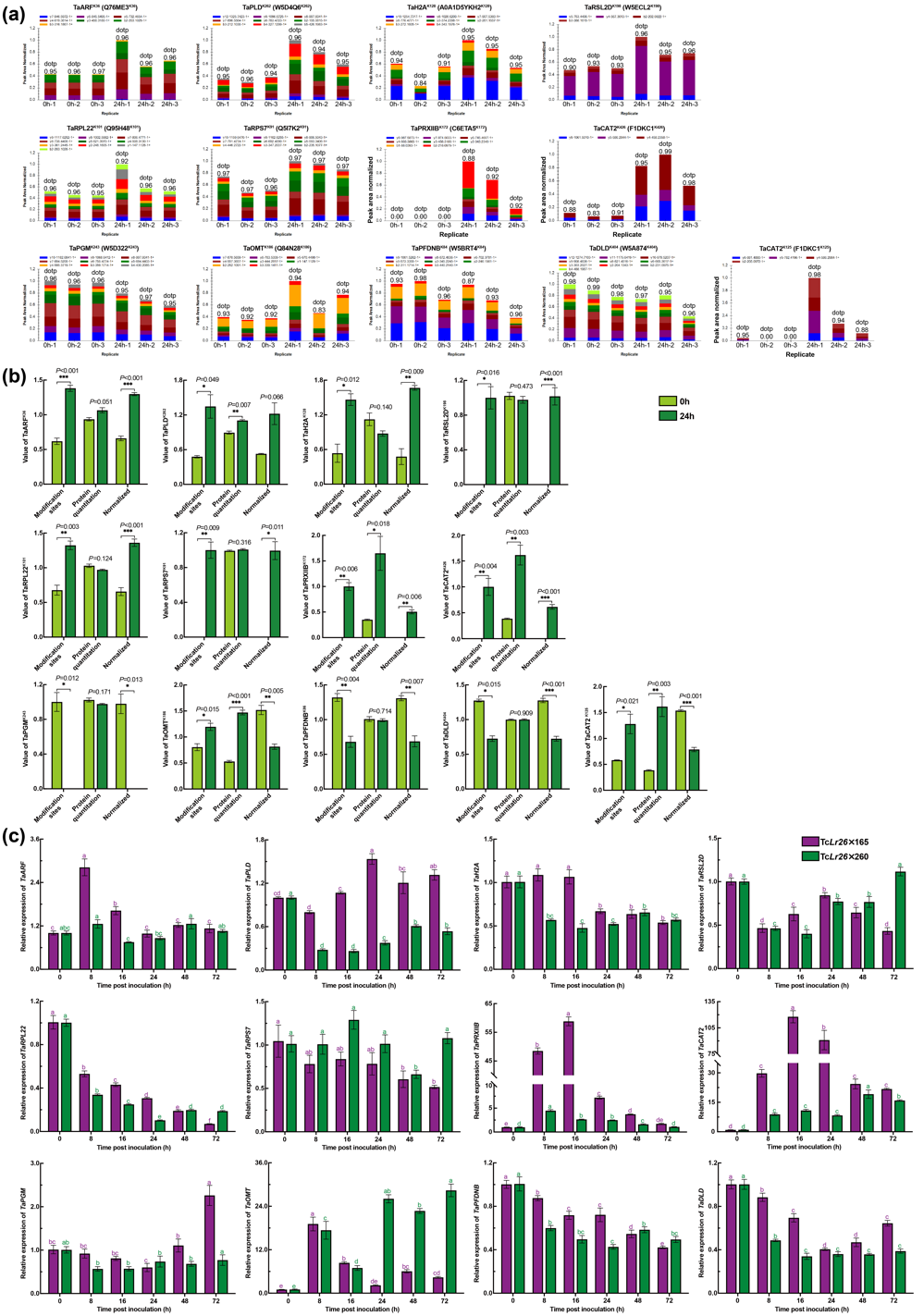


**Figure S3 Quantitative analysis and expression patterns of key differentially modified proteins in wheat during *Pt* infection.**

(a) Targeted quantitative analysis of key differentially modified proteins using PRM mass spectrometry. Different colors of the rectangles indicate the peak areas of fragment ions for the selected peptides. The dotp values represent the similarity between the measured fragment spectrum and the library spectrum. (b) Analysis results of key differentially modified proteins in the modification proteomics. Significant differences are indicated by *, **, and ***, corresponding to *P*<0.05, *P*<0.01, and *P*<0.001, respectively. (c) Relative expression pattern of key differentially modified proteins in incompatible and compatible combinations of Tc*Lr26*. Values are from 3 independent replicates. Lowercase letters (purple for Tc*Lr26*-165; green for Tc*Lr26*-260) indicate significant differences (*P*<0.05), as determined by one-way ANOVA followed by Duncan’s multiple range test.
