# Supplementary material for: Global Crotonylome Profiling Identifies TaPRXIIB Crotonylation as a Modulator H2O2 Homeostasis in Wheat Resistance to Puccinia triticina
Source: Mol Plant Pathol. 2026 Jul 11;27(7):e70288. doi: 10.1111/mpp.70288 (PMC13354946; doi:10.1111/mpp.70288)
Supplement: Supplementary file 4 — Figure S4: Comparative analysis of Kcr sites in TaPRXIIB and TaCAT2 and their most closely related homologues in other species. (a) and (b) Conservation analysis of modification sites in TaPRXIIB and TaCAT2 and their homologues in closely related species. Conserved modification sites are highlighted with an orange background. Red denotes upregulation of modifications at 24 h, blue shows downregulation and grey indicates no change. [file MPP-27-e70288-s004.docx]

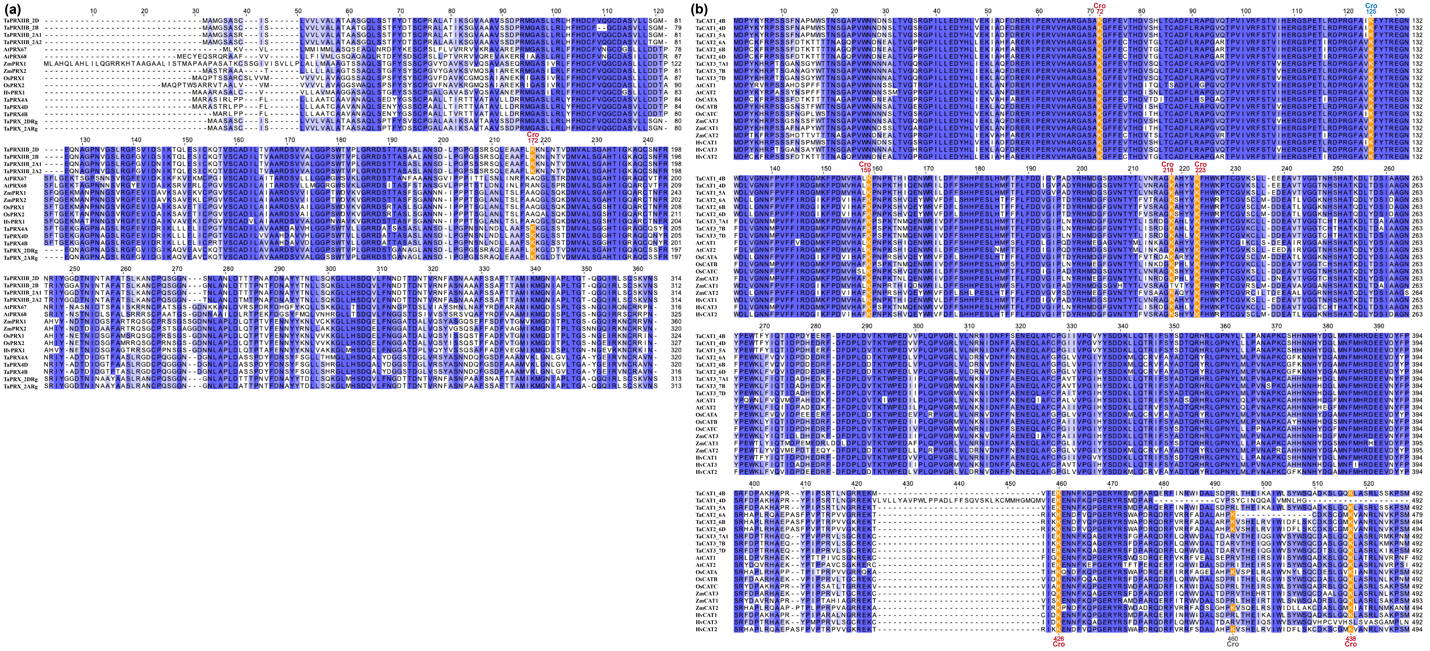


**Figure S4 Comparative analysis of Kcr sites in TaPRXⅡB and TaCAT2 and their most closely related homologs in other species.**

(a) and (b) Conservation analysis of modification sites in TaPRXⅡB and TaCAT2 and their homologs in closely related species. Conserved modification sites are highlighted with an orange background. Red denotes upregulation of modifications at 24 h, blue shows downregulation, and gray indicates no change.
