# Supplementary material for: Global Crotonylome Profiling Identifies TaPRXIIB Crotonylation as a Modulator H2O2 Homeostasis in Wheat Resistance to Puccinia triticina
Source: Mol Plant Pathol. 2026 Jul 11;27(7):e70288. doi: 10.1111/mpp.70288 (PMC13354946; doi:10.1111/mpp.70288)
Supplement: Supplementary file 5 — Figure S5: Observation and sequence analysis of TaPRXIIB‐silenced plants. (a) Virus symptoms (chlorosis or mosaic) were observed on wheat leaves 12 days after inoculation with BMSV:00, BMSV:TaPDS, or BMSV:TaPRXIIB. MOCK: wheat leaves treated with FES buffer. The BSMV RNA fragments consist of α, β and γ0. (b) Silencing efficiency of TaPRXIIB_Rg in virus‐induced gene silencing experiments. Values are from three independent replicates. (c) Sequence alignment among the silenced genes. The primer regions used to assess silencing efficiency are highlighted in blue. The regions containing the silenced fragments are highlighted in grey. Red dashed lines mark the corresponding primer regions. [file MPP-27-e70288-s011.docx]

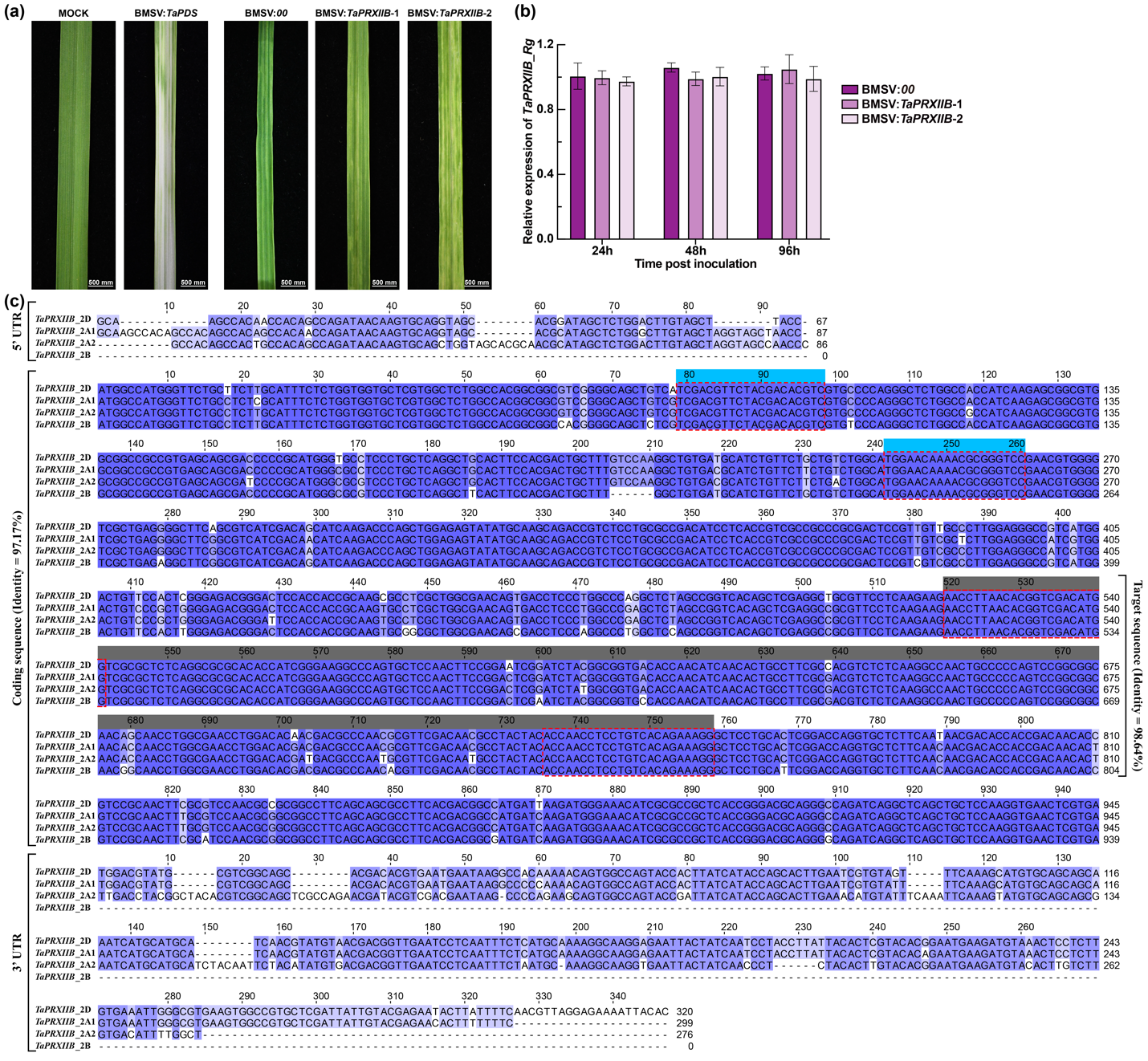


**Figure S5 Observation and sequence analysis of *TaPRXⅡB*-silenced plants.**

(a) Virus symptoms (chlorosis or mosaic) were observed on wheat leaves 12 days after inoculation with BMSV:*00*, BMSV:*TaPDS*, or BMSV:*TaPRXⅡB*. MOCK: wheat leaves treated with FES buffer. The BSMV RNA fragments consist of α, β, and γ0. (b) Silencing efficiency of *TaPRXⅡB_Rg* in VIGS experiments. Values are from 3 independent replicates. (c) Sequence alignment among the silenced genes. The primer regions used to assess silencing efficiency are highlighted in blue. The regions containing the silenced fragments are highlighted in gray. Red dashed lines mark the corresponding primer regions.
