# Supplementary material for: Global Crotonylome Profiling Identifies TaPRXIIB Crotonylation as a Modulator H2O2 Homeostasis in Wheat Resistance to Puccinia triticina
Source: Mol Plant Pathol. 2026 Jul 11;27(7):e70288. doi: 10.1111/mpp.70288 (PMC13354946; doi:10.1111/mpp.70288)
Supplement: Supplementary file 6 — Figure S6: Validation of crotonylation at lysine 172 in TaPRXIIB. (a) Detection results of pan‐crotonylation antibody. (b) Detection results of GFP antibody. [file MPP-27-e70288-s003.docx]

**
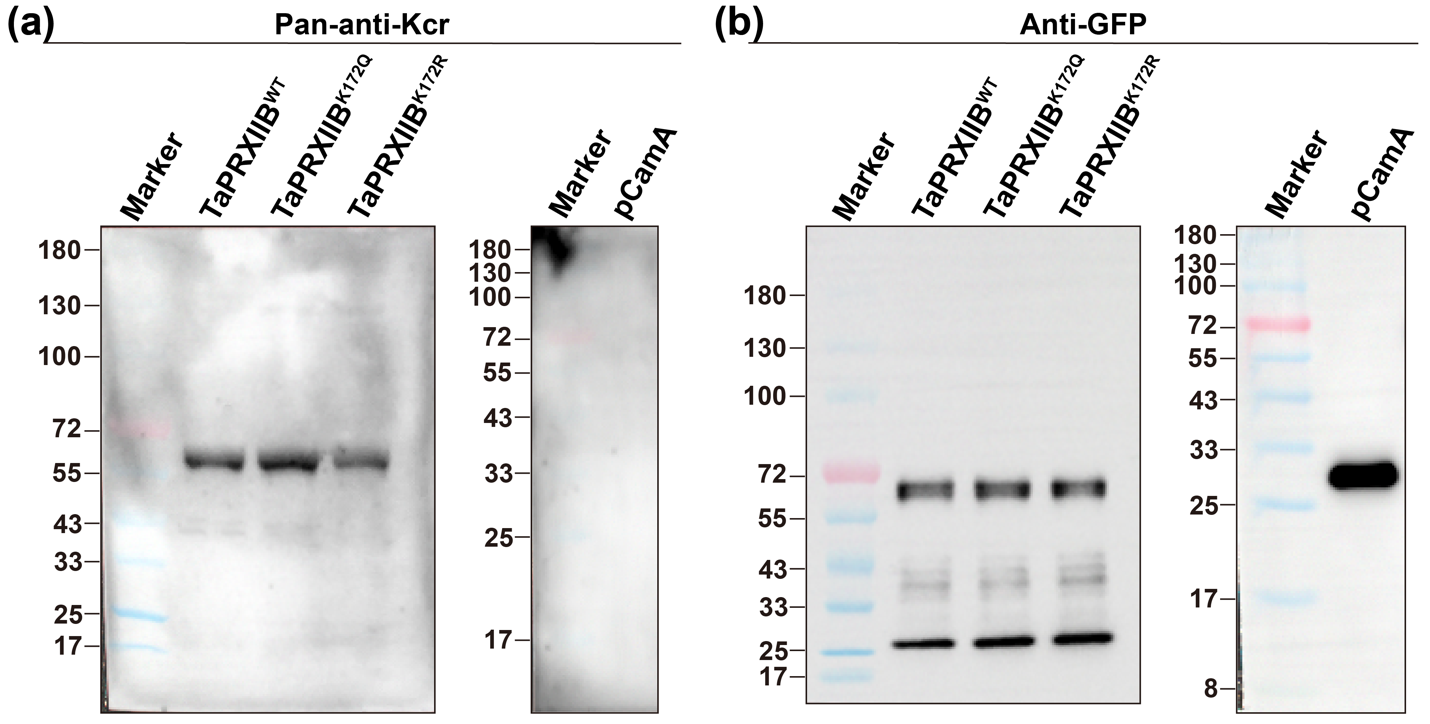
**

**Fig. S6 Validation of crotonylation at lysine 172 in TaPRXⅡB.**

(a) Detection results of pan-crotonylation antibody. (b) Detection results of GFP antibody.
