# Supplementary material for: Global Crotonylome Profiling Identifies TaPRXIIB Crotonylation as a Modulator H2O2 Homeostasis in Wheat Resistance to Puccinia triticina
Source: Mol Plant Pathol. 2026 Jul 11;27(7):e70288. doi: 10.1111/mpp.70288 (PMC13354946; doi:10.1111/mpp.70288)
Supplement: Supplementary file 7 — Table S1: Statistics of histones and non‐histones. 24HSP: Values were available only at 24 h among the time points of 0 and 24 h; 0HSP: Values were available only at 0 h among the time points of 0 and 24 h; 24HUP: 24 h modification is upregulated, with FC > 1.5 compared to 0 h; 24HDN: 24 h modification is downregulated, with FC > 1.5 compared to 0 h. [file MPP-27-e70288-s007.docx]

| **Table S1 Statistics of histones and non-histones** | | | | |
| --- | --- | --- | --- | --- |
| Category | Histone | | Non-histone | |
|  | Site | Protein | Site | Protein |
| All-identified | 156 | 62 | 10367 | 2976 |
| All-quantifiable | 7 | 4 | 3326 | 948 |
| 24HSP | 0 | 0 | 702 | 486 |
| 0HSP | 4 | 3 | 637 | 436 |
| 24HUP | 1 | 1 | 140 | 111 |
| 24HDN | 0 | 0 | 198 | 161 |
| 24HSP: Values were available only at 24 h among the time points of 0 and 24 h; 0HSP: Values were available only at 0 h among the time points of 0 and 24 h; 24HUP: 24 h modification is upregulated, with FC>1.5 compared to 0 h; 24HDN: 24 h modification is downregulated, with FC>1.5 compared to 0 h. | | | | |
