# Supplementary material for: Global Crotonylome Profiling Identifies TaPRXIIB Crotonylation as a Modulator H2O2 Homeostasis in Wheat Resistance to Puccinia triticina
Source: Mol Plant Pathol. 2026 Jul 11;27(7):e70288. doi: 10.1111/mpp.70288 (PMC13354946; doi:10.1111/mpp.70288)
Supplement: Supplementary file 8 — Table S2: Comparison of this study with other studies on crotonylation modification. [file MPP-27-e70288-s012.docx]

| **Table S2 Comparison of this study with other studies on crotonylation modification** | | | | | | |
| --- | --- | --- | --- | --- | --- | --- |
| Species | Types | No. of proteins | No. of sites | Average | References | Year |
| *Triticum* *aestivum* | Leaf rust disease | 3,038 | 10,523 | 3.46 | This study | 2025 |
| *Zjziphus jujuba* | Phytoplasma disease | 1,656 | 3,900 | 2.36 | Zhang *et al*. | 2024 |
| *Triticum* *aestivum* | Cold stress | 1,726 | 4,696 | 2.72 | Zhang *et al.* | 2023 |
| *Triticum* *aestivum* | Salt stress | 695 | 1,785 | 2.57 | Zhu *et al.* | 2023 |
| *Dendrobium huoshanense* | Drought stress | 1,591 | 4,726 | 2.97 | Wu *et al.* | 2022 |
| *Dendranthema grandiforum* | Cold stress | 1,199 | 2,017 | 1.68 | Lin *et al.* | 2021 |

References

Lin, P., Bai, H. R., He, L., Huang, Q. X., Zeng, Q. H., Pan, Y. Z.*, et al.* (2021) Proteome-wide and Lysine Crotonylation Profiling Reveals the Importance of Crotonylation in Chrysanthemum (*Dendranthema grandiforum*) under Low-temperature. *BMC Genomics,* **22,** 51.

Wu, J., Meng, X., Jiang, W., Wang, Z., Zhang, J., Meng, F.*, et al.* (2022) Qualitative Proteome-wide Analysis Reveals the Diverse Functions of Lysine Crotonylation in *Dendrobium huoshanense*. *Frontiers in Plant Science,* **13,** 822374.

Zhang, L., Wang, H., Xue, C., Liu, Y., Zhang, Y., Liu, Z.*, et al.* (2024) The Crotonylated and Succinylated Proteins of Jujube Involved in Phytoplasma-stress Responses. *BMC Biology,* **22,** 113.

Zhang, N., Wang, S., Zhao, S., Chen, D., Tian, H., Li, J.*, et al.* (2023) Global Crotonylatome and GWAS Revealed a *TaSRT1*-*TaPGK* Model Regulating Wheat Cold Tolerance Through Mediating Pyruvate. *Science Advances,* **9,** eadg1012.

Zhu, D., Liu, J., Duan, W., Sun, H., Zhang, L., Yan, Y.*, et al.* (2023) Analysis of the Chloroplast Crotonylome of Wheat Seedling Leaves Reveals the Roles of Crotonylated Proteins Involved in Salt-stress Responses. *Journal of Experimental Botany,* **74,** 2067–2082.
