# Supplementary material for: Global Crotonylome Profiling Identifies TaPRXIIB Crotonylation as a Modulator H2O2 Homeostasis in Wheat Resistance to Puccinia triticina
Source: Mol Plant Pathol. 2026 Jul 11;27(7):e70288. doi: 10.1111/mpp.70288 (PMC13354946; doi:10.1111/mpp.70288)
Supplement: Supplementary file 9 — Table S3: Information on differentially modified proteins. [file MPP-27-e70288-s006.docx]

| **Table S3 Information on differentially modified proteins** | | | | | |
| --- | --- | --- | --- | --- | --- |
| Category | Accession | Description | Category | Accession | Description |
| 24HSP | Q7DLM1 | (1,31,4) beta glucanase | 24HSP | A0A0C4BKA8 | AAA domain-containing protein |
| 24HSP | A0A3B6MN48 | 1-acylglycerol-3-phosphate O-acyltransferase | 24HSP | A0A3B6NSE6 | AAA domain-containing protein |
| 24HSP | A0A3B6IVI8 | 15-cis-phytoene desaturase, chloroplastic/chromoplastic | 24HSP | A0A3B6CEY1 | AB hydrolase-1 domain-containing protein |
| 24HSP | A0A3B6H4R5 | 2-hydroxyacyl-CoA lyase | 24HSP | A0A3B6ISQ8 | ABC transporter domain-containing protein |
| 24HSP | A0A3B6QHC3 | 2-oxoglutarate (2OG) and Fe(II)-dependent oxygenase superfamily protein | 24HSP | A0A2P1AAU6 | ABCF1d |
| 24HSP | A0A341PIP8 | 2-oxoglutarate/malate translocator | 24HSP | A0A3B6DDG1 | Abscisic stress-ripening protein 2 |
| 24HSP | W5EUY8 | 25.3 kDa vesicle transport protein, putative, expressed | 24HSP | A0A3B6I0D2 | ACB domain-containing protein |
| 24HSP | A0A3B6DIW5 | 26S proteasome non-ATPase regulatory subunit 1 homolog | 24HSP | B2ZGL4 | Acetyl-CoA carboxylase |
| 24HSP | A0A3B6QEK4 | 3-hydroxyacyl-CoA dehydrogenase | 24HSP | A0A3B5XV05 | Acetylornithine transaminase |
| 24HSP | A0A3B6B8V8 | 3-isopropylmalate dehydrogenase | 24HSP | A0A3B6KT79 | Aconitate hydratase |
| 24HSP | A0A3B6PSZ6 | 3-ketoacyl-CoA thiolase-like protein | 24HSP | A0A3chineseB6GS53 | Aconitate hydratase |
| 24HSP | A0A3B6NV46 | 3-ketoacyl-CoA thiolase-like protein | 24HSP | A0A3B6FKB3 | Aconitate hydratase |
| 24HSP | A0A3B6MXK0 | 30S ribosomal protein S18, chloroplastic | 24HSP | A0A3B6FVN1 | Acyl-[acyl-carrier-protein] desaturase 2, chloroplastic |
| 24HSP | P17933 | 30S ribosomal protein S2, chloroplastic | 24HSP | A0A3B6D774 | Acyl-CoA dehydrogenase family member 10 |
| 24HSP | Q95H49 | 30S ribosomal protein S3, chloroplastic | 24HSP | W5ALC0 | Acyl-coenzyme A oxidase 4, peroxisomal |
| 24HSP | W5FPA7 | 30S ribosomal protein S4, chloroplastic | 24HSP | Q43199 | Adenine phosphoribosyltransferase 1 |
| 24HSP | A0A3B6QF97 | 33 kDa ribonucleoprotein, chloroplastic | 24HSP | A0A3B6CE50 | Adenosylhomocysteinase |
| 24HSP | A0A3B6AU50 | 4-alpha-glucanotransferase | 24HSP | A0A3B6TT87 | Adenylate kinase |
| 24HSP | A0A3B6HW46 | 4-hydroxy-3-methylbut-2-enyl diphosphate reductase | 24HSP | A0A3B6I0G2 | Adenylyl cyclase-associated protein |
| 24HSP | Q1XIR9 | 4-hydroxy-7-methoxy-3-oxo-3,4-dihydro-2H-1,4-benzoxazin-2-yl glucoside beta-D-glucosidase 1a, chloroplastic | 24HSP | A0A3B6KMV4 | ADF-H domain-containing protein |
| 24HSP | A0A3B6LP26 | 4-hydroxy-7-methoxy-3-oxo-3,4-dihydro-2H-1,4-benzoxazin-2-yl glucosidebeta-D-glucosidase | 24HSP | A0A1D5ZLT8 | ADF-H domain-containing protein |
| 24HSP | A0A0C4BIW4 | 40S ribosomal protein S14 | 24HSP | W5EH87 | ADP-ribosylation factor-like protein 8B |
| 24HSP | W5FFT4 | 40S ribosomal protein S16 | 24HSP | A0A3B6EGC7 | ADP, ATP carrier protein |
| 24HSP | W5DS33 | 40S ribosomal protein S16 | 24HSP | A0A3B6NPW7 | ADP, ATP carrier protein |
| 24HSP | A0A3B6KQB8 | 40S ribosomal protein S26 | 24HSP | A0A3B6HMZ7 | Alanine-glyoxylate aminotransferase |
| 24HSP | W5C8N6 | 40S ribosomal protein S6 | 24HSP | A0A3B6C739 | Alba domain-containing protein |
| 24HSP | Q5I7K2 | 40S ribosomal protein S7 | 24HSP | A0A3B5ZNL5 | Alcohol dehydrogenase-like 3 |
| 24HSP | A0A1D5UKZ4 | 40S ribosomal protein S8 | 24HSP | A0A3B6KIN5 | Aldedh domain-containing protein |
| 24HSP | A0A3B6C534 | 40S ribosomal protein SA | 24HSP | A0A3B6MMH5 | Aldo_ket_red domain-containing protein |
| 24HSP | W5ELX6 | 4D hexose transporter | 24HSP | A0A3B6PFK2 | Aldo_ket_red domain-containing protein |
| 24HSP | A0A3B6KU60 | 50S ribosomal protein L11, chloroplastic | 24HSP | A0A3B6AQ54 | Aldo_ket_red domain-containing protein |
| 24HSP | A0A3B6QHZ8 | 50S ribosomal protein L19, chloroplastic | 24HSP | W5DNK7 | Alkaline/neutral invertase |
| 24HSP | P11534 | 50S ribosomal protein L2, chloroplastic | 24HSP | A0A3B6Q8W6 | Alkylresorcinol synthase |
| 24HSP | A0A3B6C9L1 | 50S ribosomal protein L20 | 24HSP | A0A3B6KQ72 | Alpha-mannosidase |
| 24HSP | A0A3B5ZNR7 | 50S ribosomal protein L28, chloroplastic | 24HSP | A0A3B6RBU0 | Alternative NAD(P)H-ubiquinone oxidoreductase C1, chloroplastic/mitochondrial |
| 24HSP | Q95H56 | 50S ribosomal protein L33, chloroplastic | 24HSP | A0A3B6SJT5 | Amino_oxidase domain-containing protein |
| 24HSP | A0A3B5YUE8 | 60S ribosomal protein L11 | 24HSP | A0A3B6TV37 | Amino_oxidase domain-containing protein |
| 24HSP | W5FSM8 | 60S ribosomal protein L17-1 | 24HSP | A0A3B6I1M0 | Aminopeptidase |
| 24HSP | W5HTZ1 | 60S ribosomal protein L17-2 | 24HSP | W5AUD8 | Aminotran_1_2 domain-containing protein |
| 24HSP | A0A1D5SRX8 | 60S ribosomal protein L18a | 24HSP | D2KZ08 | Aminotransferase |
| 24HSP | W5HXT5 | 60S ribosomal protein L22-2 | 24HSP | A0A3B6TTS5 | Ananain |
| 24HSP | W5G990 | 60S ribosomal protein L31 | 24HSP | A0A3B5ZVQ5 | ANK_REP_REGION domain-containing protein |
| 24HSP | A0A3B6H174 | 60S ribosomal protein L36 | 24HSP | A0A3B6KJT5 | ANK_REP_REGION domain-containing protein |
| 24HSP | D8L9P6 | 60S ribosomal protein L37a, expressed | 24HSP | A0A3B6N401 | ANK_REP_REGION domain-containing protein |
| 24HSP | A0A3B6PN63 | 60S ribosomal protein L6 | 24HSP | A0A3B6J0Q3 | AP-2 complex subunit alpha |
| 24HSP | A0A3B6QGW3 | 60S ribosomal protein L6 | 24HSP | A0A3B6C9B6 | Arogenate dehydratase |
| 24HSP | W5GVR2 | 60S ribosomal protein L6 | 24HSP | A0A3B5Z514 | Asparagine synthetase [glutamine-hydrolyzing] |
| 24HSP | A0A077RSZ8 | 8-amino-7-oxononanoate synthase | 24HSP | A0A3B5XWA7 | Asparagine-tRNA ligase |
| 24HSP | A0A3B6GRN4 | AA_TRNA_LIGASE_II domain-containing protein | 24HSP | A0A077S3V2 | Aspartate aminotransferase |
| 24HSP | A0A3B6RMS3 | AAA domain-containing protein | 24HSP | A0A3B6MR53 | Aspartate-tRNA ligase |

| Category | Accession | Description | Category | Accession | Description |
| --- | --- | --- | --- | --- | --- |
| 24HSP | A0A1D5XT96 | ATP citrate synthase | 24HSP | A0A3B6IX48 | DEAD-box ATP-dependent RNA helicase 38 |
| 24HSP | Q332R4 | ATP synthase subunit alpha | 24HSP | A0A3B5XVD9 | Dehydroascorbate reductase |
| 24HSP | W5BEP1 | ATP synthase subunit d, mitochondrial | 24HSP | A0A3B6MVZ0 | DHQ_synthase domain-containing protein |
| 24HSP | A0A3B5ZT15 | ATP synthase subunit gamma | 24HSP | A0A3B6CHW0 | diacylglycerol kinase |
| 24HSP | A0A3B6GUS2 | ATP-dependent Clp protease proteolytic subunit | 24HSP | W5A874 | Dihydrolipoyl dehydrogenase |
| 24HSP | A0A3B6B6N9 | ATPase_AAA_core domain-containing protein | 24HSP | A0A3B6D590 | Dihydrolipoyllysine-residue succinyltransferase |
| 24HSP | A0A0M3LQU0 | Auxin-repressed protein | 24HSP | A0A3B5ZXC0 | DLH domain-containing protein |
| 24HSP | A0A3B6QGV0 | B-keto acyl reductase | 24HSP | A0A3B6GTT8 | DREPP4 protein |
| 24HSP | A0A3B6KCT5 | Bet_v_1 domain-containing protein | 24HSP | A0A3B6EFP8 | DREPP4 protein |
| 24HSP | A0A3B6KUD8 | Beta_elim_lyase domain-containing protein | 24HSP | A0A3B6FNR1 | DREPP4 protein |
| 24HSP | A0A3B6DNX1 | Beta-fructofuranosidase, insoluble isoenzyme 7 | 24HSP | A0A3B6KM77 | DUF3598 domain-containing protein |
| 24HSP | A0A2X0SDK6 | Caffeic acid 3-O-methyltransferase | 24HSP | W5ERE6 | Dynamin-related protein 1C |
| 24HSP | A0A3B6DB95 | Calcium-dependent protein kinase | 24HSP | A0A3B5XVE8 | E3 UFM1-protein ligase 1-like protein |
| 24HSP | A0A3B6PGD2 | Calnexin-like protein | 24HSP | A0A3B6MWN4 | Ecotropic viral integration site protein |
| 24HSP | A0A3B6PI06 | Calnexin-like protein | 24HSP | A0A3B6DB70 | EF1_GNE domain-containing protein |
| 24HSP | W5CT90 | Calreticulin | 24HSP | A0A1D5UZW7 | Elongation factor 1-alpha |
| 24HSP | A0A3B6PPE6 | Carboxypeptidase | 24HSP | A0A3B6KBM4 | Elongation factor Ts, mitochondrial |
| 24HSP | F1DKC1 | Catalase | 24HSP | A0A3B6N318 | Elongation factor Tu |
| 24HSP | A0A3B5XXK2 | CBM20 domain-containing protein | 24HSP | A0A3B6PNI9 | Elongation factor Tu |
| 24HSP | A0A3B6LF49 | CCD-B1 | 24HSP | A0A3B6SJX8 | Epimerase domain-containing protein |
| 24HSP | A0A3B6HUP2 | CCT-theta | 24HSP | A0A3B6H6X9 | Epimerase domain-containing protein |
| 24HSP | A0A3B6R9K7 | Cell cycle protein GpsB (Fragment) | 24HSP | A0A3B6LQ83 | Epimerase domain-containing protein |
| 24HSP | A0A3B5Y5Q7 | Cell death-related protein | 24HSP | A0A3B5ZVX1 | Epimerase domain-containing protein |
| 24HSP | A0A3B6JDS3 | CHCH domain-containing protein | 24HSP | R9W6A6 | ER molecular chaperone |
| 24HSP | A0A3B5XZR6 | Chitinase | 24HSP | A0A3B6TVN9 | Eukaryotic initiation factor 4A |
| 24HSP | A0A3B5ZVX5 | Chitinase | 24HSP | U5HTF2 | Eukaryotic translation initiation factor 3 subunit G |
| 24HSP | W5AY52 | Chlorophyll a-b binding protein, chloroplastic | 24HSP | A0A3B6C0K1 | Eukaryotic translation initiation factor 3 subunit M |
| 24HSP | A0A2X0S1W6 | Chloroplast ferredoxin-dependent glutamate synthase | 24HSP | Q3S4I1 | Eukaryotic translation initiation factor 5A |
| 24HSP | A0A3B5ZT18 | Chloroplast inner envelope protein, putative, expressed | 24HSP | Q03387 | Eukaryotic translation initiation factor isoform 4G-1 |
| 24HSP | A0A3B6JMF7 | Chorismate synthase | 24HSP | A0A3B6RAC4 | Expressed protein |
| 24HSP | A0A3B6LU21 | Citrate synthase | 24HSP | A0A3B6JIF5 | Expressed protein |
| 24HSP | A0A3B6PJY2 | Citrate synthase | 24HSP | A0A3B6RKP0 | Far upstream element-binding protein 3 |
| 24HSP | A0A3B6KGD4 | Clathrin heavy chain | 24HSP | A0A3B6QMN1 | Fe2OG dioxygenase domain-containing protein |
| 24HSP | A0A3B6UAW0 | Clathrin light chain | 24HSP | A0A3B6MQZ1 | Fe2OG dioxygenase domain-containing protein |
| 24HSP | A0A3B6U6K4 | Clp R domain-containing protein | 24HSP | A0A3B6PQS3 | Ferredoxin-nitrite reductase |
| 24HSP | A0A3B6MVC4 | Clp R domain-containing protein | 24HSP | W5IA32 | Formate dehydrogenase, mitochondrial |
| 24HSP | A0A1D6S634 | CMP/dCMP-type deaminase domain-containing protein | 24HSP | A0A3B6PQR7 | Formate dehydrogenase, mitochondrial |
| 24HSP | A0A3B6JR58 | Cold acclimation protein WCOR615 | 24HSP | W5FL09 | Fructose-bisphosphate aldolase |
| 24HSP | A0A3B6IWH4 | Cold acclimation protein WCOR615 | 24HSP | A0A3B6FHK1 | Fructose-bisphosphate aldolase |
| 24HSP | S6AWC2 | Cold induced 16 | 24HSP | A0A3B6NP66 | Fumarylacetoacetase |
| 24HSP | W5D5R6 | Cold induced protein | 24HSP | A0A3B6QEH8 | Gamma hydroxybutyrate dehydrogenase-like protein |
| 24HSP | A0A3B6EFA0 | Cold induced protein | 24HSP | A0A3B6C5X5 | Geranylgeranyl pyrophosphate synthase, chloroplastic |
| 24HSP | A0A3B6B480 | Cold-responsive protein WCOR14 | 24HSP | A0A3B6NUI6 | Geranylgeranyl reductase |
| 24HSP | A0A3B5ZS22 | Cyanate hydratase | 24HSP | A0A3B6D692 | Germin-like protein |
| 24HSP | A0A3B6DDR9 | Cysteine proteinase 1 | 24HSP | A0A3B5XXL3 | Gln-synt_C domain-containing protein |
| 24HSP | A0A3B6KLP1 | Cysteine synthase | 24HSP | A0A3B5Y6Y9 | Glucan endo-1,3-beta-D-glucosidase |
| 24HSP | A0A3B6C6U3 | Cytochrome b-c1 complex subunit Rieske, mitochondrial | 24HSP | A0A3B6EUA2 | Glucan endo-1,3-beta-glucosidase GI |
| 24HSP | P60162 | Cytochrome b6 | 24HSP | D8L9Q2 | Glucan endo-1,3-beta-glucosidase GII, putative, expressed |
| 24HSP | Q7X9A6 | Cytochrome b6-f complex iron-sulfur subunit, chloroplastic | 24HSP | A0A3B6H5C6 | Glucan endo-1,3-beta-glucosidase GII, putative, expressed |
| 24HSP | A0A3B6PSA8 | CYTOSOL_AP domain-containing protein | 24HSP | A0A3B6LW58 | Glucose-1-phosphate adenylyltransferase |

| Category | Accession | Description | Category | Accession | Description |
| --- | --- | --- | --- | --- | --- |
| 24HSP | A0A3B6QHK3 | Glucose-6-phosphate 1-dehydrogenase | 24HSP | W5I1R7 | KH type-2 domain-containing protein |
| 24HSP | A0A3B6MTB1 | Glucose-6-phosphate isomerase | 24HSP | W5HY47 | KOW domain-containing protein |
| 24HSP | A0A3B5XU98 | Glucose-6-phosphate isomerase | 24HSP | A0A3B6KEY6 | L-ascorbate peroxidase |
| 24HSP | A0A3B6SIC2 | Glutamate-1-semialdehyde 2,1-aminomutase | 24HSP | A0A3B6PTB4 | L-ascorbate peroxidase |
| 24HSP | A0A3B6GNX8 | Glutaminyl-tRNA synthetase | 24HSP | A0A3B6NXB6 | L-ascorbate peroxidase |
| 24HSP | A0A3B6EFJ3 | Glutaredoxin-dependent peroxiredoxin | 24HSP | A0A3B6JIA4 | Lactamase_B domain-containing protein |
| 24HSP | A0A3B6QI62 | Glutathione peroxidase | 24HSP | A0A3B5ZQU4 | Lipase_3 domain-containing protein |
| 24HSP | Q8RW01 | Glutathione transferase | 24HSP | A0A3B6H278 | Lipase_3 domain-containing protein |
| 24HSP | Q8RW00 | Glutathione transferase | 24HSP | A0A3B6MY02 | Lipoxygenase |
| 24HSP | A0A3B6ENE2 | Glutathione transferase | 24HSP | A0A3B6PLP6 | Lipoxygenase |
| 24HSP | A0A3B6EPN2 | Glutathione transferase | 24HSP | A0A3B6MID4 | Lipoxygenase |
| 24HSP | A0A3B6RAZ3 | Glutathione transferase | 24HSP | A0A3B6H269 | LRRNT_2 domain-containing protein |
| 24HSP | A0A3B6I5M9 | Glutathione transferase | 24HSP | A0A3B6PP63 | M16C_associated domain-containing protein |
| 24HSP | A0A3B6RKE1 | Glyceraldehyde-3-phosphate dehydrogenase | 24HSP | A0A3B6QKG3 | M16C_associated domain-containing protein |
| 24HSP | A0A3B6ASE9 | Glycerol-3-phosphate dehydrogenase | 24HSP | W5FIX9 | Macrophage migration inhibitory factor family protein, expressed |
| 24HSP | A0A3B6T784 | Glycine-tRNA ligase | 24HSP | A0A3B6AUK1 | Magnesium chelatase |
| 24HSP | A0A3B6NKE4 | Glycine-rich protein 2 | 24HSP | A0A3B6EGW9 | Magnesium-protoporphyrin IX monomethyl ester (oxidative) cyclase |
| 24HSP | A0A3B6IKX2 | Glycine-rich protein | 24HSP | A0A3B6U6N7 | Malate dehydrogenase (NADP^+^) |
| 24HSP | A0A3B6I2T0 | glycine-rich RNA-binding protein RZ1A | 24HSP | A0A3B6EE64 | MAR-binding filament-like protein 1 |
| 24HSP | A0A3B6DHK2 | Glycosyltransferase | 24HSP | A0A3B6IVP5 | Methionyl-tRNA synthetase |
| 24HSP | A0A3B6U377 | Glycosyltransferase | 24HSP | A0A3B6JFM6 | Methylthioribose-1-phosphate isomerase |
| 24HSP | A0A3B6HPL1 | Glycosyltransferase | 24HSP | A0A3B6QAJ9 | Mitochondrial ATP synthase |
| 24HSP | A0A3B6RMH1 | Glyoxalase I | 24HSP | W5CX03 | Mitochondrial glycoprotein family protein |
| 24HSP | A0A3B6LVU2 | Glyoxalase I | 24HSP | A0A3B6MPR0 | Mitochondrial outer membrane porin |
| 24HSP | A0A3B5ZRK2 | Glyoxalase I | 24HSP | A0A3B6JNT4 | Mitochondrial outer membrane protein porin of 34 kDa |
| 24HSP | A0A3B6MXQ6 | GOLD domain-containing protein | 24HSP | A0A3B6PQ00 | Mitochondrial phosphate transporter |
| 24HSP | A7VL25 | Group3 late embryogenesis abundant protein | 24HSP | A0A3B6C558 | Mitochondrial protein YMF19 |
| 24HSP | A0A3B6LF64 | GSDH domain-containing protein | 24HSP | Q7XY22 | Mitochondrial pyruvate carrier |
| 24HSP | W5B016 | GTP-binding protein Rab6 | 24HSP | A0A3B6IVK1 | Mitochondrial-processing peptidase beta subunit |
| 24HSP | B2ZGG5 | GTPase SAR1 | 24HSP | A0A3B6JNG4 | Mitochondrial-processing peptidase beta subunit |
| 24HSP | A0A3B6CGQ1 | H0112G12.10 protein | 24HSP | K4HRS2 | Monodehydroascorbate reductase 4 |
| 24HSP | A0A3B6B901 | H0306F12.7 protein | 24HSP | U5MY58 | Monodehydroasorbate reductase |
| 24HSP | A0A3B6TYS2 | HATPase_c domain-containing protein | 24HSP | A0A3B5YW44 | Monothiol glutaredoxin-S11 |
| 24HSP | A0A3B5YVQ2 | HD domain-containing protein | 24HSP | A0A3B6TJ25 | MtN19-like protein |
| 24HSP | W5GLX4 | Heat shock cognate 70 kDa protein 1 | 24HSP | C1K737 | Multiprotein bridging factor 1 |
| 24HSP | A0A3B6TN74 | Heme oxygenase 1 | 24HSP | A0A3B6PLA1 | Myb-like domain-containing protein |
| 24HSP | A0A3B6DL15 | Hexosyltransferase | 24HSP | M9XEK1 | Myo-inositol 1-phosphate synthase |
| 24HSP | A0A3B6HSI1 | Hexosyltransferase | 24HSP | A0A3B6SD90 | Myosin heavy chain-related |
| 24HSP | W5C7Q2 | HIG1 domain-containing protein | 24HSP | W5DZS4 | NAD(P)-bd_dom domain-containing protein |
| 24HSP | A0A3B5ZRV5 | Histidine-tRNA ligase | 24HSP | A0A3B6GV04 | NAD(P)-bd_dom domain-containing protein |
| 24HSP | A0A3B6CFF4 | Histidine-tRNA ligase | 24HSP | A0A3B5XTZ8 | NAD(P)-bd_dom domain-containing protein |
| 24HSP | W5CXE7 | Hydrogen-transporting ATP synthase, rotational mechanism | 24HSP | A0A1D6CRV7 | NADH dehydrogenase [ubiquinone] 1 beta subcomplex subunit 2 |
| 24HSP | A0A341WSR4 | Hydroxypyruvate reductase | 24HSP | A0A3B6C7H1 | NADH dehydrogenase [ubiquinone] flavoprotein 1, mitochondrial |
| 24HSP | B6D9L4 | Hypersensitive induced response protein 3 | 24HSP | A0A3B6JFJ4 | NADH dehydrogenase [ubiquinone] iron-sulfur protein 1, mitochondrial |
| 24HSP | A5HE90 | Hypersensitive response protein | 24HSP | A0A3B5ZVK4 | NADH-ubiquinone oxidoreductase, putative, expressed |
| 24HSP | A0A3B5ZWW5 | Inhibitor I9 domain-containing protein | 24HSP | A0A3B6QE21 | NADPH-dependent pterin aldehyde reductase |
| 24HSP | A0A3B5ZTT5 | Inositol-tetrakisphosphate 1-kinase | 24HSP | A0A1D5UG74 | Naringenin, 2-oxoglutarate 3-dioxygenase |
| 24HSP | A0A3B6CG72 | Intracellular protease 1 | 24HSP | A0A3B6HYW3 | Nascent polypeptide-associated complex subunit beta |
| 24HSP | A0A3B6QHG5 | Iso_dh domain-containing protein | 24HSP | A0A3B5XVW0 | Nfu_N domain-containing protein |
| 24HSP | A0A3B5ZS82 | J domain-containing protein | 24HSP | A0A3B6QJ10 | Nitrite reductase |

| Category | Accession | Description | Category | Accession | Description |
| --- | --- | --- | --- | --- | --- |
| 24HSP | A0A3B5Y1E9 | NMO domain-containing protein | 24HSP | A0A3B6RJH1 | Polyadenylate-binding protein |
| 24HSP | A0A3B6RIZ4 | NmrA domain-containing protein | 24HSP | A0A3B6MNL5 | Polyadenylate-binding protein |
| 24HSP | A0A3B6PJ33 | NPL4-like protein | 24HSP | Q41523 | PR17d |
| 24HSP | W5CT58 | Nucleic acid-binding protein | 24HSP | W5B748 | Pre-mRNA cleavage factor Im 25 kDa subunit |
| 24HSP | A0A3B6JDN5 | O-methyltransferase ZRP4 | 24HSP | A0A3B6N207 | Pre-mRNA processing factor |
| 24HSP | A0A1D6DIM7 | Obg-like ATPase 1 | 24HSP | A0A3B6LQR8 | Probable 6-phosphogluconolactonase |
| 24HSP | A0A3B6H6A6 | Orotate phosphoribosyltransferase | 24HSP | A0A3B6TMV0 | Probable alanine-tRNA ligase, chloroplastic |
| 24HSP | A0A3B6CB33 | OSJNBb0011N17.8 protein | 24HSP | A0A3B6SCX2 | Probable uridine nucleosidase 1 |
| 24HSP | A0A1D5UJQ4 | OSJNBb0039L24.13 protein | 24HSP | A0A1D5YY58 | Proteasome subunit alpha type |
| 24HSP | A0A3B6B328 | OSJNBb0039L24.13 protein | 24HSP | A0A3B6JNC2 | Proteasome subunit alpha type |
| 24HSP | A0A3B6AQN0 | Oxidored_FMN domain-containing protein | 24HSP | A0A1D6CXF2 | Proteasome subunit alpha type |
| 24HSP | W5B9L0 | Oxygen evolving enhancer protein | 24HSP | A0A3B6SFC8 | Protein cbbY |
| 24HSP | A0A3B6HRY1 | Oxysterol-binding protein 8 | 24HSP | A0A3B6GVP6 | Protein CHLOROPLAST ENHANCING STRESS TOLERANCE, chloroplastic |
| 24HSP | A0A3B6MVT1 | Pantothenate kinase 2 | 24HSP | Q93XQ8 | Protein disulfide-isomerase |
| 24HSP | A0A3B6DHZ2 | PAP_fibrillin domain-containing protein | 24HSP | A0A3B6HYX1 | Protein disulfide-isomerase |
| 24HSP | A0A3B6CE00 | PAP_fibrillin domain-containing protein | 24HSP | A0A3B6KGR6 | Protein disulfide-isomerase |
| 24HSP | A0A3B5Z6T3 | Pathogen-related protein | 24HSP | A0A3B6GXU5 | Protein GPR107 |
| 24HSP | C3UZE5 | Pathogenesis-related protein 1-1 | 24HSP | W5AY37 | Protein kinase domain-containing protein |
| 24HSP | A0A3B5XZZ1 | PCI domain-containing protein | 24HSP | A0A3B6LXX0 | Protein kinase domain-containing protein |
| 24HSP | A0A3B6N2H4 | PCI domain-containing protein | 24HSP | A0A3B6GZD6 | Protein kinase domain-containing protein |
| 24HSP | A0A3B6RNP1 | Peptidase A1 domain-containing protein | 24HSP | A0A3B6INI6 | Protein kinase domain-containing protein |
| 24HSP | A0A3B5YZ06 | Peptidase_M24 domain-containing protein | 24HSP | A0A1D6BIN9 | Protein mago nashi-like protein |
| 24HSP | C0J025 | Peptidyl-prolyl cis-trans isomerase | 24HSP | W5CUJ0 | Protein phosphatase |
| 24HSP | A0A3B5XSZ5 | Peptidyl-prolyl cis-trans isomerase | 24HSP | A0A3B6GQJ1 | Protein translocase subunit SecA |
| 24HSP | A0A3B6BZV6 | Peptidyl-prolyl cis-trans isomerase | 24HSP | A0A3B6NWI0 | Protein TSS |
| 24HSP | C6ETA5 | Peroxidase | 24HSP | A0A3B5YXL9 | Protein-methionine-S-oxide reductase |
| 24HSP | Q43212 | Peroxidase | 24HSP | A0A3B6GSD2 | Protein-serine/threonine phosphatase |
| 24HSP | A0A3B6CH98 | Peroxidase | 24HSP | W5B1P6 | Protein-serine/threonine phosphatase |
| 24HSP | A0A3B6B887 | Peroxidase | 24HSP | A0A3B6TIA2 | PsbP domain-containing protein |
| 24HSP | A0A3B6DPL3 | Peroxidase | 24HSP | A0A3B6TYJ2 | PsbP domain-containing protein |
| 24HSP | A0A3B5ZQ45 | Peroxidase | 24HSP | A0A3B6ESP8 | Pseudouridine-5'-monophosphatase |
| 24HSP | A0A3B6H3P5 | Peroxidase | 24HSP | D3KVP3 | Purple acid phosphatase |
| 24HSP | A0A3B6PH99 | Peroxidase | 24HSP | A0A3B6NNX5 | Putative 4-coumarate-CoA ligase 3 |
| 24HSP | Q5S1S6 | Peroxiredoxin Q, chloroplastic | 24HSP | A0A077RX76 | Putative acyl transferase 3 |
| 24HSP | A0A3B6QF91 | Phenylalanine ammonia-lyase | 24HSP | W5D2A3 | Putative mitochondrial import receptor subunit TOM20 |
| 24HSP | A0A3B6IK29 | Phosphoglucomutase (alpha-D-glucose-1,6-bisphosphate-dependent) | 24HSP | W5D122 | Putative oxidoreductase GLYR1 |
| 24HSP | A0A3B5Z298 | Phosphoglycerate kinase | 24HSP | W5C4P1 | Putative oxygen-evolving complex |
| 24HSP | A0A3B6NNE0 | Phosphoglycerate kinase | 24HSP | A0A3B6IU00 | Putative rhamnose biosynthetic enzyme 1 |
| 24HSP | W5D4Q6 | Phospholipase D | 24HSP | E6Y0T1 | Putative SNAP receptor protein |
| 24HSP | P26302 | Phosphoribulokinase, chloroplastic | 24HSP | A0A1D5ZBB4 | Putative vesicle-associated membrane protein 726 |
| 24HSP | A0A3B6HRZ3 | Phosphoserine aminotransferase | 24HSP | A0A3B6IRV7 | Putative Xaa-Pro aminopeptidase 2 |
| 24HSP | A0A3B5ZSS7 | Phosphotransferase | 24HSP | A0A3B6H611 | Pyridoxal phosphate homeostasis protein |
| 24HSP | A0A3B6LPN8 | Photolyase/cryptochrome alpha/beta domain-containing protein | 24HSP | A0A1D6D1Q3 | Pyrophosphate-fructose 6-phosphate 1-phosphotransferase subunit beta |
| 24HSP | A0A3B6QMM8 | Photosystem II 10 kDa polypeptide, chloroplastic | 24HSP | W5C673 | Pyrrolidone-carboxylate peptidase |
| 24HSP | Q36814 | Photosystem II D2 protein | 24HSP | W5C3E3 | Pyruvate dehydrogenase E1 component subunit alpha |
| 24HSP | A0A3B6GVK7 | PITH domain-containing protein | 24HSP | A0A1D5WW05 | Pyruvate kinase |
| 24HSP | A0A3B6TEG7 | PKS_ER domain-containing protein | 24HSP | W4ZLP9 | RanBD1 domain-containing protein |
| 24HSP | A0A3B6ED41 | PKS_ER domain-containing protein | 24HSP | A0A3B5ZUU7 | RanBD1 domain-containing protein |
| 24HSP | A0A3B6KID6 | PKS_ER domain-containing protein | 24HSP | A0A1D5W7M9 | Ras-related protein RIC1 |
| 24HSP | A0A3B6NMG8 | PKS_ER domain-containing protein | 24HSP | A0A3B5ZT57 | Remorin, C-terminal region family protein, expressed |

| Category | Accession | Description | Category | Accession | Description |
| --- | --- | --- | --- | --- | --- |
| 24HSP | A0A3B6SBT2 | Rhodanese domain-containing protein | 24HSP | Q8S9H0 | Temperature stress-induced lipocalin |
| 24HSP | A0A3B6QHC8 | Rhodanese domain-containing protein | 24HSP | A0A3B6CEX1 | Tetratricopeptide repeat (TPR)-like superfamily protein |
| 24HSP | A0A3B6EM75 | Rhodanese domain-containing protein | 24HSP | A0A3B6DB45 | Tetratricopeptide repeat containing protein |
| 24HSP | A0A3B6MQM1 | Rhodanese domain-containing protein | 24HSP | A0A3B6JDF8 | Thaumatin-like protein TLP8 |
| 24HSP | A0A3B6DD84 | Ribose-5-phosphate isomerase | 24HSP | A0A3B6JIP4 | Thioredoxin domain-containing protein |
| 24HSP | Q5I7L1 | Ribosomal protein L13a | 24HSP | A0A3B6DBB2 | Thioredoxin domain-containing protein |
| 24HSP | Q5I7K4 | Ribosomal protein L17 | 24HSP | A0A3B6C9S5 | Thioredoxin domain-containing protein |
| 24HSP | A0A3B5YZT7 | Ribosomal protein | 24HSP | O64394 | Thioredoxin H-type |
| 24HSP | A0A3B6PL02 | Ribosomal_L14e domain-containing protein | 24HSP | A0A1D5S567 | Thioredoxin |
| 24HSP | A0A0C4BIR6 | Ribosomal_L16 domain-containing protein | 24HSP | A0A3B5Z1P0 | Thioredoxin |
| 24HSP | A0A3B6DE43 | Ribosomal_L18_c domain-containing protein | 24HSP | A0A3B6GRB7 | Thylakoid lumenal 17.9 kDa protein, chloroplastic |
| 24HSP | W5BPV2 | Ribosomal_L18e/L15P domain-containing protein | 24HSP | A0A3B6N1I5 | TOG domain-containing protein |
| 24HSP | A0A3B6C571 | Ribosomal_L18e/L15P domain-containing protein | 24HSP | A0A3B6TS42 | Tr-type G domain-containing protein |
| 24HSP | W5ECL2 | Ribosomal_L2_C domain-containing protein | 24HSP | A0A1D5V3K8 | Trafficking protein particle complex subunit |
| 24HSP | A0A1D5UQZ5 | Ribosomal_L23eN domain-containing protein | 24HSP | A0A3B6H0W1 | Transaldolase |
| 24HSP | W5DEJ5 | Ribosomal_L28e domain-containing protein | 24HSP | A0A3B6ASP6 | Transket_pyr domain-containing protein |
| 24HSP | A0A3B5ZZN9 | Ribosomal_L28e domain-containing protein | 24HSP | A0A3B6C6P4 | Transket_pyr domain-containing protein |
| 24HSP | W5I3L0 | Ribosomal_L7Ae domain-containing protein | 24HSP | A0A3B6TID0 | Transketolase |
| 24HSP | W5BFB7 | Ribosomal_S17_N domain-containing protein | 24HSP | A0A3B6ELC9 | TRASH domain-containing protein |
| 24HSP | A0A3B6NTA2 | RNA-binding Musashi-2-like protein | 24HSP | A0A3B6CEW9 | Ubiquitin carboxyl-terminal hydrolase 22 |
| 24HSP | A0A3B6HUD4 | RRM domain-containing protein | 24HSP | A0A3B6NQ23 | Ubiquitin carboxyl-terminal hydrolase |
| 24HSP | A0A3B6PV81 | rRNA 2'-O-methyltransferase fibrillarin 1 | 24HSP | W5ADS2 | Ubiquitin |
| 24HSP | A0A3B6RB14 | RuBisCO large subunit-binding protein subunit beta, chloroplastic | 24HSP | A0A3B6JFX9 | UDP-glucose 6-dehydrogenase |
| 24HSP | A0A3B6QKH8 | S-(hydroxymethyl) glutathione dehydrogenase | 24HSP | A0A3B6C053 | UDP-glucosyltransferase |
| 24HSP | A0A1D6B308 | S-adenosylmethionine synthase | 24HSP | A0A3B6EQU7 | Uricase |
| 24HSP | A0A3B6PFM5 | S1 motif domain-containing protein | 24HSP | A0A3B6QH30 | Usp domain-containing protein |
| 24HSP | W5ATK7 | Salt tolerant protein | 24HSP | A0A3B6PQQ9 | Usp domain-containing protein |
| 24HSP | A0A3B6KF02 | SCP domain-containing protein | 24HSP | A0A1D6S518 | UTP-glucose-1-phosphate uridylyltransferase |
| 24HSP | A0A3B6SGZ1 | Sec-independent translocase protein tatA/E-like protein | 24HSP | W5BT22 | UV-B-induced protein chloroplastic |
| 24HSP | A0A3B6ELV2 | Secretory carrier-associated membrane protein | 24HSP | A0A3B6JM59 | V-type proton ATPase subunit a |
| 24HSP | P08819 | Serine carboxypeptidase 2 | 24HSP | A0A3B6JM94 | VPS9 domain-containing protein |
| 24HSP | A0A3B6CE87 | Serine hydroxymethyltransferase | 24HSP | A0A3B6TB53 | VWFA domain-containing protein |
| 24HSP | A0A3B6KJ32 | SHSP domain-containing protein | 24HSP | A0A341UGW3 | W2 domain-containing protein |
| 24HSP | W5HV90 | Signal recognition particle 9 kDa protein | 24HSP | Q8L5C6 | Xylanase inhibitor protein 1 |
| 24HSP | Q5I5K7 | Small GTP-binding protein | 24HSP | A0A3B6DJQ6 | Zinc finger CCCH domain-containing protein 31 |
| 24HSP | A0A3B6DFE2 | Small nuclear ribonucleoprotein-associated protein | 24HSP | A0A3B6TVQ6 | Zinc finger CCCH domain-containing protein 44 |
| 24HSP | W5D591 | Small ubiquitin-related modifier | 0HSP | A0A3B5Z4L3 | 1,2-dihydroxy-3-keto-5-methylthiopentene dioxygenase |
| 24HSP | A0A3B6C6E8 | SnRK1 gamma subunit | 0HSP | A0A1D5UUP9 | 10 kDa chaperonin |
| 24HSP | W5D4D4 | Sodium/calcium exchanger NCL1 | 0HSP | A0A3B6IPI3 | 2-carboxy-D-arabinitol-1-phosphatase |
| 24HSP | A0A3B6GX01 | SOUL heme-binding domain containing protein, expressed | 0HSP | A0A3B6KFM7 | 2-isopropylmalate synthase |
| 24HSP | A0A3B6HUD3 | SRP54 domain-containing protein | 0HSP | A0A3B6QHC3 | 2-oxoglutarate (2OG) and Fe(II)-dependent oxygenase superfamily protein |
| 24HSP | A0A3B6MMK9 | Stress-response A/B barrel domain-containing protein | 0HSP | A0A3B6B2A6 | 2-phytyl-1,4-beta-naphthoquinone methyltransferase, chloroplastic |
| 24HSP | A0A3B6C2L2 | Stress-response A/B barrel domain-containing protein UP3 | 0HSP | A0A1D6D1N1 | 20 kDa chaperonin, chloroplastic |
| 24HSP | A0A3B6LU07 | Succinate dehydrogenase [ubiquinone] flavoprotein subunit, mitochondrial | 0HSP | A0A3B6RAV4 | 20 kDa chaperonin, chloroplastic |
| 24HSP | A0A3B6RH41 | Succinate dehydrogenase [ubiquinone] iron-sulfur subunit, mitochondrial | 0HSP | A0A3B6DIW5 | 26S proteasome non-ATPase regulatory subunit 1 homolog |
| 24HSP | W5C4B7 | Succinate-CoA ligase [ADP-forming] subunit alpha, mitochondrial | 0HSP | A0A3B6TM54 | 3-hydroxyisobutyryl-CoA hydrolase-like protein 3 mitochondrial |
| 24HSP | A0A1D6B2M0 | Succinate-CoA ligase [ADP-forming] subunit beta, mitochondrial | 0HSP | W5EIQ2 | 3-ketoacyl-CoA synthase |
| 24HSP | A0A3B6TMN0 | Sucrose synthase | 0HSP | P60577 | 30S ribosomal protein S19, chloroplastic |
| 24HSP | A0A1D6CXJ5 | T-complex protein 1 subunit delta | 0HSP | Q95H49 | 30S ribosomal protein S3, chloroplastic |
| 24HSP | A0A3B6SMZ6 | T-complex protein 1 subunit gamma | 0HSP | W5FPA7 | 30S ribosomal protein S4, chloroplastic |

| Category | Accession | Description | Category | Accession | Description |
| --- | --- | --- | --- | --- | --- |
| 0HSP | W5C5U5 | 30S ribosomal protein S4, chloroplastic | 0HSP | A0A3B6SF84 | Alternative NAD(P)H-ubiquinone oxidoreductase C1, chloroplastic/mitochondrial |
| 0HSP | W5D4Z8 | 4-(cytidine-5'-diphospho)-2-C-methyl-D-erythritol kinase | 0HSP | A0A3B6H2I8 | Amidophosphoribosyltransferase |
| 0HSP | A0A3B6AU50 | 4-alpha-glucanotransferase | 0HSP | W5AUD8 | Aminotran_1_2 domain-containing protein |
| 0HSP | A0A3B6HW46 | 4-hydroxy-3-methylbut-2-enyl diphosphate reductase | 0HSP | A0A3B6LVK5 | Anthranilate synthase |
| 0HSP | A0A3B6JDC9 | 4-hydroxy-3-methylbut-2-enyl diphosphate reductase | 0HSP | A0A1D6AM47 | Aquaporin |
| 0HSP | Q1XIR9 | 4-hydroxy-7-methoxy-3-oxo-3,4-dihydro-2H-1,4-benzoxazin-2-yl glucoside beta-D-glucosidase 1a, chloroplastic | 0HSP | A0A3B6HQN3 | Argininosuccinate lyase |
| 0HSP | A0A3B6LP26 | 4-hydroxy-7-methoxy-3-oxo-3,4-dihydro-2H-1,4-benzoxazin-2-yl glucosidebeta-D-glucosidase | 0HSP | A0A3B6LSK6 | Arogenate dehydratase |
| 0HSP | W5FV77 | 40S ribosomal protein S23 | 0HSP | A0A3B5Z514 | Asparagine synthetase [glutamine-hydrolyzing] |
| 0HSP | Q5I7K3 | 40S ribosomal protein S29 | 0HSP | A0A3B5XWA7 | Asparagine-tRNA ligase |
| 0HSP | A0A1D5SLI4 | 40S ribosomal protein S4 | 0HSP | A0A3B6NJ31 | Aspartate aminotransferase |
| 0HSP | W5C8N6 | 40S ribosomal protein S6 | 0HSP | A0A3B6MRS1 | Aspartokinase |
| 0HSP | A0A3B6HQ62 | 40S ribosomal protein SA | 0HSP | A0A3B6E9Q1 | Aspergillus nuclease S(1) |
| 0HSP | A0A3B6C534 | 40S ribosomal protein SA | 0HSP | Q332R4 | ATP synthase subunit alpha |
| 0HSP | Q95H48 | 50S ribosomal protein L22, chloroplastic | 0HSP | P12112 | ATP synthase subunit alpha, chloroplastic |
| 0HSP | W5CQ20 | 50S ribosomal protein L31 | 0HSP | A0A3B5Y5K9 | ATP synthase subunit beta |
| 0HSP | W5FSM8 | 60S ribosomal protein L17-1 | 0HSP | P20858 | ATP synthase subunit beta, chloroplastic |
| 0HSP | A0A3B6QGW3 | 60S ribosomal protein L6 | 0HSP | A0A3B6EKW3 | ATP synthase subunit epsilon, mitochondrial |
| 0HSP | A0A3B6PN63 | 60S ribosomal protein L6 | 0HSP | A0A3B5ZT15 | ATP synthase subunit gamma |
| 0HSP | W5GVR2 | 60S ribosomal protein L6 | 0HSP | A0A3B5ZTY8 | ATP-dependent Clp protease proteolytic subunit |
| 0HSP | A0A077RSZ8 | 8-amino-7-oxononanoate synthase | 0HSP | A0A3B6GUS2 | ATP-dependent Clp protease proteolytic subunit |
| 0HSP | A0A077RXS4 | AAA domain-containing protein | 0HSP | A0A3B6B6N9 | ATPase_AAA_core domain-containing protein |
| 0HSP | A0A3B6A2N9 | AAA domain-containing protein | 0HSP | A0A3B6AXC6 | Beta-amylase |
| 0HSP | A0A3B6RMS3 | AAA domain-containing protein | 0HSP | A0A3B6RQL9 | BnaC05g49910D protein |
| 0HSP | A0A3B6NSE6 | AAA domain-containing protein | 0HSP | A0A3B6TM09 | Bsd2 |
| 0HSP | A0A3B6JQ40 | AAI domain-containing protein | 0HSP | A0A3B6MVC8 | C2 NT-type domain-containing protein |
| 0HSP | A0A3B6ISQ8 | ABC transporter domain-containing protein | 0HSP | A0A3B6DB95 | Calcium-dependent protein kinase |
| 0HSP | A0A3B6GZI6 | ABC transporter domain-containing protein | 0HSP | A0A3B6GT39 | Carbonic anhydrase |
| 0HSP | A0A3B6H1B6 | ABC transporter subunit-like | 0HSP | A0A3B6FP56 | Carboxypeptidase |
| 0HSP | A0A3B6HYQ0 | Abcissic acid stress ripening | 0HSP | A0A3B6RB99 | CBM20 domain-containing protein |
| 0HSP | A0A3B6DDG1 | Abscisic stress-ripening protein 2 | 0HSP | A0A3B5XXK2 | CBM20 domain-containing protein |
| 0HSP | A0A1D6D5I2 | ACB domain-containing protein | 0HSP | A0A3B6RJQ9 | CBS domain-containing protein-like |
| 0HSP | B2ZGL4 | Acetyl-CoA carboxylase | 0HSP | A0A3B6HUP2 | CCT-theta |
| 0HSP | A0A3B6PQ12 | Aconitase_C domain-containing protein | 0HSP | A0A341YEW4 | Chlorophyll a-b binding protein, chloroplastic |
| 0HSP | A0A3B6KT79 | Aconitate hydratase | 0HSP | A0A2X0S1W6 | Chloroplast ferredoxin-dependent glutamate synthase |
| 0HSP | A0A3B6FVN1 | Acyl-[acyl-carrier-protein] desaturase 2, chloroplastic | 0HSP | A0A3B5ZT18 | Chloroplast inner envelope protein, putative, expressed |
| 0HSP | W5ALC0 | Acyl-coenzyme A oxidase 4, peroxisomal | 0HSP | A0A3B5XZJ7 | Chloroplast inner envelope protein, putative, expressed |
| 0HSP | A0A3B6PM63 | Adenosine kinase | 0HSP | A0A3B5YVW2 | Chloroplast inner envelope protein, putative, expressed |
| 0HSP | A0A3B6B781 | Adenosylhomocysteinase | 0HSP | A0A3B6PJY2 | Citrate synthase |
| 0HSP | A0A3B6TT87 | Adenylate kinase | 0HSP | A0A3B6U6K4 | Clp R domain-containing protein |
| 0HSP | A0A3B6I0G2 | Adenylyl cyclase-associated protein | 0HSP | A0A3B5XY59 | Coatomer subunit gamma |
| 0HSP | Q76ME3 | ADP-ribosylation factor | 0HSP | A0A3B6IWH4 | Cold acclimation protein WCOR615 |
| 0HSP | A0A3B6UBC9 | Agglutinin domain-containing protein | 0HSP | A0A3B6BXP4 | CP12 domain-containing protein |
| 0HSP | A0A3B6LRD0 | Alba domain-containing protein | 0HSP | A0A341YVV0 | CREG1 protein |
| 0HSP | A0A3B6TH43 | Alba domain-containing protein | 0HSP | A0A3B6RFV2 | CS domain-containing protein |
| 0HSP | A0A3B5ZYK2 | Aldo_ket_red domain-containing protein | 0HSP | A0A3B6LHY5 | CTP synthase |
| 0HSP | A0A3B6C663 | Aldo_ket_red domain-containing protein | 0HSP | A0A3B6JG56 | Cysteine desulfurase |
| 0HSP | D8L9G6 | Alpha-1,4 glucan phosphorylase | 0HSP | A0A3B6KP84 | Cytochrome b-c1 complex subunit 7 |
| 0HSP | A0A3B6LTI6 | Alpha-1,4 glucan phosphorylase | 0HSP | Q7X9A6 | Cytochrome b6-f complex iron-sulfur subunit, chloroplastic |
| 0HSP | A0A3B6MN38 | Alpha-D-phosphohexomutase superfamily | 0HSP | A0A3B6SIV9 | Cytochrome c domain-containing protein |
| 0HSP | A0A3B6RBU0 | Alternative NAD(P)H-ubiquinone oxidoreductase C1, chloroplastic/mitochondrial | 0HSP | A0A1D5YVB5 | Cytochrome c oxidase subunit 5C |

| Category | Accession | Description | Category | Accession | Description |
| --- | --- | --- | --- | --- | --- |
| 0HSP | A0A3B6PSA8 | CYTOSOL_AP domain-containing protein | 0HSP | A0A3B6SIC2 | Glutamate-1-semialdehyde 2,1-aminomutase |
| 0HSP | W5CY74 | DEAD-box ATP-dependent RNA helicase 25 | 0HSP | A0A3B6QCY2 | Glutamyl-tRNA synthetase |
| 0HSP | A0A3B6SGQ9 | Defective in cullin neddylation protein | 0HSP | A0A3B6QDQ4 | Glutamyl-tRNA (Gln) amidotransferase subunit B, chloroplastic/mitochondrial |
| 0HSP | A0A3B6RG17 | Dehydroascorbate reductase | 0HSP | A0A3B6EFJ3 | Glutaredoxin-dependent peroxiredoxin |
| 0HSP | A0A3B6MVZ0 | DHQ_synthase domain-containing protein | 0HSP | A0A3B6JNZ9 | Glutathione S-transferase |
| 0HSP | A0A3B6PS22 | diacylglycerol kinase | 0HSP | A0A3B6EPN2 | Glutathione transferase |
| 0HSP | A0A3B6TJ13 | Diadenosine tetraphosphate synthetase | 0HSP | A0A3B6ASE9 | Glycerol-3-phosphate dehydrogenase |
| 0HSP | A0A3B6AZN1 | Diadenosine tetraphosphate synthetase | 0HSP | A0A3B5ZWW3 | Glyco_hydro_18 domain-containing protein |
| 0HSP | A0A3B6LWF0 | Dihydrolipoamide acetyltransferase component of pyruvate dehydrogenase complex | 0HSP | D5MTE0 | Glycosyltransferase |
| 0HSP | A0A3B6N0L8 | Dihydrolipoamide acetyltransferase component of pyruvate dehydrogenase complex | 0HSP | A0A3B6KUM9 | Glycosyltransferase |
| 0HSP | A0A3B6DN56 | Dihydroorotate dehydrogenase (quinone), mitochondrial | 0HSP | A0A3B6BZ82 | Glycosyltransferase |
| 0HSP | A0A3B6TJJ8 | Dihydroxy-acid dehydratase | 0HSP | A0A3B6KK51 | Glycosyltransferase |
| 0HSP | A0A3B6HUF8 | Dirigent protein | 0HSP | A0A3B6EHB2 | Glyoxylate reductase |
| 0HSP | A0A3B6QFT8 | DNA damage-inducible protein 1 | 0HSP | A0A3B6AYW6 | GrpE protein homolog |
| 0HSP | A0A3B6JMS5 | Dolichyl-diphosphooligosaccharide-protein glycosyltransferase subunit 1 | 0HSP | A0A3B6TWH8 | GTP-binding nuclear protein Ran-3 |
| 0HSP | A0A3B6LW93 | DPP6 N-terminal domain-like protein | 0HSP | A0A3B6SP42 | H^+^-exporting diphosphatase |
| 0HSP | A0A3B6GTT8 | DREPP4 protein | 0HSP | A0A3B6CGQ1 | H0112G12.10 protein |
| 0HSP | A0A3B6TP00 | DUF953 domain-containing protein | 0HSP | A0A3B6KR72 | H15 domain-containing protein |
| 0HSP | A0A3B5ZNF9 | E3 UFM1-protein ligase 1 homolog | 0HSP | A0A3B6MXZ6 | H15 domain-containing protein |
| 0HSP | A0A3B5XVE8 | E3 UFM1-protein ligase 1-like protein | 0HSP | A0A3B6KJX5 | HATPase_c domain-containing protein |
| 0HSP | A0A3B6KBM4 | Elongation factor Ts, mitochondrial | 0HSP | A0A3B6MT71 | HATPase_c domain-containing protein |
| 0HSP | A0A3B6N318 | Elongation factor Tu | 0HSP | A0A3B6GZK9 | Heat shock 70 kDa protein, mitochondrial |
| 0HSP | A0A3B6MND7 | Endopolygalacturonase AN8327 (Fragment) | 0HSP | A0A3B6TN74 | Heme oxygenase 1 |
| 0HSP | A0A3B6LQ83 | Epimerase domain-containing protein | 0HSP | A0A3B6SJ32 | Heme-binding-like protein At3g10130, chloroplastic |
| 0HSP | A0A3B5ZVX1 | Epimerase domain-containing protein | 0HSP | A0A3B6CFF4 | Histidine-tRNA ligase |
| 0HSP | A0A3B6TVN9 | Eukaryotic initiation factor 4A | 0HSP | W5GDR6 | Histone deacetylase complex subunit SAP18 |
| 0HSP | A0A3B6A138 | Eukaryotic translation initiation factor 3 subunit D | 0HSP | A0A3B6HTP8 | Histone domain-containing protein |
| 0HSP | U5HTF2 | Eukaryotic translation initiation factor 3 subunit G | 0HSP | A0A3B6H4D4 | Histone H2A |
| 0HSP | Q3S4I1 | Eukaryotic translation initiation factor 5A | 0HSP | A0A3B6DHS2 | HMA domain-containing protein |
| 0HSP | Q03389 | Eukaryotic translation initiation factor isoform 4E-2 | 0HSP | A0A3B6LI13 | Homoserine dehydrogenase |
| 0HSP | Q03387 | Eukaryotic translation initiation factor isoform 4G-1 | 0HSP | A0A3B6LJD2 | Hypothetical_protein |
| 0HSP | A0A3B6KQ25 | Expressed protein | 0HSP | A0A3B6B107 | Indole-3-glycerol-phosphate synthase |
| 0HSP | A0A3B6LIF0 | Expressed protein | 0HSP | A0A3B6PT23 | Isoleucyl-tRNA synthetase |
| 0HSP | A0A3B6ET26 | Expressed protein | 0HSP | A0A3B6LWK1 | J domain-containing protein |
| 0HSP | A0A3B6C7U0 | FAD-binding FR-type domain-containing protein | 0HSP | A0A3B6D8P8 | J domain-containing protein |
| 0HSP | A0A3B6RKP0 | Far upstream element-binding protein 3 | 0HSP | A0A3B5Y7Y1 | Ketol-acid reductoisomerase |
| 0HSP | A0A3B6DCN1 | Fatty acid biosynthesis1 | 0HSP | W5HY47 | KOW domain-containing protein |
| 0HSP | A0A3B6QK55 | Fatty acid desaturase DES2 | 0HSP | A0A3B6PTB4 | L-ascorbate peroxidase |
| 0HSP | A0A3B6EIA3 | Fe-S cluster assembly factor HCF101, chloroplastic | 0HSP | A0A3B6NXB6 | L-ascorbate peroxidase |
| 0HSP | A0A3B6R6V9 | Ferredoxin-NADP reductase, chloroplastic | 0HSP | A0A3B6SCP3 | L-ascorbate peroxidase |
| 0HSP | A0A3B6I733 | Ferredoxin-NADP reductase, chloroplastic | 0HSP | A0A3B6KEY6 | L-ascorbate peroxidase |
| 0HSP | A0A3B6PQS3 | Ferredoxin-nitrite reductase | 0HSP | A0A3B6JIA4 | Lactamase_B domain-containing protein |
| 0HSP | A0A3B6CF64 | Fn3_like domain-containing protein | 0HSP | A0A3B5ZQU4 | Lipase_3 domain-containing protein |
| 0HSP | A0A3B6LYU6 | Fructan exohydrolase | 0HSP | A0A3B6AVN3 | Lipase_GDSL domain-containing protein |
| 0HSP | A0A3B5Y285 | G domain-containing protein | 0HSP | A0A3B6PLP6 | Lipoxygenase |
| 0HSP | A0A3B6QEH8 | Gamma hydroxybutyrate dehydrogenase-like protein | 0HSP | A0A3B6CF89 | Lipoxygenase |
| 0HSP | A0A3B6D692 | Germin-like protein | 0HSP | A0A341WW51 | Lipoxygenase |
| 0HSP | W5H014 | GLTP domain-containing protein | 0HSP | A0A3B6MKA4 | Lipoxygenase |
| 0HSP | A0A3B5XVL2 | Glucose and ribitol dehydrogenase-like protein | 0HSP | A0A3B6LF12 | Lipoxygenase |
| 0HSP | A0A3B6NN83 | Glucose-6-phosphate 1-dehydrogenase | 0HSP | A0A3B6MID4 | Lipoxygenase |

| Category | Accession | Description | Category | Accession | Description |
| --- | --- | --- | --- | --- | --- |
| 0HSP | A0A3B6QI43 | Lysine-tRNA ligase | 0HSP | A0A3B6A3S4 | Nucleoside diphosphate kinase |
| 0HSP | A0A3B6QKG3 | M16C_associated domain-containing protein | 0HSP | A0A077RVD7 | OB-fold nucleic acid binding domain containing protein |
| 0HSP | A0A3B6ITC2 | Macro domain-containing protein | 0HSP | A0A3B6CFB2 | OSJNBb0003B01.9 protein |
| 0HSP | W5FIX9 | Macrophage migration inhibitory factor family protein, expressed | 0HSP | A0A3B6C9T3 | OSJNBb0039L24.13 protein |
| 0HSP | A0A3B6AUK1 | Magnesium chelatase | 0HSP | A0A3B6PPI5 | Outer envelope pore protein 16-3, chloroplastic/mitochondrial |
| 0HSP | A0A3B6TRX0 | Malate dehydrogenase (NADP^+^) | 0HSP | A0A1D5W9E8 | Oxidored_q6 domain-containing protein |
| 0HSP | A0A3B6MY99 | Malate dehydrogenase | 0HSP | D3K1B4 | Ozone-responsive stress-related protein |
| 0HSP | A0A3B6LSL8 | Malate dehydrogenase | 0HSP | A0A3B6QH72 | p-loop containing nucleoside triphosphate hydrolase superfamily protein |
| 0HSP | A0A3B6GWS9 | Malate dehydrogenase | 0HSP | A0A3B6CE00 | PAP_fibrillin domain-containing protein |
| 0HSP | A0A3B6FS40 | MAR-binding filament-like protein 1-1 isoform 2 | 0HSP | A0A3B6N2H4 | PCI domain-containing protein |
| 0HSP | A0A3B6GPQ3 | MAR-binding filament-like protein 1 | 0HSP | A0A3B5XW00 | PDZ domain-containing protein |
| 0HSP | A0A3B6FLG3 | MAR-binding filament-like protein 1 | 0HSP | A0A3B6A1A2 | PDZ domain-containing protein |
| 0HSP | A0A3B6EE64 | MAR-binding filament-like protein 1 | 0HSP | A0A3B6DM97 | Pentatricopeptide repeat-containing protein |
| 0HSP | A0A3B6MYP1 | MBD domain-containing protein | 0HSP | A0A3B5YZ06 | Peptidase_M24 domain-containing protein |
| 0HSP | A0A125RLL8 | MBD6-5AS | 0HSP | A0A3B6TMI3 | Peptidase_S26 domain-containing protein |
| 0HSP | W5G255 | MBD6-5DS | 0HSP | A0A3B6HVS6 | Peptidase, M50 family |
| 0HSP | A0A3B5XY55 | Metallopeptidase M24 family protein | 0HSP | C0J025 | Peptidyl-prolyl cis-trans isomerase |
| 0HSP | A0A3B5ZQ95 | Methenyltetrahydrofolate cyclohydrolase | 0HSP | A0A3B6EH86 | Peptidyl-prolyl cis-trans isomerase |
| 0HSP | A0A3B6PI86 | Methenyltetrahydrofolate cyclohydrolase | 0HSP | A0A3B6PP75 | Peptidylprolyl isomerase |
| 0HSP | A0A3B6HSM4 | Methionyl-tRNA synthetase | 0HSP | A0A3B6TM18 | Peptidylprolyl isomerase |
| 0HSP | A0A3B6IVP5 | Methionyl-tRNA synthetase | 0HSP | A0A3B6U675 | Peptidylprolyl isomerase |
| 0HSP | A0A3B6LW30 | Mg-protoporphyrin IX chelatase | 0HSP | A0A3B6JJ78 | Phosphoenolpyruvate carboxykinase (ATP) |
| 0HSP | A0A3B6TX84 | Mg-protoporphyrin IX chelatase | 0HSP | A0A077RRA0 | Phosphoenolpyruvate carboxylase |
| 0HSP | A0A3B5YWR8 | mitochondrial carnitine/acylcarnitine carrier-like protein | 0HSP | W5D322 | Phosphoglycerate mutase (2,3-diphosphoglycerate-independent) |
| 0HSP | A0A3B6MPR0 | Mitochondrial outer membrane porin | 0HSP | A0A3B6LJ28 | Phosphoglycolate phosphatase |
| 0HSP | A0A3B6HQH0 | Mitochondrial-processing peptidase beta subunit | 0HSP | W5D4Q6 | Phospholipase D |
| 0HSP | A0A3B6GUF4 | MoCF_biosynth domain-containing protein | 0HSP | A0A3B6HRZ3 | Phosphoserine aminotransferase |
| 0HSP | A0A3B6MQU5 | Monogalactosyldiacylglycerol synthase | 0HSP | A0A3B6LPN8 | Photolyase/cryptochrome alpha/beta domain-containing protein |
| 0HSP | A0A3B6DLZ0 | MPN domain-containing protein | 0HSP | P62720 | Photosystem I assembly protein Ycf4 |
| 0HSP | W5CZ06 | Mucin-like protein | 0HSP | A0A3B6NWL9 | Photosystem II 10 kDa polypeptide, chloroplastic |
| 0HSP | A0A3B6RGV6 | Multiple organellar RNA editing factor 9, chloroplastic-like | 0HSP | W5D509 | Photosystem II reaction center Psb28 protein |
| 0HSP | A0A3B6PLA1 | Myb-like domain-containing protein | 0HSP | A0A3B6TSQ3 | PI-PLC X domain-containing protein |
| 0HSP | A0A3B6H333 | Myosin-like protein | 0HSP | A0A3B6TEG7 | PKS_ER domain-containing protein |
| 0HSP | A0A3B6ELF1 | NAD(P)-bd_dom domain-containing protein | 0HSP | A0A3B6KUA5 | PKS_ER domain-containing protein |
| 0HSP | A0A3B6N2D1 | NAD(P)-bd_dom domain-containing protein | 0HSP | A0A3B6KID6 | PKS_ER domain-containing protein |
| 0HSP | A0A3B6DDI9 | NAD(P)-bd_dom domain-containing protein | 0HSP | A0A3B6C650 | Plasma membrane intrinsic protein |
| 0HSP | A0A3B6QPF6 | NAD(P)-bd_dom domain-containing protein | 0HSP | A0A3B6MNL5 | Polyadenylate-binding protein |
| 0HSP | A0A3B6QCE9 | NAD(P)-bd_dom domain-containing protein | 0HSP | A0A3B6RJH1 | Polyadenylate-binding protein |
| 0HSP | A0A3B6KSP7 | NAD(P)-bd_dom domain-containing protein | 0HSP | A0A3B6H0R4 | Potassium transporter 2 |
| 0HSP | A0A3B6C0B9 | NAD(P)-binding Rossmann-fold superfamily protein | 0HSP | A0A3B5XZG7 | Prefoldin subunit 5 |
| 0HSP | Q95H42 | NAD(P)H-quinone oxidoreductase subunit H, chloroplastic | 0HSP | A0A3B6TMV0 | Probable alanine-tRNA ligase, chloroplastic |
| 0HSP | W5EGE3 | NADH dehydrogenase [ubiquinone] 1 beta subcomplex subunit 7 | 0HSP | A0A3B5Z1F6 | Proline-rich family protein |
| 0HSP | A0A3B6JFJ4 | NADH dehydrogenase [ubiquinone] iron-sulfur protein 1, mitochondrial | 0HSP | A0A1D5YY58 | Proteasome subunit alpha type |
| 0HSP | W5C2Z7 | NADH dehydrogenase [ubiquinone] iron-sulfur protein 4, mitochondrial | 0HSP | W5G0E6 | Proteasome subunit alpha type |
| 0HSP | W5CHL9 | Neuronal acetylcholine receptor subunit alpha | 0HSP | A0A3B6JH57 | Proteasome subunit beta |
| 0HSP | A0A3B5ZR64 | NFACT-R_1 domain-containing protein | 0HSP | A0A3B6RL68 | Protein cbbY |
| 0HSP | A0A3B6QJ10 | Nitrite reductase | 0HSP | A0A3B6PSW8 | Protein DCL chloroplastic |
| 0HSP | A0A3B6RIZ4 | NmrA domain-containing protein | 0HSP | A0A3B6KHN0 | Protein DETOXIFICATION |
| 0HSP | A0A3B6SH56 | NmrA domain-containing protein | 0HSP | Q93XQ7 | Protein disulfide-isomerase |
| 0HSP | W5CT58 | Nucleic acid-binding protein | 0HSP | A0A3B6HYX1 | Protein disulfide-isomerase |

| Category | Accession | Description | Category | Accession | Description |
| --- | --- | --- | --- | --- | --- |
| 0HSP | A0A3B5YQ83 | Protein EMBRYO SAC DEVELOPMENT ARREST 3, chloroplastic | 0HSP | A0A3B6MKF1 | Ribosomal_S10 domain-containing protein |
| 0HSP | A0A3B6JK46 | Protein IN2-1-like protein B | 0HSP | W5I035 | Ribosomal_S13_N domain-containing protein |
| 0HSP | A0A3B5ZZX9 | Protein kinase domain-containing protein | 0HSP | W5BFB7 | Ribosomal_S17_N domain-containing protein |
| 0HSP | A0A3B6UBM4 | Protein kinase domain-containing protein | 0HSP | P11383 | Ribulose bisphosphate carboxylase large chain |
| 0HSP | A0A3B6PUW8 | Protein kinase domain-containing protein | 0HSP | A0A3B6BYP2 | Ribulose bisphosphate carboxylase small chain |
| 0HSP | A0A3B6INI6 | Protein kinase domain-containing protein | 0HSP | W5E5Z3 | Ribulose-phosphate 3-epimerase |
| 0HSP | A0A3B6EHP4 | Protein PLASTID MOVEMENT IMPAIRED 2 | 0HSP | A0A3B6DAA8 | Ricin B-like lectin R40G2 |
| 0HSP | A0A3B6JK48 | Protein Rf1, mitochondrial-like | 0HSP | W5GYP9 | RRM domain-containing protein |
| 0HSP | A0A3B6FKQ2 | Protein translocase subunit SecA | 0HSP | A0A3B6RB14 | RuBisCO large subunit-binding protein subunit beta, chloroplastic |
| 0HSP | A0A3B6GQJ1 | Protein translocase subunit SecA | 0HSP | A0A3B6PFM5 | S1 motif domain-containing protein |
| 0HSP | A0A3B6NWI0 | Protein TSS | 0HSP | W5ATK7 | Salt tolerant protein |
| 0HSP | A0A3B6GZM3 | Protein TSS | 0HSP | A0A3B6HV43 | SelT/selW/selH selenoprotein domain containing protein |
| 0HSP | A0A3B6C1Y1 | Protein-serine/threonine kinase | 0HSP | A0A0C4BJE5 | Serine hydroxymethyltransferase |
| 0HSP | W5B1P6 | Protein-serine/threonine phosphatase | 0HSP | A0A3B6IU93 | Seryl-tRNA synthetase |
| 0HSP | H9C8A6 | Proton gradient regulation 5 | 0HSP | A0A3B6GLL5 | Shikimate kinase family protein |
| 0HSP | A0A3B6MXE7 | PSI-F | 0HSP | A0A3B6KJ32 | SHSP domain-containing protein |
| 0HSP | A0A3B6U0E4 | PSII_BNR domain-containing protein | 0HSP | W5B7G8 | Sm domain-containing protein |
| 0HSP | A0A3B6JMU6 | Purple acid phosphatase | 0HSP | A0A3B6AZ37 | Sm domain-containing protein |
| 0HSP | A0A077RX76 | Putative acyl transferase 3 | 0HSP | A0A3B6MJY6 | Small GTP-binding protein |
| 0HSP | A0A3B6APF8 | Putative CBS domain-containing protein | 0HSP | W5D4D4 | Sodium/calcium exchanger NCL1 |
| 0HSP | A0A3B6TRV7 | Putative glutamate/malate translocator | 0HSP | A0A3B6A1R3 | Solanesyl-diphosphate synthase 2, chloroplastic |
| 0HSP | A0A3B6DK94 | Putative glutathione S-transferase GSTU6 | 0HSP | A0A3B6HUD3 | SRP54 domain-containing protein |
| 0HSP | A0A3B6GP35 | Putative hydroquinone glucosyltransferase | 0HSP | A0A3B6IQE4 | SRP54 domain-containing protein |
| 0HSP | A0A3B6KQS3 | Putative NADH-ubiquinone | 0HSP | A0A3B6S8B4 | Starch synthase, chloroplastic/amyloplastic |
| 0HSP | A0A1D5UGI9 | Putative oxidoreductase | 0HSP | A0A3B6LU07 | Succinate dehydrogenase [ubiquinone] flavoprotein subunit, mitochondrial |
| 0HSP | A0A3B6IU00 | Putative rhamnose biosynthetic enzyme 1 | 0HSP | A0A3B6TQC1 | Sulfite oxidase |
| 0HSP | A0A3B6I1H3 | Putative thiamine biosythesis protein ThiC | 0HSP | A0A3B6MWE0 | Sulfotransferase |
| 0HSP | E6Y3A2 | Putative translation initiation factor | 0HSP | A0A3B6CDX0 | SWIB complex BAF60b domain-containing protein |
| 0HSP | A0A3B6IRV7 | Putative Xaa-Pro aminopeptidase 2 | 0HSP | A0A3B6QC33 | T-complex protein 1 subunit eta |
| 0HSP | A0A3B6H611 | Pyridoxal phosphate homeostasis protein | 0HSP | A0A3B6SMZ6 | T-complex protein 1 subunit gamma |
| 0HSP | A0A1D6D1Q3 | Pyrophosphate-fructose 6-phosphate 1-phosphotransferase subunit beta | 0HSP | A0A3B6INC2 | TCTP domain-containing protein |
| 0HSP | A0A3B6MYS2 | Pyruvate dehydrogenase E1 component subunit beta | 0HSP | A0A3B6CEX1 | Tetratricopeptide repeat (TPR)-like superfamily protein |
| 0HSP | A0A1D5W7M9 | Ras-related protein RIC1 | 0HSP | A0A3B6DB45 | Tetratricopeptide repeat containing protein |
| 0HSP | A0A3B6AVR1 | Reactive Intermediate Deaminase A, chloroplastic | 0HSP | A0A3B6C4Z5 | Tetratricopeptide repeat containing protein |
| 0HSP | V9HXE6 | Remorin REMO1-b | 0HSP | A0A3B6TUD9 | Thiamine thiazole synthase, chloroplastic |
| 0HSP | A0A3B6U3N3 | RF_PROK_I domain-containing protein | 0HSP | A0A3B6PS76 | Thioredoxin domain-containing protein |
| 0HSP | A0A3B6QHC8 | Rhodanese domain-containing protein | 0HSP | A0A3B6IMD4 | Thioredoxin domain-containing protein |
| 0HSP | A0A3B6ITL8 | Rhodanese domain-containing protein | 0HSP | A0A3B6DBB2 | Thioredoxin domain-containing protein |
| 0HSP | A0A3B6FTI5 | Rhodanese domain-containing protein | 0HSP | A0A3B5XWU3 | Thioredoxin domain-containing protein |
| 0HSP | A0A3B6H196 | Rhodanese domain-containing protein | 0HSP | O64394 | Thioredoxin H-type |
| 0HSP | A0A3B6MYQ9 | Ribose-5-phosphate isomerase | 0HSP | A0A3B5ZYU5 | Thioredoxin |
| 0HSP | A0A3B6QCR5 | Ribose-5-phosphate isomerase | 0HSP | A0A3B5XYZ9 | Thylakoid lumenal 17 protein, chloroplastic |
| 0HSP | Q5I7K4 | Ribosomal protein L17 | 0HSP | A0A3B5Z049 | TPR_MalT domain-containing protein |
| 0HSP | A0A1D5W7I4 | Ribosomal protein | 0HSP | D8LAL0 | TPR_REGION domain-containing protein |
| 0HSP | A0A3B6HZM8 | Ribosomal protein | 0HSP | A0A3B6TS42 | Tr-type G domain-containing protein |
| 0HSP | A0A3B6I5R9 | Ribosomal RNA small subunit methyltransferase G | 0HSP | A0A3B6C6P4 | Transket_pyr domain-containing protein |
| 0HSP | A0A3B6PL02 | Ribosomal_L14e domain-containing protein | 0HSP | E6Y289 | Translationally-controlled tumor protein |
| 0HSP | A0A0C4BIR6 | Ribosomal_L16 domain-containing protein | 0HSP | W5DXF3 | Translocase of chloroplast |
| 0HSP | W5BPV2 | Ribosomal_L18e/L15P domain-containing protein | 0HSP | A0A3B6SBB4 | Transmembrane protein 245 |
| 0HSP | A0A3B6IVC6 | Ribosomal_L18e/L15P domain-containing protein | 0HSP | A0A3B6NRW7 | Tripeptidyl-peptidase II |

| Category | Accession | Description | Category | Accession | Description |
| --- | --- | --- | --- | --- | --- |
| 0HSP | A0A3B5Y749 | Tryptophan synthase | 24HUP | W5AY52 | Chlorophyll a-b binding protein, chloroplastic |
| 0HSP | A0A3B5XZ27 | Tyrosine-tRNA ligase | 24HUP | A0A2X0S1W6 | Chloroplast ferredoxin-dependent glutamate synthase |
| 0HSP | A0A3B5YTT3 | U1 small nuclear ribonucleoprotein A | 24HUP | A0A3B5XZJ7 | Chloroplast inner envelope protein, putative, expressed |
| 0HSP | A0A3B6NM58 | UBA domain-containing protein | 24HUP | A0A3B5ZT18 | Chloroplast inner envelope protein, putative, expressed |
| 0HSP | A0A3B6QCE5 | Ubiquitin receptor RAD23 | 24HUP | A0A3B5YVW2 | Chloroplast inner envelope protein, putative, expressed |
| 0HSP | W5GQY7 | Ubiquitin thioesterase OTU1 | 24HUP | A0A3B6UAW0 | Clathrin light chain |
| 0HSP | A0A3B6DC18 | Ubiquitinyl hydrolase 1 | 24HUP | A0A3B6JR58 | Cold acclimation protein WCOR615 |
| 0HSP | A0A3B6DAP6 | Usp domain-containing protein | 24HUP | A0A3B6EFA0 | Cold induced protein |
| 0HSP | A0A1D6S518 | UTP-glucose-1-phosphate uridylyltransferase | 24HUP | A0A172WCB1 | Cold-responsive LEA/RAB-related COR protein |
| 0HSP | W5BT22 | UV-B-induced protein chloroplastic | 24HUP | A0A3B6ISV8 | Cystathionine gamma-synthase, chloroplastic |
| 0HSP | W5H1G1 | Vacuolar proton pump subunit B | 24HUP | A0A3B6KP84 | Cytochrome b-c1 complex subunit 7 |
| 0HSP | A0A3B5ZRH5 | VOC domain-containing protein | 24HUP | P05151 | Cytochrome f |
| 0HSP | A0A3B6IS82 | VOC domain-containing protein | 24HUP | A0A3B6PSA8 | CYTOSOL_AP domain-containing protein |
| 0HSP | A0A3B6A021 | Voltage dependent anion channel (VDAC) (Fragment) | 24HUP | D8L9K9 | D-fructose-1,6-bisphosphate 1-phosphohydrolase |
| 0HSP | A0A3B6FLS2 | VPE4B protein | 24HUP | A0A3B5XVD9 | Dehydroascorbate reductase |
| 0HSP | A0A3B6JJG0 | VWFA domain-containing protein | 24HUP | A0A3B6EFP8 | DREPP4 protein |
| 0HSP | A0A341UGW3 | W2 domain-containing protein | 24HUP | A0A3B6GTT8 | DREPP4 protein |
| 0HSP | A0A3B6U4W5 | WD_REPEATS_REGION domain-containing protein | 24HUP | A0A3B6PFS9 | DUF1995 domain-containing protein |
| 0HSP | W5EP05 | WD_REPEATS_REGION domain-containing protein | 24HUP | A0A3B6DB70 | EF1_GNE domain-containing protein |
| 0HSP | A0A3B6EAV0 | WD_REPEATS_REGION domain-containing protein | 24HUP | A0A3B6KBM4 | Elongation factor Ts, mitochondrial |
| 0HSP | A0A3B6IWE6 | WPP domain-containing protein | 24HUP | A0A3B6PNI9 | Elongation factor Tu |
| 0HSP | A0A3B6KJ15 | ZnF_CDGSH domain-containing protein | 24HUP | R9W6A6 | ER molecular chaperone |
| 24HUP | L0GED8 | 14-3-3 protein | 24HUP | A0A3B6H5M6 | Expressed protein |
| 24HUP | A0A3B6GUW3 | 30S ribosomal protein S20, chloroplastic | 24HUP | A0A3B6R6V9 | Ferredoxin-NADP reductase, chloroplastic |
| 24HUP | A0A3B6QG71 | 30S ribosomal protein S21 | 24HUP | A0A3B6NH85 | Ferredoxin-NADP reductase, chloroplastic |
| 24HUP | W5FPA7 | 30S ribosomal protein S4, chloroplastic | 24HUP | A0A3B6SIC2 | Glutamate-1-semialdehyde 2,1-aminomutase |
| 24HUP | Q1XIR9 | 4-hydroxy-7-methoxy-3-oxo-3,4-dihydro-2H-1,4-benzoxazin-2-yl glucoside beta-D-glucosidase 1a, chloroplastic | 24HUP | A0A3B6JP20 | Group3 late embryogenesis abundant protein |
| 24HUP | W5BSX7 | 40S ribosomal protein S10-1 | 24HUP | A0A3B6C7G7 | GrpE protein homolog |
| 24HUP | A0A3B6KQB8 | 40S ribosomal protein S26 | 24HUP | W5GLX4 | Heat shock cognate 70 kDa protein 1 |
| 24HUP | W5C8N6 | 40S ribosomal protein S6 | 24HUP | A0A1D5YKH2 | Histone H2A |
| 24HUP | A0A3B6C6G0 | 40S ribosomal protein S8 | 24HUP | A0A3B6RKQ5 | HMA domain-containing protein |
| 24HUP | A0A1D5UKZ4 | 40S ribosomal protein S8 | 24HUP | A0A3B6B107 | Indole-3-glycerol-phosphate synthase |
| 24HUP | A0A3B6ITQ9 | 50S ribosomal protein L10, chloroplastic | 24HUP | W5HY47 | KOW domain-containing protein |
| 24HUP | A0A3B6QHZ8 | 50S ribosomal protein L19, chloroplastic | 24HUP | A0A3B6NXB6 | L-ascorbate peroxidase |
| 24HUP | Q95H48 | 50S ribosomal protein L22, chloroplastic | 24HUP | A0A3B6PTB4 | L-ascorbate peroxidase |
| 24HUP | W5H631 | 60S ribosomal protein L27 | 24HUP | A0A3B6MKA4 | Lipoxygenase |
| 24HUP | A0A3B5XZW7 | AAI domain-containing protein | 24HUP | A0A3B6MID4 | Lipoxygenase |
| 24HUP | Q76ME3 | ADP-ribosylation factor | 24HUP | A0A3B6QAJ9 | Mitochondrial ATP synthase |
| 24HUP | A0A3B6SHQ4 | Alanine-glyoxylate aminotransferase | 24HUP | A0A3B5YWR8 | mitochondrial carnitine/acylcarnitine carrier-like protein |
| 24HUP | D2KZ08 | Aminotransferase | 24HUP | A0A3B6SD90 | Myosin heavy chain-related |
| 24HUP | A0A3B5Z514 | Asparagine synthetase [glutamine-hydrolyzing] | 24HUP | A0A3B6GV04 | NAD(P)-bd_dom domain-containing protein |
| 24HUP | A0A3B5XWA7 | Asparagine-tRNA ligase | 24HUP | A0A3B6ELF1 | NAD(P)-bd_dom domain-containing protein |
| 24HUP | Q332R4 | ATP synthase subunit alpha | 24HUP | A0A3B5XTZ8 | NAD(P)-bd_dom domain-containing protein |
| 24HUP | P12112 | ATP synthase subunit alpha, chloroplastic | 24HUP | A0A3B5ZVK4 | NADH-ubiquinone oxidoreductase, putative, expressed |
| 24HUP | P06528 | ATP synthase subunit b, chloroplastic | 24HUP | A0A3B5XZQ9 | NADPH-protochlorophyllide oxidoreductase |
| 24HUP | A0A3B5Y5K9 | ATP synthase subunit beta | 24HUP | A0A3B6MMT9 | Nucleoside diphosphate kinase |
| 24HUP | A0A3B5ZT15 | ATP synthase subunit gamma | 24HUP | W5B9L0 | Oxygen evolving enhancer protein |
| 24HUP | A0A341QMC1 | ATP synthase subunit | 24HUP | A0A3B5ZUB2 | PAP_fibrillin domain-containing protein |
| 24HUP | A0A3B6GT39 | Carbonic anhydrase | 24HUP | A0A3B6QCI9 | Peroxidase |
| 24HUP | A0A341YEW4 | Chlorophyll a-b binding protein, chloroplastic | 24HUP | A0A3B6CC52 | PEROXIDASE_4 domain-containing protein |

| Category | Accession | Description | Category | Accession | Description |
| --- | --- | --- | --- | --- | --- |
| 24HUP | Q5S1S6 | Peroxiredoxin Q, chloroplastic | 24HDN | A0A3B6QG11 | 50S ribosomal protein L21, chloroplastic |
| 24HUP | A0A3B6HW60 | Phosphoglucomutase (alpha-D-glucose-1,6-bisphosphate-dependent) | 24HDN | F4YWL9 | 60S ribosomal protein L21 |
| 24HUP | A0A3B5Z298 | Phosphoglycerate kinase | 24HDN | W5C5N9 | 60S ribosomal protein L22-2 |
| 24HUP | W5D4Q6 | Phospholipase D | 24HDN | W5G990 | 60S ribosomal protein L31 |
| 24HUP | P26302 | Phosphoribulokinase, chloroplastic | 24HDN | A0A0C4BKI1 | 60S ribosomal protein L35a-1 |
| 24HUP | P69555 | Photosystem II reaction center protein H | 24HDN | D8L9P6 | 60S ribosomal protein L37a, expressed |
| 24HUP | A0A3B6FKL0 | PNP_UDP_1 domain-containing protein | 24HDN | A0A077RXS4 | AAA domain-containing protein |
| 24HUP | A0A3B6N207 | Pre-mRNA processing factor | 24HDN | A0A3B6ISQ8 | ABC transporter domain-containing protein |
| 24HUP | W5G0E6 | Proteasome subunit alpha type | 24HDN | A0A2P1AAU6 | ABCF1d |
| 24HUP | A0A3B6JDZ5 | Proteasome subunit beta | 24HDN | A0A1D6D5I2 | ACB domain-containing protein |
| 24HUP | A0A3B6HYX1 | Protein disulfide-isomerase | 24HDN | A0A3B6I0D2 | ACB domain-containing protein |
| 24HUP | A0A3B6DFX2 | PsbP domain-containing protein | 24HDN | A0A3B6FKB3 | Aconitate hydratase |
| 24HUP | A0A3B6MXE7 | PSI-F | 24HDN | A0A3B6PM63 | Adenosine kinase |
| 24HUP | A0A3B6IL99 | Putative 30S ribosomal protein S13 | 24HDN | A0A3B6UBC9 | Agglutinin domain-containing protein |
| 24HUP | A0A3B5ZUP0 | Putative chloroplast inner envelope protein (Fragment) | 24HDN | A0A3B6SHQ4 | Alanine-glyoxylate aminotransferase |
| 24HUP | A0A3B5XYP5 | Putative chloroplast inner envelope protein (Fragment) | 24HDN | A0A3B6SGJ8 | Aldedh domain-containing protein |
| 24HUP | A0A3B6B7R8 | Putative glutathione S-transferase GSTU6 | 24HDN | A0A3B5ZSJ8 | Alpha-galactosidase |
| 24HUP | W5C4P1 | Putative oxygen-evolving complex | 24HDN | A0A3B6H2I8 | Amidophosphoribosyltransferase |
| 24HUP | A0A3B6EM75 | Rhodanese domain-containing protein | 24HDN | A0A3B6N401 | ANK_REP_REGION domain-containing protein |
| 24HUP | A0A3B6MQM1 | Rhodanese domain-containing protein | 24HDN | G3E8E1 | Aquaporin 7 |
| 24HUP | A0A3B6H196 | Rhodanese domain-containing protein | 24HDN | A0A1D6AM47 | Aquaporin |
| 24HUP | A0A3B6QHC8 | Rhodanese domain-containing protein | 24HDN | A0A3B6IV09 | bPH_2 domain-containing protein |
| 24HUP | A0A3B6C571 | Ribosomal_L18e/L15P domain-containing protein | 24HDN | A0A3B6PI06 | Calnexin-like protein |
| 24HUP | A0A1D5UQZ5 | Ribosomal_L23eN domain-containing protein | 24HDN | A0A3B6GT39 | Carbonic anhydrase |
| 24HUP | W5I035 | Ribosomal_S13_N domain-containing protein | 24HDN | A0A3B6PPE6 | Carboxypeptidase |
| 24HUP | P11383 | Ribulose bisphosphate carboxylase large chain | 24HDN | F1DKC1 | Catalase |
| 24HUP | A0A3B6BYP2 | Ribulose bisphosphate carboxylase small chain | 24HDN | A0A3B6HUP2 | CCT-theta |
| 24HUP | A0A3B6RB14 | RuBisCO large subunit-binding protein subunit beta, chloroplastic | 24HDN | A0A3B5ZRV7 | Chaperonin CPN60-2, mitochondrial |
| 24HUP | A0A0C4BJE5 | Serine hydroxymethyltransferase | 24HDN | A0A3B6UAW0 | Clathrin light chain |
| 24HUP | A0A3B6TID0 | Transketolase | 24HDN | A0A3B6U6K4 | Clp R domain-containing protein |
| 24HUP | E6Y289 | Translationally-controlled tumor protein | 24HDN | A0A3B6CDD2 | Coiled-coil domain-containing protein 124 |
| 24HUP | A0A3B6LR33 | Triosephosphate isomerase, chloroplastic | 24HDN | S6AWC2 | Cold induced 16 |
| 24HUP | A0A3B6DE42 | Usp domain-containing protein | 24HDN | W5D5R6 | Cold induced protein |
| 24HUP | A0A3B6DCQ0 | VDE domain-containing protein | 24HDN | A0A3B6EFA0 | Cold induced protein |
| 24HDN | Q7DLM1 | (1,31,4) beta glucanase | 24HDN | A0A172WCB1 | Cold-responsive LEA/RAB-related COR protein |
| 24HDN | A0A341PIP8 | 2-oxoglutarate/malate translocator | 24HDN | A0A3B6B480 | Cold-responsive protein WCOR14 |
| 24HDN | A0A1D6D1N1 | 20 kDa chaperonin, chloroplastic | 24HDN | A0A3B6KLP1 | Cysteine synthase |
| 24HDN | A0A3B5Z524 | 2Fe-2S ferredoxin-type domain-containing protein | 24HDN | P05151 | Cytochrome f |
| 24HDN | A0A3B6B8V8 | 3-isopropylmalate dehydrogenase | 24HDN | W5CY74 | DEAD-box ATP-dependent RNA helicase 25 |
| 24HDN | A0A3B6PSZ6 | 3-ketoacyl-CoA thiolase-like protein | 24HDN | A0A3B6PJY3 | Delta-aminolevulinic acid dehydratase |
| 24HDN | A0A3B6NV46 | 3-ketoacyl-CoA thiolase-like protein | 24HDN | A0A3B6MVZ0 | DHQ_synthase domain-containing protein |
| 24HDN | W5FPA7 | 30S ribosomal protein S4, chloroplastic | 24HDN | W5A874 | Dihydrolipoyl dehydrogenase |
| 24HDN | A0A3B6AU50 | 4-alpha-glucanotransferase | 24HDN | A0A3B6ES90 | Dolichyl-diphosphooligosaccharide-protein glycosyltransferase subunit 2 |
| 24HDN | A0A3B6RHV8 | 40S ribosomal protein S24 | 24HDN | A0A3B6EFP8 | DREPP4 protein |
| 24HDN | A0A3B6S9M7 | 40S ribosomal protein S30 | 24HDN | A0A3B6FNR1 | DREPP4 protein |
| 24HDN | A0A1D5SLI4 | 40S ribosomal protein S4 | 24HDN | A0A3B5ZNF9 | E3 UFM1-protein ligase 1 homolog |
| 24HDN | A0A3B6U6R2 | 4a-hydroxytetrahydrobiopterin dehydratase | 24HDN | A0A3B6KBM4 | Elongation factor Ts, mitochondrial |
| 24HDN | A0A3B6ITQ9 | 50S ribosomal protein L10, chloroplastic | 24HDN | A0A0C4BIT2 | Epimerase domain-containing protein |
| 24HDN | A0A3B6KU60 | 50S ribosomal protein L11, chloroplastic | 24HDN | A0A3B5ZVX1 | Epimerase domain-containing protein |
| 24HDN | A0A3B6QHZ8 | 50S ribosomal protein L19, chloroplastic | 24HDN | Q03389 | Eukaryotic translation initiation factor isoform 4E-2 |

| Category | Accession | Description | Category | Accession | Description |
| --- | --- | --- | --- | --- | --- |
| 24HDN | A0A3B6LIF0 | Expressed protein | 24HDN | A0A3B6I1J2 | PGR5-like protein 1B chloroplastic |
| 24HDN | A0A3B6KQE5 | Expressed protein | 24HDN | A0A3B5Z298 | Phosphoglycerate kinase |
| 24HDN | A0A3B6RKP0 | Far upstream element-binding protein 3 | 24HDN | A0A3B6NNE0 | Phosphoglycerate kinase |
| 24HDN | A0A3B6SAV4 | Fe2OG dioxygenase domain-containing protein | 24HDN | A0A3B6LPN8 | Photolyase/cryptochrome alpha/beta domain-containing protein |
| 24HDN | A0A3B6I733 | Ferredoxin-NADP reductase, chloroplastic | 24HDN | A0A3B6HX37 | Photosynthetic NDH subunit of subcomplex B 1, chloroplastic |
| 24HDN | P00228 | Ferredoxin, chloroplastic | 24HDN | A7J2I2 | Plasma membrane intrinsic protein |
| 24HDN | A0A3B6NRA1 | FeThRed_A domain-containing protein | 24HDN | A0A1D6CXF2 | Proteasome subunit alpha type |
| 24HDN | Q84N28 | Flavone O-methyltransferase 1 | 24HDN | A0A3B6QAP5 | Proteasome subunit alpha type |
| 24HDN | A0A3B6MPP6 | Glutaredoxin domain-containing protein | 24HDN | A0A3B6SFC8 | Protein cbbY |
| 24HDN | A0A3B6EFJ3 | Glutaredoxin-dependent peroxiredoxin | 24HDN | A0A3B6MRQ5 | Protein CHAPERONE-LIKE PROTEIN OF POR1, chloroplastic |
| 24HDN | A0A3B6JM67 | Glutathione reductase | 24HDN | A0A3B6KHN0 | Protein DETOXIFICATION |
| 24HDN | A0A3B6RKE1 | Glyceraldehyde-3-phosphate dehydrogenase | 24HDN | A0A3B6KFF3 | Protein phosphatase 2A structural subunit |
| 24HDN | A0A3B6T784 | Glycine-tRNA ligase | 24HDN | W5CUJ0 | Protein phosphatase |
| 24HDN | A0A3B6EI00 | Glycosyltransferase | 24HDN | W5BRT4 | Putative prefoldin subunit 2 |
| 24HDN | A7VL25 | Group3 late embryogenesis abundant protein | 24HDN | E6Y0T1 | Putative SNAP receptor protein |
| 24HDN | A0A3B6JP20 | Group3 late embryogenesis abundant protein | 24HDN | A0A3B6IRV7 | Putative Xaa-Pro aminopeptidase 2 |
| 24HDN | A0A3B6C7G7 | GrpE protein homolog | 24HDN | A0A3B6NUN7 | Pyruvate dehydrogenase E1 component subunit alpha |
| 24HDN | A0A3B6EII0 | HABP4_PAI-RBP1 domain-containing protein | 24HDN | A0A3B6PG18 | Rhodanese domain-containing protein |
| 24HDN | A0A3B6MT71 | HATPase_c domain-containing protein | 24HDN | A0A3B6H196 | Rhodanese domain-containing protein |
| 24HDN | W5GLX4 | Heat shock cognate 70 kDa protein 1 | 24HDN | A0A3B6EM75 | Rhodanese domain-containing protein |
| 24HDN | A5HE90 | Hypersensitive response protein | 24HDN | A0A3B6EPN4 | Ribonuclease 2 |
| 24HDN | A0A3B6KR09 | Inosine-5'-monophosphate dehydrogenase | 24HDN | Q5I7L1 | Ribosomal protein L13a |
| 24HDN | A0A3B5Y7Y1 | Ketol-acid reductoisomerase | 24HDN | U5HTD8 | Ribosomal protein S20 |
| 24HDN | A0A3B6CF89 | Lipoxygenase | 24HDN | A0A3B6I5R9 | Ribosomal RNA small subunit methyltransferase G |
| 24HDN | M4VSR0 | Low temperature-responsive RNA-binding protein | 24HDN | A0A3B6C571 | Ribosomal_L18e/L15P domain-containing protein |
| 24HDN | A0A3B6QI43 | Lysine-tRNA ligase | 24HDN | W5I035 | Ribosomal_S13_N domain-containing protein |
| 24HDN | A0A3B6TRX0 | Malate dehydrogenase (NADP^+^) | 24HDN | W5BFB7 | Ribosomal_S17_N domain-containing protein |
| 24HDN | A0A3B6GPQ3 | MAR-binding filament-like protein 1 | 24HDN | A0A3B6H402 | RRM domain-containing protein |
| 24HDN | A0A3B6EE64 | MAR-binding filament-like protein 1 | 24HDN | A0A3B6RB14 | RuBisCO large subunit-binding protein subunit beta, chloroplastic |
| 24HDN | A0A3B6FLG3 | MAR-binding filament-like protein 1 | 24HDN | W5ATK7 | Salt tolerant protein |
| 24HDN | A0A3B6PI86 | Methenyltetrahydrofolate cyclohydrolase | 24HDN | A0A0C4BJE5 | Serine hydroxymethyltransferase |
| 24HDN | A0A3B6MPR0 | Mitochondrial outer membrane porin | 24HDN | A0A3B6CE87 | Serine hydroxymethyltransferase |
| 24HDN | K4HRS2 | Monodehydroascorbate reductase 4 | 24HDN | A0A3B6GLL5 | Shikimate kinase family protein |
| 24HDN | A0A3B6ELF1 | NAD(P)-bd_dom domain-containing protein | 24HDN | W5D4D4 | Sodium/calcium exchanger NCL1 |
| 24HDN | Q95H42 | NAD(P)H-quinone oxidoreductase subunit H, chloroplastic | 24HDN | A0A3B6HUD3 | SRP54 domain-containing protein |
| 24HDN | W5EHI0 | NADH dehydrogenase [ubiquinone] 1 alpha subcomplex subunit 13-B | 24HDN | A0A3B5ZWY5 | Subtilisin-like protease |
| 24HDN | A0A3B6C7H1 | NADH dehydrogenase [ubiquinone] flavoprotein 1, mitochondrial | 24HDN | A0A3B6LU07 | Succinate dehydrogenase [ubiquinone] flavoprotein subunit, mitochondrial |
| 24HDN | A0A3B6JFJ4 | NADH dehydrogenase [ubiquinone] iron-sulfur protein 1, mitochondrial | 24HDN | A0A3B6TMN0 | Sucrose synthase |
| 24HDN | A0A3B5XZQ9 | NADPH-protochlorophyllide oxidoreductase | 24HDN | A0A3B6TQC1 | Sulfite oxidase |
| 24HDN | A0A3B6QJ10 | Nitrite reductase | 24HDN | A0A3B6DB45 | Tetratricopeptide repeat containing protein |
| 24HDN | A0A3B6MMT9 | Nucleoside diphosphate kinase | 24HDN | A0A3B5XYZ9 | Thylakoid lumenal 17 protein, chloroplastic |
| 24HDN | A0A3B6A3S4 | Nucleoside diphosphate kinase | 24HDN | A0A3B6QC29 | Translation factor GUF1 homolog, chloroplastic |
| 24HDN | W5B9L0 | Oxygen evolving enhancer protein | 24HDN | A0A077RSI3 | Triosephosphat-isomerase |
| 24HDN | A0A3B5XW00 | PDZ domain-containing protein | 24HDN | A0A3B6LR33 | Triosephosphate isomerase, chloroplastic |
| 24HDN | A0A3B6A1A2 | PDZ domain-containing protein | 24HDN | W5GR34 | UBC core domain-containing protein |
| 24HDN | A0A3B6HV54 | Peptidase_S9 domain-containing protein | 24HDN | W5ADS2 | Ubiquitin |
| 24HDN | A0A3B6PP75 | Peptidylprolyl isomerase | 24HDN | A0A1D6S518 | UTP-glucose-1-phosphate uridylyltransferase |
| 24HDN | Q5S1S6 | Peroxiredoxin Q, chloroplastic |  |  |  |
