# Supplementary material for: Global Crotonylome Profiling Identifies TaPRXIIB Crotonylation as a Modulator H2O2 Homeostasis in Wheat Resistance to Puccinia triticina
Source: Mol Plant Pathol. 2026 Jul 11;27(7):e70288. doi: 10.1111/mpp.70288 (PMC13354946; doi:10.1111/mpp.70288)
Supplement: Supplementary file 10 — Table S4: Information on the interaction network of crotonylated proteins. [file MPP-27-e70288-s005.docx]

| **Table S4 Information on the interaction network of crotonylated proteins** | | | | | | | |
| --- | --- | --- | --- | --- | --- | --- | --- |
| Degree | Accession | Rename | Description | Degree | Accession | Rename | Description |
| 90 | W5ALC0 | PXP | Acyl-coenzyme A oxidase 4, peroxisomal | 37 | F1DKC1 | **TaCAT2** | Catalase |
| 86 | A0A3B6KIN5 | BADH | Aldedh domain-containing protein, aldehyde Dehydrogenase | 37 | D2KZ08 | TA | Aminotransferase |
| 76 | W5A874 | TaDLD | Dihydrolipoyl dehydrogenase, 2-hydroxyacyl-CoA dehydrogenase | 36 | Q7X9A6 | ISP | Cytochrome b6-f complex iron-sulfur subunit, chloroplastic |
| 76 | A0A3B6LU21 | CS | Citrate synthase, acetyl-CoA:oxaloacetate C-acetyltransferase | 36 | B2ZGL4 | AACase | Acetyl-CoA carboxylase |
| 75 | A0A0C4BJE5 | SHMT | Serine hydroxymethyltransferase | 36 | A0A3B6HMZ7 | AGT2 | Alanine-glyoxylate aminotransferase |
| 74 | A0A3B6JM67 | GR | Glutathione reductase | 36 | A0A3B6NN83 | G6PD1 | Glucose-6-phosphate 1-dehydrogenase |
| 73 | A0A3B6KQS3 | NDU | Putative NADH-ubiquinone | 35 | A0A3B6NH85 | LFNR1 | Ferredoxin-NADP reductase, chloroplastic |
| 70 | A0A3B6SHQ4 | AGT1 | Alanine-glyoxylate aminotransferase | 35 | A0A3B6A3S4 | NDPK1 | Nucleoside diphosphate kinase |
| 69 | A0A077RSI3 | ALT | Triosephosphat-isomerase | 35 | A0A3B6MMT9 | NDPK2 | Nucleoside diphosphate kinase |
| 62 | W5C4B7 | SUCL1 | Succinate-CoA ligase [ADP-forming] subunit alpha, mitochondrial | 34 | A0A3B6LI13 | HSD | Homoserine dehydrogenase |
| 61 | A0A3B5XU98 | G6PI1 | Glucose-6-phosphate isomerase | 34 | W5ADS2 | Ub | Ubiquitin |
| 61 | A0A3B6MTB1 | G6PI2 | Glucose-6-phosphate isomerase | 34 | A0A3B6R6V9 | LFNR2 | Ferredoxin-NADP reductase, chloroplastic |
| 59 | A0A3B6LWF0 | DLAT1 | Dihydrolipoamide acetyltransferase component of pyruvate dehydrogenase complex | 34 | A0A3B6I733 | LFNR3 | Ferredoxin-NADP reductase, chloroplastic |
| 59 | A0A3B6N0L8 | DLAT2 | Dihydrolipoamide acetyltransferase component of pyruvate dehydrogenase complex | 34 | A0A3B6C2L2 | UP3 | Stress-response A/B barrel domain-containing protein UP3 |
| 57 | A0A3B6TJJ8 | DHAD | Dihydroxy-acid dehydratase | 34 | A0A3B6ASE9 | GPDH | Glycerol-3-phosphate dehydrogenase |
| 55 | A0A3B6TEG7 | A0A3B6TEG7 | PKS_ER domain-containing | 32 | A0A3B6QHK3 | G6PD2 | Glucose-6-phosphate 1-dehydrogenase |
| 54 | A0A341WSR4 | HPR | Hydroxypyruvate reductase | 32 | A0A3B6KLP1 | AAS | Cysteine synthase |
| 53 | A0A1D6B2M0 | SUCL2 | Succinate-CoA ligase [ADP-forming] subunit beta, mitochondrial | 32 | A0A3B6ASP6 | A0A3B6ASP6 | Transket_pyr domain-containing protein |
| 53 | A0A077S3V2 | AST | Aspartate aminotransferase | 32 | A0A3B6C6P4 | A0A3B6C6P4 | Transket_pyr domain-containing protein |
| 52 | W5AY52 | LHC | Chlorophyll a-b binding protein, chloroplastic | 32 | Q95H42 | NQO | NAD(P)H-quinone oxidoreductase subunit H, chloroplastic |
| 51 | A0A3B6RMH1 | GLO1 | Glyoxalase I | 31 | D8L9G6 | αGP | Alpha-1,4 glucan phosphorylase |
| 51 | A0A3B6H4R5 | HPCL | 2-hydroxyacyl-CoA lyase | 31 | A0A1D6B308 | SAMS | S-adenosylmethionine synthase |
| 50 | A0A3B6JFJ4 | NDUFS1 | NADH dehydrogenase [ubiquinone] iron-sulfur protein 1, mitochondrial | 31 | A0A3B6KP84 | Uqcrb | Cytochrome b-c1 complex subunit 7 |
| 49 | A0A3B5ZRK2 | GLO2 | Glyoxalase I | 28 | W5FL09 | ALD1 | Fructose-bisphosphate aldolase |
| 49 | A0A3B6EHB2 | GLYR | Glyoxylate reductase | 28 | A0A3B6FHK1 | ALD2 | Fructose-bisphosphate aldolase |
| 48 | A0A3B6TID0 | TKT | Transketolase | 28 | A0A3B6Q8W6 | ARS | Alkylresorcinol synthase |
| 48 | A0A3B6IK29 | TaPGM1 | Phosphoglucomutase (alpha-D-glucose-1,6-bisphosphate-dependent) | 28 | A0A3B6AQ54 | AKR1 | Aldo_ket_red domain-containing protein |
| 48 | A0A3B6HW60 | TaPGM2 | Phosphoglucomutase (alpha-D-glucose-1,6-bisphosphate-dependent) | 27 | A0A3B6PFK2 | AKR2 | Aldo_ket_red domain-containing protein |
| 48 | A0A3B6MYS2 | PDH1 | Pyruvate dehydrogenase E1 component subunit beta | 27 | A0A3B5ZYK2 | AKR3 | Aldo_ket_red domain-containing protein |
| 47 | A0A3B6C7H1 | NDH | NADH dehydrogenase [ubiquinone] flavoprotein 1, mitochondrial | 27 | W5D122 | OxR | Putative oxidoreductase GLYR1 |
| 47 | W5IA32 | FDH | Formate dehydrogenase, mitochondrial | 27 | A0A3B6QEH8 | HBDH | Gamma hydroxybutyrate dehydrogenase-like protein |
| 46 | A0A3B6QEK4 | HADD | 3-hydroxyacyl-CoA dehydrogenase | 26 | A0A3B6QGV0 | KAR | B-keto acyl reductase |
| 45 | A0A1D5WW05 | PK | Pyruvate kinase | 26 | A0A3B6QPF6 | A0A3B6QPF6 | NAD(P)-bd_dom domain-containing protein |
| 44 | Q5S1S6 | TaPRX1 | Peroxiredoxin Q, chloroplastic | 26 | A0A3B6ELF1 | A0A3B6ELF1 | NAD(P)-bd_dom domain-containing protein |
| 44 | A0A3B6PSZ6 | KAT | 3-ketoacyl-CoA thiolase-like protein | 26 | A0A3B6GV04 | A0A3B6GV04 | NAD(P)-bd_dom domain-containing protein |
| 44 | A0A3B6QE21 | PTR | NADPH-dependent pterin aldehyde reductase | 26 | A0A3B6B781 | AHCY1 | Adenosylhomocysteinase |
| 44 | A0A3B5XVL2 | GRDH | Glucose and ribitol dehydrogenase-like protein | 26 | A0A3B6CE50 | AHCY2 | Adenosylhomocysteinase |
| 44 | A0A3B6NNX5 | 4CL | Putative 4-coumarate-CoA ligase 3 | 26 | A0A1D5W9E8 | A0A1D5W9E8 | Oxidored_q6 domain-containing protein |
| 43 | A0A3B6CEW9 | UCH | Ubiquitin carboxyl-terminal hydrolase 22 | 25 | A0A3B6NUI6 | GGR | Geranylgeranyl reductase |
| 43 | A0A3B6B901 | A0A3B6B901 | H0306F12.7 protein - | 25 | A0A3B6KID6 | A0A3B6KID6 | PKS_ER domain-containing protein |
| 41 | W5C2Z7 | NDUFS4 | NADH dehydrogenase [ubiquinone] iron-sulfur protein 4, mitochondrial | 24 | A0A3B5ZVK4 | CI | NADH-ubiquinone oxidoreductase, putative, expressed |
| 41 | A0A3B6NUN7 | PDH2 | Pyruvate dehydrogenase E1 component subunit alpha | 24 | W5EGE3 | NADFB7 | NADH dehydrogenase [ubiquinone] 1 beta subcomplex subunit 7 |
| 41 | W5C3E3 | PDH3 | Pyruvate dehydrogenase E1 component subunit alpha | 24 | A0A3B6N2D1 | A0A3B6N2D1 | NAD(P)-bd_dom domain-containing protein |
| 41 | A0A3B6KFM7 | IPMS | 2-isopropylmalate synthase | 24 | A0A3B6DDI9 | A0A3B6DDI9 | NAD(P)-bd_dom domain-containing protein |
| 40 | A0A3B6HV43 | SELT | SelT/selW/selH selenoprotein domain containing protein | 24 | A0A3B5XTZ8 | A0A3B5XTZ8 | NAD(P)-bd_dom domain-containing protein |
| 39 | A0A3B6ISV8 | CRS | Cystathionine gamma-synthase, chloroplastic (predicted) | 24 | A0A3B6KSP7 | A0A3B6KSP7 | NAD(P)-bd_dom domain-containing protein |
| 39 | A0A3B6HW46 | HDR1 | 4-hydroxy-3-methylbut-2-enyl diphosphate reductase (predicted) | 24 | A0A3B6SBT2 | A0A3B6SBT2 | Rhodanese domain-containing protein |
| 39 | A0A3B6JDC9 | HDR2 | 4-hydroxy-3-methylbut-2-enyl diphosphate reductase (predicted) | 24 | A0A3B6RIZ4 | A0A3B6RIZ4 | NmrA domain-containing protein |
| 38 | A0A3B6PM63 | ADK | Adenosine kinase | 24 | A0A3B6PG18 | A0A3B6PG18 | Rhodanese domain-containing protein |
| 37 | A0A3B6KT79 | ACO | Aconitate hydratase | 24 | A0A3B6QJ10 | NiRs | Nitrite reductase |

| Degree | Accession | Rename | Description | Degree | Accession | Rename | Description |
| --- | --- | --- | --- | --- | --- | --- | --- |
| 24 | A0A3B6NMG8 | A0A3B6NMG8 | PKS_ER domain-containing protein | 13 | A0A3B6C1Y1 | PLK | Protein-serine/threonine kinase |
| 23 | A0A1D6D1N1 | A0A1D6D1N1 | 20 kDa chaperonin, chloroplastic | 12 | A0A3B6EFJ3 | GPX | Glutaredoxin-dependent peroxiredoxin |
| 23 | A0A3B6RAV4 | A0A3B6RAV4 | 20 kDa chaperonin, chloroplastic | 11 | A0A3B6RL68 | CbbY1 | Protein cbbY |
| 23 | W5EHI0 | NDUFA13 | NADH dehydrogenase [ubiquinone] 1 alpha subcomplex subunit 13-B | 11 | A0A3B6SFC8 | CbbY2 | Protein cbbY |
| 22 | A0A3B6PSA8 | A0A3B6PSA8 | CYTOSOL_AP domain-containing protein | 11 | A0A3B6QI43 | KARS | Lysine-tRNA ligase |
| 22 | A0A3B6IMD4 | TRX1 | Thioredoxin domain-containing protein | 11 | A0A3B5XW00 | A0A3B5XW00 | PDZ domain-containing protein |
| 22 | A0A3B6C9S5 | TRX2 | Thioredoxin domain-containing protein | 11 | R9W6A6 | ERC | ER molecular chaperone |
| 21 | A0A1D5UUP9 | A0A1D5UUP9 | 10 kDa chaperonin | 10 | A0A3B6DB70 | A0A3B6DB70 | EF1_GNE domain-containing protein |
| 21 | A0A3B6AQN0 | A0A3B6AQN0 | Oxidored_FMN domain-containing protein | 10 | A0A3B6MN48 | AGPAT | 1-acylglycerol-3-phosphate O-acyltransferase |
| 21 | A0A3B6LW93 | A0A3B6LW93 | DPP6 N-terminal domain-like protein | 10 | A0A3B6A1A2 | A0A3B6A1A2 | PDZ domain-containing protein |
| 21 | A0A3B6CB33 | A0A3B6CB33 | OSJNBb0011N17.8 protein | 9 | A0A3B6PLP6 | LOX | Lipoxygenase |
| 21 | A0A3B6PS76 | TRX3 | Thioredoxin domain-containing protein | 7 | A0A3B6SH56 | A0A3B6SH56 | NmrA domain-containing protein |
| 21 | O64394 | TRX4 | Thioredoxin H-type | 6 | A0A3B6CEY1 | A0A3B6CEY1 | AB hydrolase-1 domain-containing protein |
| 20 | A0A3B5Y749 | TrpB | Tryptophan synthase | 6 | A0A3B6DPL3 | TaPRX3 | Peroxidase |
| 20 | W5EIQ2 | KCS | 3-ketoacyl-CoA synthase | 5 | A0A3B6MPP6 | GRX1 | Glutaredoxin domain-containing protein |
| 20 | A0A3B5YXL9 | Msr | Protein-methionine-S-oxide reductase | 5 | A0A3B6B887 | TaPRX4 | Peroxidase |
| 19 | A0A3B6JFX9 | UDP-GDH | UDP-glucose 6-dehydrogenase | 5 | A0A1D6D5I2 | A0A1D6D5I2 | ACB domain-containing protein |
| 19 | A0A3B6SIC2 | GSAAT | Glutamate-1-semialdehyde 2,1-aminomutase | 5 | A0A3B6QC29 | GUF1 | Translation factor GUF1 homolog, chloroplastic |
| 19 | A0A3B5XV05 | OTA | Acetylornithine transaminase | 5 | A0A3B6I0D2 | A0A3B6I0D2 | ACB domain-containing protein |
| 19 | A0A3B5XWU3 | TRX5 | Thioredoxin domain-containing protein | 5 | A0A3B6KJ15 | A0A3B6KJ15 | ZnF_CDGSH domain-containing protein |
| 18 | A0A3B6AVR1 | ADA | Reactive Intermediate Deaminase A, chloroplastic | 4 | A0A3B5YW44 | GRX2 | Monothiol glutaredoxin-S11 |
| 17 | A0A3B6SCP3 | APOX1 | L-ascorbate peroxidase | 4 | A0A3B6PS22 | DGK | diacylglycerol kinase |
| 17 | A0A3B6CG72 | IP | Intracellular protease 1 | 4 | A0A3B6CC52 | TaPRX5 | PEROXIDASE_4 domain-containing protein |
| 16 | A0A3B6SJX8 | A0A3B6SJX8 | Epimerase domain-containing protein | 4 | A0A3B6PH99 | TaPRX6 | Peroxidase |
| 16 | A0A3B6QI62 | GSH-Px | Glutathione peroxidase | 4 | A0A3B6CH98 | TaPRX7 | Peroxidase |
| 16 | A0A3B6NXB6 | APOX2 | L-ascorbate peroxidase | 4 | A0A3B6H3P5 | TaPRX8 | Peroxidase |
| 15 | A0A1D6CXF2 | PSMα1 | Proteasome subunit alpha type | 4 | A0A3B6QCI9 | TaPRX9 | Peroxidase |
| 15 | A0A1D5YY58 | PSMα2 | Proteasome subunit alpha type | 4 | C6ETA5 | **TaPRXⅡB** | Peroxidase |
| 15 | W5DZS4 | W5DZS4 | NAD(P)-bd_dom domain-containing protein | 2 | A0A3B5ZS22 | CYN | Cyanate hydratase |
| 15 | A0A0C4BIT2 | A0A0C4BIT2 | Epimerase domain-containing protein | 1 | A0A172WCB1 | COR | Cold-responsive LEA/RAB-related COR protein |
| 15 | A0A3B6H6X9 | A0A3B6H6X9 | Epimerase domain-containing protein | 1 | A0A3B5ZQ45 | TaPRX10 | Peroxidase |
| 15 | A0A3B6LQ83 | A0A3B6LQ83 | Epimerase domain-containing protein | 1 | A7VL25 | LEA1 | Group3 late embryogenesis abundant protein |
| 15 | A0A3B6KUA5 | A0A3B6KUA5 | PKS_ER domain-containing protein | 1 | A0A3B6B480 | WCOR14 | Cold-responsive protein WCOR14 |
| 15 | A0A3B6B107 | IGPS | Indole-3-glycerol-phosphate synthase | 1 | A0A3B6JP20 | LEA2 | Group3 late embryogenesis abundant protein |
| 14 | A0A3B6TM18 | PPI | Peptidylprolyl isomerase | 1 | A0A3B6JR58 | WCOR15 | Cold acclimation protein WCOR615 |
| 13 | A0A1D5UGI9 | TaPRX2 | Putative oxidoreductase | 1 | A0A3B6IWH4 | WCOR16 | Cold acclimation protein WCOR615 |
| 13 | Q43199 | APRT1 | Adenine phosphoribosyltransferase 1 |  |  |  |  |
