# Supplementary material for: Global Crotonylome Profiling Identifies TaPRXIIB Crotonylation as a Modulator H2O2 Homeostasis in Wheat Resistance to Puccinia triticina
Source: Mol Plant Pathol. 2026 Jul 11;27(7):e70288. doi: 10.1111/mpp.70288 (PMC13354946; doi:10.1111/mpp.70288)
Supplement: Supplementary file 11 — Table S5: Information on the interaction network of differentially modified antioxidant enzymes and their interactors. [file MPP-27-e70288-s010.docx]

| **Table S5 Information on the interaction network of differentially modified antioxidant enzymes and their interactors** | | | | | | | |
| --- | --- | --- | --- | --- | --- | --- | --- |
| Category | Accession | Rename | Description | Category | Accession | Rename | Description |
| Disease-related | Q6W8Q2 | PER1 | 1-Cys peroxiredoxin PER1 | Disease-related | W5D122 | OxR | Putative oxidoreductase GLYR1 |
| Disease-related | P80602 | BAS1 | 2-Cys peroxiredoxin BAS1 | Disease-related | F1DKC1 | **TaCAT2** | Catalase |
| Disease-related | A0A3B6I3Z5 | ACO1 | Acyl-coenzyme A oxidase | Disease-related | A0A3B6EFJ3 | GPX | Glutaredoxin-dependent peroxiredoxin |
| Disease-related | A0A3B6R6T1 | ACO2 | Acyl-coenzyme A oxidase | Stress-related | W5FUB4 | GLDH1 | L-galactono-1,4-lactone dehydrogenase |
| Disease-related | A0A3B6TAZ0 | ACO3 | Acyl-coenzyme A oxidase | Stress-related | W5FJN8 | GLDH2 | L-galactono-1,4-lactone dehydrogenase |
| Disease-related | A0A3B6SHQ4 | AGT1 | Alanine-glyoxylate aminotransferase | Stress-related | A0A3B6B286 | MS | Malate synthase |
| Disease-related | A0A3B5XYW1 | CYPs1 | Belongs to the cytochrome P450 family | Stress-related | A0A3B6H4Q4 | A0A3B6H4Q4 | PKS_ER domain-containing protein |
| Disease-related | A0A3B5YWL6 | CYPs2 | Belongs to the cytochrome P450 family | Stress-related | A0A3B6FVU5 | A0A3B6FVU5 | PKS_ER domain-containing protein |
| Disease-related | A0A3B5ZT38 | CYPs3 | Belongs to the cytochrome P450 family | Stress-related | A0A3B6EPI5 | A0A3B6EPI5 | PKS_ER domain-containing protein |
| Disease-related | A0A3B6GLK2 | CYPs4 | Belongs to the cytochrome P450 family | Stress-related | A0A3B6SIG0 | A0A3B6SIG0 | PKS_ER domain-containing protein |
| Disease-related | A0A3B5XVD9 | GSTs1 | Belongs to the GST superfamily | Stress-related | A0A077S262 | PAP | Plastid lipid-associated protein/fibrillin conserved domain-containing protein |
| Disease-related | A0A3B6TNN8 | GSTs2 | Belongs to the GST superfamily | Stress-related | A0A0C4BK42 | RPL37-1 | Ribosomal protein L37 |
| Disease-related | A0A3B6RG17 | GSTs3 | Belongs to the GST superfamily | Stress-related | A0A1D6BC85 | RPL37-2 | Ribosomal protein L37 |
| Disease-related | A0A3B6SGA5 | GSTs4 | Belongs to the GST superfamily | Stress-related | A0A3B6NLV6 | SDH1 | Succinate-semialdehyde dehydrogenase |
| Disease-related | A0A3B5YRG3 | GSTs5 | Belongs to the GST superfamily | Stress-related | A0A3B6PJL2 | SDH2 | Succinate-semialdehyde dehydrogenase |
| Disease-related | A0A3B6C474 | ICL1 | isocitrate lyase | Stress-related | A0A3B6QCZ1 | SDH3 | Succinate-semialdehyde dehydrogenase |
| Disease-related | A0A3B6DB69 | ICL2 | Isocitrate lyase | Interactors | A0A3B6HW46 | HDR1 | 4-hydroxy-3-methylbut-2-enyl diphosphate reductase |
| Disease-related | D2KZ15 | ICL3 | Isocitrate lyase | Interactors | A0A3B6ILZ0 | HDR3 | 4-hydroxy-3-methylbut-2-enyl diphosphate reductase |
| Disease-related | A0A3B5ZPY4 | NPC1 | Nuclear pore complex protein | Interactors | A0A3B6JDC9 | HDR2 | 4-hydroxy-3-methylbut-2-enyl diphosphate reductase |
| Disease-related | A0A3B5YT00 | NPC2 | Nuclear pore complex protein | Interactors | A0A3B5XVT1 | A0A3B5XVT1 | CYTOSOL_AP domain-containing protein |
| Disease-related | A0A3B5XU20 | NPC3 | Nuclear pore complex protein | Interactors | A0A3B5YU68 | A0A3B5YU68 | CYTOSOL_AP domain-containing protein |
| Disease-related | A0A3B5YTC1 | TaPRX11 | Peroxidase | Interactors | A0A3B6KGF2 | A0A3B6KGF2 | FAD-binding PCMH-type domain-containing protein |
| Disease-related | A0A3B6NXB6 | APOX2 | PEROXIDASE_4 domain-containing protein | Interactors | A0A3B6HSW1 | A0A3B6HSW1 | FAD-binding PCMH-type domain-containing protein |
| Disease-related | A0A3B6PVU5 | APOX3 | PEROXIDASE_4 domain-containing protein | Interactors | A0A3B6JMI1 | A0A3B6JMI1 | FAD-binding PCMH-type domain-containing protein |
| Disease-related | A0A3B6CC52 | TaPRX5 | PEROXIDASE_4 domain-containing protein | Interactors | A0A3B5Y6U3 | A0A3B5Y6U3 | FAD-binding PCMH-type domain-containing protein |
| Disease-related | A0A3B5ZQ45 | TaPRX10 | Peroxidase | Interactors | A0A077RTG0 | A0A077RTG0 | FAD-binding PCMH-type domain-containing protein |
| Disease-related | A0A3B6DPL3 | TaPRX3 | Peroxidase | Interactors | A0A3B6B565 | A0A3B6B565 | FMN hydroxy acid dehydrogenase domain-containing protein |
| Disease-related | A0A3B6B887 | TaPRX4 | Peroxidase | Interactors | A0A3B6LUD5 | A0A3B6LUD5 | FMN hydroxy acid dehydrogenase domain-containing protein |
| Disease-related | A0A3B6PH99 | TaPRX6 | Peroxidase | Interactors | A0A3B6C505 | A0A3B6C505 | FMN hydroxy acid dehydrogenase domain-containing protein |
| Disease-related | A0A3B6CH98 | TaPRX7 | Peroxidase | Interactors | A0A3B6DN81 | A0A3B6DN81 | FMN hydroxy acid dehydrogenase domain-containing protein |
| Disease-related | A0A3B6H3P5 | TaPRX8 | Peroxidase | Interactors | A0A3B6CD34 | A0A3B6CD34 | FMN hydroxy acid dehydrogenase domain-containing protein |
| Disease-related | A0A3B6QCI9 | TaPRX9 | Peroxidase | Interactors | A0A3B5XWY3 | HPRT1 | Hypoxanthine phosphoribosyltransferase |
| Disease-related | C6ETA5 | **TaPRXⅡB** | Peroxidase | Interactors | A0A3B5YVK4 | HPRT2 | Hypoxanthine phosphoribosyltransferase |
| Disease-related | D0PRB4 | TaPRX12 | Peroxiredoxin | Interactors | A0A3B5ZSJ6 | HPRT3 | Hypoxanthine phosphoribosyltransferase |
| Disease-related | Q5S1S6 | TaPRX1 | Peroxiredoxin Q, chloroplastic | Interactors | A0A3B5Y4I8 | A0A3B5Y4I8 | MaoC-like domain-containing protein |
| Disease-related | A0A1D5UGI9 | TaPRX2 | Putative oxidoreductase | Interactors | A0A077RXD9 | MED1 | Mediator of RNA polymerase II transcription subunit 10 |
| Disease-related | A0A3B5YYW5 | RINGE3s-1 | RING-type E3 ubiquitin transferase | Interactors | A0A077S2Q2 | MED2 | Mediator of RNA polymerase II transcription subunit 10 |
| Disease-related | A0A3B5ZX64 | RINGE3s-2 | RING-type E3 ubiquitin transferase | Interactors | A0A3B5Y074 | MED3 | Mediator of RNA polymerase II transcription subunit 31 |
| Disease-related | A0A3B5Y1M2 | RINGE3s-3 | RING-type E3 ubiquitin transferase | Interactors | A0A3B6LW64 | MTHFR1 | Methylenetetrahydrofolate reductase |
| Disease-related | A0A3B5XWS0 | SPP1 | Serine/threonine-protein phosphatase | Interactors | W5FX66 | MTHFR2 | Methylenetetrahydrofolate reductase |
| Disease-related | A0A3B5YTD5 | SPP2 | Serine/threonine-protein phosphatase | Interactors | A0A3B6QFE4 | NAXE1 | NAD(P)H-hydrate epimerase |
| Disease-related | A0A3B6KCU5 | SPP3 | Serine/threonine-protein phosphatase | Interactors | A0A3B6NR76 | NAXE2 | NAD(P)H-hydrate epimerase |
| Disease-related | A0A3B6LFS8 | SPP4 | Serine/threonine-protein phosphatase | Interactors | A0A3B6PL53 | NAXE3 | NAD(P)H-hydrate epimerase |
| Disease-related | A0A3B6MLJ6 | SPP5 | Serine/threonine-protein phosphatase | Interactors | A0A3B5YXB7 | A0A3B5YXB7 | Protein kinase domain-containing protein |
| Disease-related | A0A0C4BJ55 | TRX6 | Thioredoxin domain-containing protein | Interactors | A0A077RV00 | SURF5 | Surfeit locus protein 5 |
| Disease-related | A0A3B6B0I4 | TRX7 | Thioredoxin domain-containing protein | Interactors | A0A341ZLT5 | A0A341ZLT5 | Uncharacterized protein |
| Disease-related | A0A3B6C702 | TRX8 | Thioredoxin domain-containing protein | Interactors | A0A3B5XT95 | A0A3B5XT95 | Uncharacterized protein |
| Disease-related | A0A3B6LHJ4 | SUMO1-1 | Ubiquitin-related modifier 1 | Interactors | A0A1D5UPR1 | A0A1D5UPR1 | Uncharacterized protein |
| Disease-related | A0A3B6IWZ7 | SUMO1-2 | Ubiquitin-related modifier 1 homolog | Interactors | A0A3B5YQ78 | A0A3B5YQ78 | Uncharacterized protein |
| Disease-related | A0A3B6MM80 | SUMO1-3 | Ubiquitin-related modifier 1 homolog | Interactors | A0A3B6GWI2 | A0A3B6GWI2 | WD_REPEATS_REGION domain-containing protein |
| Disease-related | A0A3B6KB43 | SUMO1-4 | Ubiquitin-related modifier 1 homolog | Interactors | A0A3B6EK60 | A0A3B6EK60 | WD_REPEATS_REGION domain-containing protein |
