# Supplementary material for: Global Crotonylome Profiling Identifies TaPRXIIB Crotonylation as a Modulator H2O2 Homeostasis in Wheat Resistance to Puccinia triticina
Source: Mol Plant Pathol. 2026 Jul 11;27(7):e70288. doi: 10.1111/mpp.70288 (PMC13354946; doi:10.1111/mpp.70288)
Supplement: Supplementary file 12 — Table S6: Real‐time quantitative PCR primers and related information used for gene detection. [file MPP-27-e70288-s013.docx]

| **Table S6 Real-time quantitative primers and related information used for gene detection** | | | | |
| --- | --- | --- | --- | --- |
| Gene name | Accession | Forward/Reverse: sequence (5'->3') | Length/nt | Notes |
| *TaPR1* | KF196296.1 | F: ACAGAAACCATACTAACTGC | 20 | Pathogenesis-related gene |
|  |  | R: AGCCCCTCTAGCTAGTTACAG | 21 |  |
| *TaPR2* | AK446628.1 | F: CCGCACAAGACACCTCAAGATA | 22 | Pathogenesis-related gene |
|  |  | R: CGATGCCCTTGGTTTGGTAGA | 21 |  |
| *TaPR5* | KJ764822.1 | F: AGACTTCTACGACATCTCG | 19 | Pathogenesis-related gene |
|  |  | R: TGCAGGCGTGTGTGGCGAC | 19 |  |
| *TaPRXⅡB* | C6ETA5 | F: TCGACGTTCTACGACACGTC | 20 | Key differentially modified protein gene |
|  |  | R: GGACCCGCGTTTTGTTCCA | 19 |  |
| *TaCAT2* | F1DKC1 | F: CAGCTTCGACACCAAGACGA | 20 | Key differentially modified protein gene |
|  |  | R: AAGTGGGCGATCTTCTCCAG | 20 |  |
| *TaOMT* | Q84N28 | F: GAAGTGGATCCTCCACGACT | 20 | Key differentially modified protein gene |
|  |  | R: AAGTGGTCTTGATGGCTTTGA | 21 |  |
| *TaArf* | Q76ME3 | F: AGGGATGAGCTCCACAGGAT | 20 | Key differentially modified protein gene |
|  |  | R: GTCCCTCGTACAACCCTTCG | 20 |  |
| *TaPGM* | W5D322 | F: ACTTTGACGAGACCAGGGAA | 20 | Key differentially modified protein gene |
|  |  | R: TGGTCAAACTTCCCACTGAGG | 21 |  |
| *TaPLD* | W5D4Q6 | F: ATGTGGCCTGAGGGTGTTC | 19 | Key differentially modified protein gene |
|  |  | R: CTAGCCTCTTGAGCACGGAC | 20 |  |
| *TaRPS7* | Q5I7K2 | F: GAGCTCAAGAGCGACCTCAA | 20 | Key differentially modified protein gene |
|  |  | R: ATGAACAGCTGTCAGGGTCC | 20 |  |
| *TaRSL2D* | W5ECL2 | F: TATGTACACCGGCCAGTTCG | 20 | Key differentially modified protein gene |
|  |  | R: TTGGGACGATCTTCTTGGCG | 20 |  |
| *TaH2A* | A0A1D5YKH2 | F: TACTGCGGCGGTCTACTCTG | 20 | Key differentially modified protein gene |
|  |  | R: ATGTGCGGGATCACACCTC | 19 |  |
| *TaRPL22* | Q95H48 | F: GTGGGCTAAGGAAACTCGCAA | 21 | Key differentially modified protein gene |
|  |  | R: CGATAGGGCATGAGGTTCAGT | 21 |  |
| *TaDLD* | W5A874 | F: ACTGGGGCTCTTGCACTTAC | 20 | Key differentially modified protein gene |
|  |  | R: CCAGAGGTATCGACCCCAAC | 20 |  |
| *TaPFDNB* | W5BRT4 | F: GCGGAGTCTTGGTTGAGAGG | 20 | Key differentially modified protein gene |
|  |  | R: CCGCAAGGCCAACAAGAAC | 19 |  |
| *TaEF-1α* | M90077.1 | F: TGGTGTCATCAAGCCTGGTATGGT | 24 | Reference gene |
|  |  | R: ACTCATGGTGCATCTCAACGGACT | 24 |  |
| *TaPRXⅡB_Rg* | A0A3B6D745 | F: CCCCGACGTTCTACAGC | 17 | The highest sequence similarity to *TaPRXⅡB* |
|  |  | R: TTTGCACCTGTGGAGTCCC | 19 |  |
