# Supplementary material for: Global Crotonylome Profiling Identifies TaPRXIIB Crotonylation as a Modulator H2O2 Homeostasis in Wheat Resistance to Puccinia triticina
Source: Mol Plant Pathol. 2026 Jul 11;27(7):e70288. doi: 10.1111/mpp.70288 (PMC13354946; doi:10.1111/mpp.70288)
Supplement: Supplementary file 13 — Table S7: Information on primers for full‐length gene cloning, site‐directed mutagenesis and overexpression. [file MPP-27-e70288-s001.docx]

| **Table S7 Information on primers for full-length gene cloning, site-directed mutagenesis, and overexpression** | | | | |
| --- | --- | --- | --- | --- |
| Gene name | Accession | Forward/Reverse: sequence (5'->3') | Length/nt | Notes |
| *TaPRXⅡB* | C6ETA5 | F: ATGGCCATGGGTTCTGCTTC | 20 | Full-length |
|  |  | R: TCACGAGTTCACCTTGGAGC | 20 |  |
| *TaCAT2* | F1DKC1 | F: ATGGATCCCTGCAAGTTCCG | 20 | Full-length |
|  |  | R: TCACATGCTTGGCTTCACGTTAAG | 24 |  |
| *TaPRXⅡB* | C6ETA5 | F: CTCGAGGCTGCGTTCCTC**CAG**AAGAACCTTAACACGGTC | 39 | K172Q |
|  |  | R: GACCGTGTTAAGGTTCTT**CTG**GAGGAACGCAGCCTCGAG | 39 |  |
| *TaPRXⅡB* | C6ETA5 | F: CTCGAGGCTGCGTTCCTC**CGC**AAGAACCTTAACACGGTC | 39 | K172R |
|  |  | R: GACCGTGTTAAGGTTCTT**GCG**GAGGAACGCAGCCTCGAG | 39 |  |
| *TaCAT2* | F1DKC1 | F: CCGCGCGGGTTCGCCGTC**CAG**TTCTACACGCGCGAGGGC | 39 | K125Q |
|  |  | R: GCCCTCGCGCGTGTAGAA**CTG**GACGGCGAACCCGCGCGG | 39 |  |
| *TaCAT2* | F1DKC1 | F: CCGCGCGGGTTCGCCGTC**CGC**TTCTACACGCGCGAGGGC | 39 | K125R |
|  |  | R: GCCCTCGCGCGTGTAGAA**GCG**GACGGCGAACCCGCGCGG | 39 |  |
| *TaCAT2* | F1DKC1 | F: GAGAAGACGAGGATCAAG**CAG**GAGAACGACTTCGTGCAG | 39 | K426Q |
|  |  | R: CTGCACGAAGTCGTTCTC**CTG**CTTGATCCTCGTCTTCTC | 39 |  |
| *TaCAT2* | F1DKC1 | F: GAGAAGACGAGGATCAAG**CGC**GAGAACGACTTCGTGCAG | 39 | K426R |
|  |  | R: CTGCACGAAGTCGTTCTC**GCG**CTTGATCCTCGTCTTCTC | 39 |  |
| *TaPRXⅡB* | C6ETA5 | F: tagcgctaccggtcgATGGCCATGGGTTCTGCTTCTT | 37 | pCamA:: *Gene* |
|  |  | R: cccggtacccggggaCGAGTTCACCTTGGAGCAG | 34 |  |
| *TaCAT2* | F1DKC1 | F: tagcgctaccggtcgATGGATCCCTGCAAGTTCCG | 35 | pCamA::*Gene* |
|  |  | R: cccggtacccggggaCATGCTTGGCTTCACGTTAAGG | 37 |  |
