# Supplementary material for: Global Crotonylome Profiling Identifies TaPRXIIB Crotonylation as a Modulator H2O2 Homeostasis in Wheat Resistance to Puccinia triticina
Source: Mol Plant Pathol. 2026 Jul 11;27(7):e70288. doi: 10.1111/mpp.70288 (PMC13354946; doi:10.1111/mpp.70288)
Supplement: Supplementary file 14 — Table S8: Information on primers for BMSV vector construction and gene silencing efficiency assessment. [file MPP-27-e70288-s008.docx]

| **Table S8 Information on primers for BMSV vector construction and gene silencing efficiency assessment** | | | | |
| --- | --- | --- | --- | --- |
| Gene name | Accession | Forward/Reverse: sequence (5'->3') | Length/nt | Notes |
| *TaPRXⅡB* | C6ETA5 | F: AACCTTAACACGGTCGACATGG | 22 | Silencing fragment |
|  |  | R: CCTTTCTGTGACAGGAGGTTGGT | 23 |  |
| *TaPRXⅡB* | C6ETA5 | F: taattaacccggggcAACCTTAACACGGTCGACATGG | 37 | BMSV:*TaPRXⅡB* |
|  |  | R: gctagctgagcggccCCTTTCTGTGACAGGAGGTTGGT | 38 |  |
| *TaPRXⅡB* | C6ETA5 | F: TCGACGTTCTACGACACGTC | 20 | Silencing efficiency detection |
|  |  | R: GGACCCGCGTTTTGTTCCA | 19 |  |
